# All-Hydrocarbon-Ligated Superatomic Gold/Aluminum Clusters

**DOI:** 10.1021/acs.inorgchem.3c03790

**Published:** 2024-02-09

**Authors:** Ivan Antsiburov, Max Schütz, Raphael Bühler, Maximilian Muhr, Johannes Stephan, Christian Gemel, Wilhelm Klein, Samia Kahlal, Jean-Yves Saillard, Roland A. Fischer

**Affiliations:** † Department of Chemistry and Catalysis Research Center, Chair of Inorganic and Metal−Organic Chemistry, Technical University of Munich, Lichtenbergstr. 4, Munich, Garching 85748, Germany; ‡ Univ Rennes, CNRS, ISCR-UMR 6226, Beaulieu, Rennes F-35000, France

## Abstract

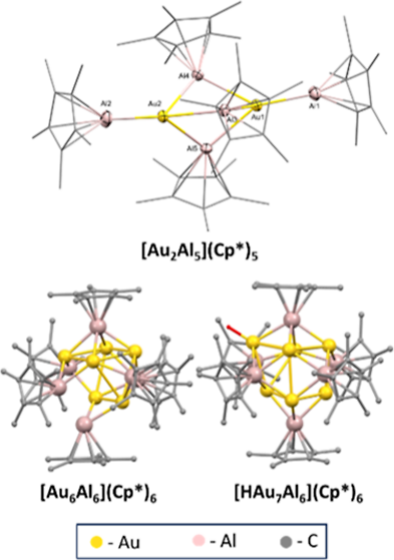

Key strategies in cluster synthesis include the use of modulating agents (e.g., coordinating additives). We studied the influence of various phosphines exhibiting different steric and electronic properties on the reduction of the Au(I) precursor to Au(0) clusters. We report a synthesis of the bimetallic clusters [Au_6_(AlCp*)_6_] = [Au_6_Al_6_](Cp*)_6_ (**1**) and [HAu_7_(AlCp*)_6_] = [HAu_7_Al_6_](Cp*)_6_ (**2**) (Cp* = pentamethylcyclopentadiene) using Au(I) precursors and AlCp*. The cluster [Au_2_(AlCp*)_5_] = [Au_2_Al_5_](Cp*)_5_ (**3**) was isolated and identified as an intermediate species in the reactions to **1** and **2**. The processes of cluster growth and degradation were investigated by in situ ^1^H NMR and LIFDI-MS techniques. The structures of **1** and **2** were established by DFT geometry optimization. These octahedral clusters can both be described as closed-shell 18-electron superatoms.

## Abstract 2

Two novel intermetalloid Au/Al clusters were synthesized with phosphines as modulators accomplishing atom precision in the cluster growth process. A small Au/Al cluster was selectively formed during cluster degradation.

## Introduction

Gold clusters have been known for more than 50 years and are among the most thoroughly studied metal clusters.^1−5^ The investigation of their structures, electronic properties, and reactivity profiles contributed a lot to the general understanding of cluster chemistry and the nature of metal–metal bonding.^6−8^ The physical and chemical properties of a cluster are determined by its ligand shell and the precise number of metal atoms in the core.^9^ The most widely studied are gold clusters bearing phosphines or thiolate ligands, such as the famous *Schmid cluster* [Au_55_](PPh_3_)_12_Cl_6_ or the thiolate-stabilized Au_25_(SR)_18_.^2,10,11^ Such clusters are typically obtained by the reduction of Au(I) or Au(III) precursors, e.g., [AuCl_4_]^−^ or (R_3_P)AuCl, in the presence of stabilizing and cluster growth-controlling phosphine or thiolate ligands. However, the precise size-selectivity (atomicity and nuclearity) of the cluster synthesis is most often impossible.^10^ Common reducing agents for the Au precursors used in the synthesis of gold clusters and nanoparticles are borohydrides, carboxylic acids, or polyols, among many others.^12^

Usually, these reducing agents serve as a source of electrons without providing additional stabilizing interactions with the formed product clusters. A different approach combining a reducing and cluster stabilizing agent in one molecule is, for example, the low valent group 13 organometallic compounds ECp* (E = Al and Ga).^13−16^ The reaction of AlCp* with {CuMes} under various conditions, for example, leads to the formation of clusters [Cu_7_Al_6_](Cp*)_6_, [Hcu_7_Al_6_](Cp*)_6_, [Cu_8_Al_6_](Cp*)_6_, and [Cu_43_Al_12_](Cp*)_12_. In these reactions, Cu(I) is reduced to Cu(0) by AlCp*, AlMes_3_, and AlMes_2_Cp* arising as oxidized side products. In a similar manner, the reaction of AlCp* with {(PPh_3_)CuH}_6_ gives the cluster [H_4_Cu_6_Al_6_](Cp*)_6_. In this case, the elimination of H_2_ is also observed as a parallel pathway for the reduction of Cu(I). The notation of cluster formulas that we prefer highlights the hydrocarbon protected bimetallic core structure, including hydrides [H_*x*_Au_a_Al_b_](Cp*)_b_, rather than specifying the selective binding of Cp* to Al and implying Cp*Al acting as a protecting ligand for the cluster core, which is highlighted by the alternative notation [H_*x*_Au_a_(AlCp*)_b_].

In this work, we describe the synthetic access to the three new gold–aluminum clusters [Au_6_Al_6_](Cp*)_6_ (**1**), [HAu_7_Al_6_](Cp*)_6_ (**2**), and [Au_2_Al_5_](Cp*)_5_ (**3**). Heteronuclear gold clusters with late transition metals (e.g., Pd and Pt) were studied intensively in the past and show promising reactivities, such as the activation of H_2_.^17^ In the contrary, intermetallic gold clusters with p-block metals received considerably less attention, with only a few examples known in the literature.^18−20^ The initial reaction of the precursors ^*i*^DippAuH (^*i*^Dipp = 2,3-dihydro-1,3-bis(2,6-diisopropylphenyl)-1H-imidazole-2-ylidene) and AlCp* (Cp* = η^5^-C_5_Me_5_) leads to a library of various Au/Al clusters of different nuclearities. Inspired by similar concepts in nanoparticle synthesis,^21−24^ we recently showed that the product distribution in the synthesis of related Ni/Ga and Ni/Al clusters can be narrowed down to singular products by using alkynes as additives during synthesis (coordination modulation).^25^ In the present case, phosphines were chosen as adequate additives due to their ability to stabilize the Au(0) centers.^26^

The geometric and electronic structures of the larger clusters [Au_6_Al_6_](Cp*)_6_ (**1**) and [HAu_7_Al_6_](Cp*)_6_ (**2**) were investigated by calculations on the density functional theory (DFT) level of theory, revealing the bonding situations in **1** and **2** related to the corresponding hydrocarbon-ligated (organometallic) Cu/Al superatomic clusters.

## Experimental Section

### General

All experiments were conducted using standard Schlenk and glovebox techniques under an atmosphere of purified argon. **Caution!** Extreme care should be taken in both the handling of the cryogen liquid nitrogen and its use in the Schlenk line trap to avoid the condensation of oxygen from air. **Caution!** Gold/aluminum clusters are air-sensitive and pyrophoric. Their residues should be quenched with isopropanol. All solvents were carefully dried over molecular sieves (water content <5 ppm) and degassed prior to their use. The starting compounds AlCp* and ^*i*^DippAuH (^*i*^Dipp = 1,3-bis(2,6-diisopropylphenyl)imidazole-2-ylidene) were prepared according to the literature methods.^27,28^ For **1**, and the cocrystal **1**/**2** no meaningful data for the elemental analysis (C, H, Au, and Al) could be obtained. Satisfying elemental analysis data for pure isolated compounds **2** and **3** were obtained. However, based on the spectroscopic data of pure **2**, the assignments of LIFDI MS^29^ and ^1^H NMR data for **1** and **1/2** were possible. NMR spectra were recorded on a Bruker Avance III AV400US (^1^H, 400 MHz; ^13^C, 101 MHz). Chemical shifts are described in parts per million relative to tetramethylsilane (TMS) and referenced to the solvent residual signals. FTIR spectra were measured with an ATR setup using a Bruker Alpha FTIR spectrometer under an inert gas atmosphere in a glovebox. UV–vis spectra were measured in toluene under an inert atmosphere with an Agilent Carry 60 spectrometer. **Caution!** All chemicals should be handled with care and personal protective equipment must be used.

### Synthesis of [Au_6_Al_6_](Cp*)_6_ (1)

Samples of (PPh_3_)AuCp* (305 mg, 538 μmol, 1.0 equiv) and AlCp* (125 mg, 807 μmol, 1.5 equiv) were heated in 30 mL of toluene at 75 °C for 2 h. The hot, orange reaction solution was filtered and concentrated in vacuo to 12 mL. The resulting solution was stored at −32 °C for 7 days and at −86 °C for 4 days. The formed brown crystalline precipitate was filtered off, washed with hexane (3 × 5 mL) and with pentane (4 × 2 mL), and dried in vacuo, giving pure **1** (52.4 mg, 24.3 μmol, 27% based on Au). **Caution!** Compound **1** is air-sensitive and pyrophoric. Its residues were quenched with isopropanol.

^1^H NMR (400 MHz, Benzene-*d*_6_) δ 1.94 (s, 90H).

^13^C NMR (126 MHz, Benzene-*d*_6_) δ 115.21 (s, quaternary *C* (Cp*)), 13.00 (s, *C*H_3_).

IR (ATR, 298 K): ν [cm^–1^] = 2907 (m), 2849 (m), 2361 (w), 2340 (w), 1425 (s), 1370 (s), 795 (s), 727 (s), 422 (vs) cm^–1^.

UV–vis (298 K, toluene): 513 (broad), 385–400 (shoulder, strong), 360–380 (shoulder, small), 307 nm (sharp).

LIFDI-MS: *m*/*z* = 2155 ([Au_6_Al_6_](Cp*)_6_]^+^).

No satisfying elemental analysis data of **1** could be obtained due to the high sensitivity of the cluster.

### Synthesis of [HAu_7_Al_6_](Cp*)_6_ (2)

Samples of ^*i*^DippAuH (150 mg, 256 μmol, 1.0 equiv), AlCp* (31 mg, 192 μmol, 0.75 equiv), and PPh_3_ (201 mg, 767 μmol, 3.0 equiv) were heated in 15 mL of toluene at 75 °C for 3 h. The hot, dark-red reaction solution was filtered and stored at −32 °C for 7 days. The formed dark crystalline precipitate was filtered off, washed with pentane (2 × 5 mL), then with THF (3 × 1 mL), and again with pentane (3 × 1 mL) and dried in a glovebox, giving the pure cluster **2** (22 mg, 9.3 μmol, 26% based on Au). **Caution!** Compound **2** is air-sensitive and pyrophoric. Its residues were quenched with isopropanol.

^1^H NMR (298 K, 400 MHz, C_6_D_6_): 1.99 (s (broad), 90H, C*H*_3_ (Cp*)).

^13^C NMR (298 K, 500 MHz, cryo-probe, C_6_D_6_): 115.5 (s, quaternary *C* (Cp*)), 12.9 (s, *C*H_3_).

IR (ATR, 298 K): ν [cm^–1^] = 3552 (w), 3483 (w), 2964 (w), 2902, 2850, 1753, 1452 (shoulder), 1421, 1371, 1310 (b, (i), 1050 (w), 1027 (w), 801, 729, 694, 587, 451 (i).

UV–vis (298 K, toluene): 514 nm (broad), 480 nm (very weak), 415–463 nm (shoulder, strong), 298 nm (sharp).

LIFDI-MS: *m*/*z* = 2351 ([Au_7_Al_6_](Cp*)_6_]^+^).

Elemental analysis [%]: calculated for Au_7_Al_6_C_60_H_90_: C: 30.64, H: 3.86, Al: 6.88, Au: 58.62; found: C: 29.95, H: 3.88, Al: 6.73, Au: 58.41.

### Synthesis of [DAu_7_Al_6_](Cp*)_6_ (2D)

Samples of ^*i*^DippAuD (125 mg, 213 μmol, 1.0 equiv), AlCp* (26 mg, 160 μmol, 0.75 equiv), and PPh_3_ (168 mg, 638 μmol, 3.0 equiv) were heated in 15 mL of toluene at 75 °C for 3 h. The hot, dark-red reaction solution was filtered and stored at −32 °C for 7 days. The formed dark crystalline precipitate was filtered off, washed with pentane (2 × 5 mL), then with THF (3 × 1 mL), and again with pentane (3 × 1 mL) and dried in a glovebox, giving the pure cluster **2D** (15 mg, 6.4 μmol, 24% based on Au). **Caution!** Compound **2D** is air-sensitive and pyrophoric. Its residues were quenched with isopropanol.

^1^H NMR (298 K, 400 MHz, C_6_D_6_): 1.99 [s (broad), 90H, C*H*_3_ (Cp*)].

IR (ATR, 298 K): ν [cm^–1^] = 3552 (w), 3483 (w), 2964 (w), 2902, 2850, 1452 (shoulder), 1421, 1371, 1310 b, (i), 1260 (i), 1050 (w), 1027 (w), 801, 729, 694, 587, 451 (i).

LIFDI-MS: *m*/*z* = 2351 ([Au_7_Al_6_](Cp*)_6_]^+^).

### Synthesis of [H_0_–_1_Au_6/7_Al_6_](Cp*)_6_ (1/2)

Samples of ^*i*^DippAuH (150 mg, 0.256 mmol, 1.0 equiv) and AlCp* (41.56 mg, 0.256 mmol 1.0 equiv) were heated in 15 mL of toluene at 75 °C for 3 h. After a short time, some gas evolution was observed. The dark-red reaction solution was filtered and stored at −30 °C for several weeks. The formed dark crystalline precipitate was isolated by means of filtration and washed with *n*-hexane (3 × 0.1 mL) and benzene (3 × 0.1 mL) resulting in pure **1/2** (10 mg, 4.4 μmol, 11% based on Au). **Caution!** Compound **1/2** is air-sensitive and pyrophoric. Its residues were quenched with isopropanol.

^1^H NMR (298 K, 400 MHz, C_6_D_6_): 1.99 [s (broad), 90H, C*H*_3_ (Cp*) of **2**], 1.94 [s (broad), 90H, C*H*_3_ (Cp*) of **1**].

^13^C NMR (298 K, 500 MHz, cryo-probe, C_6_D_6_): 12.99 [s, *C*H_3_ (Cp*) of **1**], 115.2 [s, quaternary *C* (Cp*) of **1**], 115.5 [s, quaternary *C* (Cp*) of **2**].

IR (ATR, 298 K): ν [cm^–1^] = 2959 (w), 2904, 2845, 1756 (w), 1595, 1411, 1358, 1325, 1264, 1230, 1201, 1158–915 b, (i), 889, 809, 781, 742, 685, 638, 546, 426.

UV–vis (298 K, toluene): 513 (broad), 478 (very weak), 440 (shoulder, weak), 309 nm (sharp).

LIFDI-MS: *m*/*z* = 2155 ([Au_6_Al_6_](Cp*)_6_]^+^), 2351 ([Au_7_Al_6_](Cp*)_6_]^+^).

### Synthesis of [Au_2_Al_5_](Cp*)_5_ (3)

Samples of ^*i*^DippAuH (100 mg, 170 μmol 1.0 equiv) and AlCp* (69 mg, 426 μmol, 2.5 equiv) were heated in 5 mL of toluene to 75 °C for 20 h. The hot, dark–brown reaction solution was filtered and stored at −32 °C for 2 weeks. The formed dark needles were filtered off, washed with pentane (5 × 0.1 mL), then dissolved in 2.5 mL hexane at 70 °C and stored at −32 °C for 6 days. Crystals were isolated by decantation, washed with cold pentane (3 × 0.2 mL), and dried in a glovebox, giving the pure cluster 3 (18.5 mg, 15.4 μmol, 18% based on Au). **Caution!** Compound 3 is air-sensitive and pyrophoric. Its residues were quenched with isopropanol.

^1^H NMR (298 K, 400 MHz, C_6_D_6_): 2.19 [s (very broad), 45H, bridging AlCp*]; 1.85 [s (very broad), 30H, terminal AlCp*].

^13^C NMR (298 K, 500 MHz, cryo-probe, C_7_D_8_): 114.19 (s, quaternary C), 13.65 (s, -*C*H_3_), 10.23 (s, -*C*H_3_).

LIFDI-MS: *m*/*z* = 1203 ([**3**-H]^+^).

IR (ATR, 298 K): ν [cm^–1^] = 2964 (w), 2900, 2848, 2712 (w), 1487 (w), 1417, 1369, 1176 (w), 1019, 799, 578, 455 (i).

UV–vis (298 K, toluene): 498–536 nm (weak, broad), 478 nm (broad), 406 nm (very broad), 331 nm (sharp).

Elemental analysis [%]: calculated for Au_2_Al_5_C_50_H_75_: C: 49.84, H: 6.27, Al: 11.20, Au: 32.69; found: C: 49.00, H: 6.22, Al: 10.87, Au: 32.51.

### Computational Details

DFT calculations^30^ were carried out with the use of the Amsterdam Density Functional code (ADF2019)^31−33^ incorporating scalar relativistic corrections via the ZORA Hamiltonian^34^ and with the addition of Grimme’s D3(BJ) empirical corrections^35^ in order to take into account dispersion effects. The all-electron triple-ξ Slater basis set plus two polarization functions (STO-TZ2P)^36^ was used, together with the Becke–Perdew (BP86)^37,38^ exchange–correlation functional. All of the optimized structures were confirmed as true minima on their potential energy surface by analytical vibration frequency calculations. Natural atomic orbital populations and Wiberg bond indices were computed with the natural bond orbital NBO6.0 program^39^ implemented in the ADF2019 package. The NMR chemical shifts were computed according to the gauge-independent atomic orbital method.^40^

Owing to the too long demand of CPU time required by the ADF2019 code for these clusters, the Gaussian 16 package^41^ was used for calculating the UV–visible electronic transitions by means of time-dependent DFT (TD-DFT) calculations. The PBE0 hybrid functional^42,43^ was chosen for the sake of accuracy, together with the Def2TZVP basis set.^44,45^ The UV–visible spectra were simulated from the computed TD-DFT transitions and their oscillator strengths, each transition being associated with a Gaussian function of half-height widths equal to 1500 cm^–1^

## Results and Discussion

### Synthesis and Spectroscopic Characterization of [Au_6_Al_6_](Cp*)_6_ (1)

**Scheme 1:**
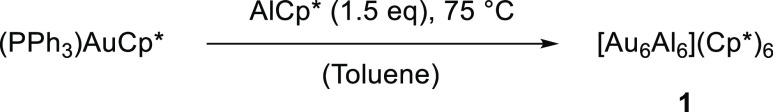
Synthesis of the [Au_6_Al_6_](Cp*)_6_ (**1**)

The cluster [Au_6_Al_6_](Cp*)_6_ (**1**) was prepared by the reaction of (PPh_3_)AuCp* with AlCp* in a 27% yield (Scheme 1). The preparative purity of isolated **1** is confirmed by ^1^H NMR analysis in benzene-d_6_, exhibiting only one peak at 1.94 ppm as the only signal, besides the very small impurities of HCp* (see Figure S1). The ^13^C NMR spectrum shows the quaternary carbon signal of the Cp* ligands of **1** at 115.21 ppm and the signal of the methyl groups at 13.00 ppm (see Figure S2). LIFDI-MS analysis of isolated samples of **1** reveals the presence of **1** together with trace amounts of **2**. Observed molecular peaks and fragments include (see Figure S9): [Au_6_Al_6_](Cp*)_5_^+^ (*m*/*z* 2019.79), {[Au_6_Al_6_](Cp*)_7_ – H}^+^ (*m*/*z* 2289.20), and {[Au_7_Al_6_](Cp*)_7_ – H}^+^ (*m*/*z* 2486.17). The ATR-IR spectrum of **1** shows the expected bands of C–H, C–C, and Al-Cp* stretching as well as C–H bending (Figure S11). Two signals at 2361 and 2340 cm^–1^ could not be assigned. The bands in this range are typical for CO_2_, but no CO_2_ was present inside the glovebox (Ar inert gas) where the sample was prepared and measured. The wavenumbers of these two signals are significantly higher than the value of 1753 cm^–1^ observed for the ν(Au–H) stretching vibration of [HAu_7_Al_6_](Cp*)_6_ (vide infra). The UV–vis spectrum of isolated **1** in toluene shows a prominent absorption band at 496 nm −540 nm (maximum: 513 nm), an intense shoulder at 385–400 nm, as well as a weak shoulder at 360–380 nm (see Figure S13).

### Synthesis and Characterization of [HAu_7_Al_6_](Cp*)_6_ (2)

**Scheme 2:**
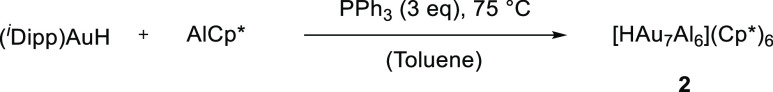
Synthesis of the [HAu_7_Al_6_](Cp*)_6_ (**2**)

The cluster [HAu_7_Al_6_](Cp*)_6_ (**2**) was prepared by the reaction of ^*i*^DippAuH with AlCp* in the presence of a high excess of PPh_3_ with a 26% yield (Scheme 2).

The reaction outcome of ^*i*^DippAuH and AlCp* in the presence of PR_3_ (10 equiv) exhibiting different electronic properties was studied by in situ ^1^H NMR spectroscopy (see Figures S33–46). In the most cases with monodentate [PPh_3_, P(*p*-Tol)_3_, P(anis)_3_, PEt_3_, P(*n*-Oct)_3_, P(*i*-Pr)_3_], and bidentate (dppbz, dppe), the selective formation of **2** is observed. The selectivity toward **2** was lower in the presence of P(4-FC_6_H_4_)_3_ and a small amount of **1** is observed. The presence of very bulky PCy_3_ or P(*o*-Tol)_3_ leads to a mixture **1**/**2**, i.e., the same reaction outcome as in the absence of any additive. The presence of small, strongly σ-donating phosphines PMe_3_ or depe led to the precipitation of elemental gold. Usage of phosphites [P(OPh)_3_ or P(OMe)_3_] leads to decomposition under P–O bond cleavage, and the phosphinidene ^*i*^Dipp = PH arises as a side product. Notably, AlCp* as well as ^*i*^DippAuH alone are unreactive toward P(OR)_3_. The reduction of Au(I) in precursor ^*i*^DippAuH to yield Au(0) in cluster **2** happens through reductive elimination of H_2_ or by reduction with AlCp*, which is confirmed by in situ NMR measurements (Figures S30 and S33).

The purity of **2** is confirmed by ^1^H NMR analysis in benzene-d_6_, exhibiting only one peak at 1.99 ppm as the only signal, besides the very small impurities of PPh_3_ and cocrystallized toluene (see Figure S4). The hydride signal could not be detected neither at room temperature nor at −80 °C. The ^13^C NMR spectrum shows the quaternary carbon signal of the Cp* ligands of **2** at 115.50 ppm (see Figure S5). The signal of -*C*H_3_ carbon atoms at 12.91 ppm could be observed only in the DEPT 135° experiment (see Figure S6). Likewise, the LIFDI-MS spectrum of isolated **2** shows the molecular ion peak of **2** as the only signal attributable to a cluster species (see Figure S10). The IR spectrum of **2** (see Figure S11) exhibits the characteristic C–H stretching (2790–3012 cm^–1^), C–H deformation (1178–1335 cm^–1^; 729 cm^–1^), and C–C stretching (1380–1497 cm^–1^) modes of the Cp* ligand are again identified in the spectrum in addition to the Al-Cp* stretching frequency at 445 cm^–1^. A characteristic ν(Au–H) stretching vibration is discernible at 1720–1780 cm^–1^. The band exhibits maximum absorption at 1753 cm^–1^, as well as two shoulders at 1735 and 1770 cm^–1^. This value is very similar to the IR data of [AuH_4_]^−^ (1676.4 cm^–1^ and 1678.8 cm^–1^) and [H_2_AuH_3_] (1651.5 cm^–1^ and 1666.8 cm^–1^) obtained in matrix isolation experiments, whereas the frequencies observed for [AuH] (2226.6 cm^–1^) and also for ^*i*^DippAuH (1976 cm^–1^) are found at higher wavenumbers.^28,46^ This assignment was confirmed through the preparation of the corresponding deuteride cluster (**2D**) from ^*i*^DippAuD (86% deuterium according to ^1^H NMR). Indeed, the band at 1753 cm^–1^ is almost vanished and a new intense band at 1260 cm^–1^ arises (Figure S12). This value is in good agreement with the computed value of 1236 cm^–1^ (Figure S61). The UV–vis spectrum of isolated **2** in toluene shows the prominent absorption band at 494 nm −540 nm (maximum: 514 nm), the minor band at 480 nm, and the shoulder at 415–463 nm (see Figure S13). Single crystals of **2** are obtained upon crystallization from toluene at −35 °C. SC-XRD analysis of these crystals leads to a highly disordered structure (i.e., cocrystallized isomers), and the data obtained upon refinement are not sufficiently accurate for a detailed discussion of structural parameters. However, the overall structural motive is confirmed by SC-XRD data analysis. It consists of a Au_7_ core, which adopts an approximate cube-shaped geometry with one missing vertex. This unit is embedded in an octahedral Al_6_ shell (see Figure S58).

### Computational DFT Investigation

In order to shed some light on the structure, bonding, and stability of clusters **1** and **2**, we investigated them through DFT calculations at the BP86/TZ2P-D3 level (see Computational Details). The experimental, solid-state structure of **2**, somewhat reminds that of the copper–aluminum clusters [HCu_7_Al_6_](Cp*)_6_ and [Cu_8_Al_6_](Cp*)_6_, which cocrystallize too.^15^ In any case, the structures derived from analysis of XRD data suggest an *M*_n_ (M = Au and Cu) core embedded within a distorted (AlCp*)_6_ octahedron and DFT calculations confirmed this type of arrangement for [HCu_7_Al_6_](Cp*)_6_ and [Cu_8_Al_6_](Cp*)_6_.^15^ When looking for structures of this type in the case of **2**, four low-energy isomers were found, lying within a range of 5 kcal/mol (Figure S59). The most stable, both in total (E) and free (G) energies, is shown in Figure S59 and selected computed data are provided in Table 1. Its structure can be viewed as a quite distorted Au cube, having a missing vertex and lying within an (AlCp*)_6_ octahedron. The other energy minima exhibit more or less similar features. The hydride occupies a terminal position at a gold atom (Au–H = 1.664 Å). The corresponding computed Au–H stretching frequency (1745 cm^–1^) is close to the experimental value, as well as the computed ^13^C and ^1^H NMR chemical shifts (Figure S59). Two other low-energy isomers also meet these criteria (Figure S59). Unfortunately, neither the ^1^H chemical shift of the hydride of **2** (computed value: 8.8 ppm) nor the ^2^H of the deuteride **2D** could be experimentally recorded, likely due to fluxionality. As its isoelectronic relative [HCu_7_Al_6_](Cp*)_6_, the shape of cluster **2** is enough pseudospherical for being described as an 18-electron superatom^8,47^ of 1S^2^ 1P^6^ 1D^10^ configuration, with 1 and 2 electron(s) provided by each Au(0) and Al(I) atom, respectively, with the exception of the Au atom described as Au(I), linked to the terminal hydride (H^–^), which is formally a 0-electron supplier ligand. Consistently, the five highest Kohn–Sham orbitals are of large 6s(Au) and 3*s*/3p(Al) character and resemble, although somehow dented, the pure 1D orbitals of a quasi-spherical superatom (Figure S60).

**Table 1:** Selected Computed Data for the Lowest Energy Isomers of Compounds **1** and **2**. WBI = Wiberg Bond Index (in Brackets)

compound		1	2
**HOMO–LUMO gap (eV)**		1.96	1.40
**distances (Å) [WBI]**	Au–Au (av. and range)	2.898 [0.025] 2.762–2.995	2.910 [0.019] 2.792–3.073
	Al–Au (av. and range)	2.574 [0.322] 2.501–2.667	2.567 [0.316] 2.484–2.671
	Au–H		1.664 [0.436]
**NBO charges**	Au (av. and range)	0.00 (−0.02)–(+0.03)	+0.07 (−0.03)–(+0.24)
	Al (av. and range)	+0.57 (+0.50)–(+0.63)	+0.55 (+0.49)–(+0.71)
	H		–0.38

**Figure 1:**
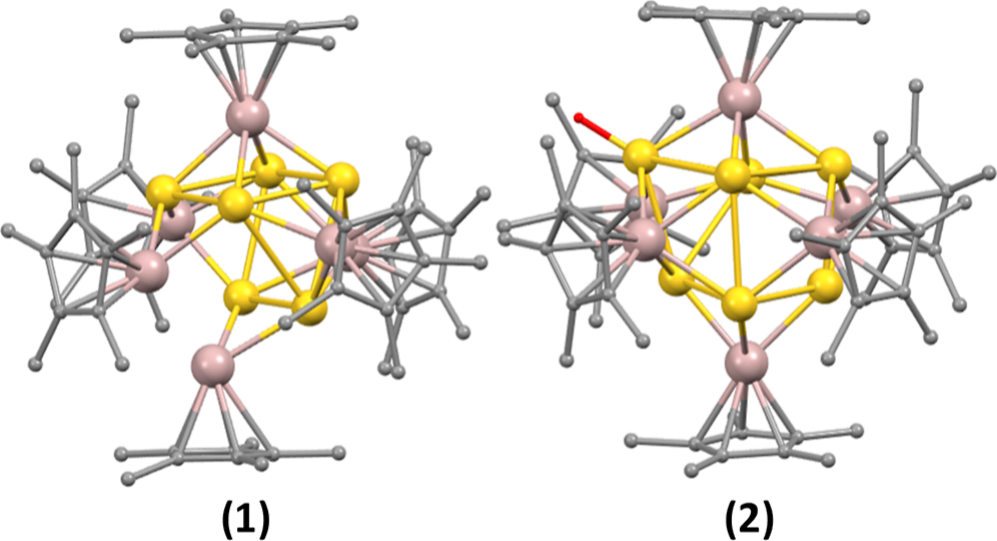
DFT-optimized geometries of the lowest energy isomers [Au_6_Al_6_](Cp*)_6_ (**1**) and [HAu_7_Al_6_](Cp*)_6_ (**2**). Color code: Au, yellow; Al, magenta; and C, gray. Hydride ligand of the AuH moiety is shown in red.

Three low-energy isomers (Figure S59) were found for **1**, of which the two lowest are almost degenerate in total energy and differ by less than 4 kcal/mol in free energy. They can all be viewed as deriving from a substantially distorted Au cube having two missing vertices and lying within an (also distorted) (AlCp*)_6_ octahedron. The isomer of lowest energy is shown in Figure 1 and selected computed data are provided in Table 1. It is also possible to view it as an 18-electron superatom with its five highest occupied orbitals constituting the 1D set (Figure S60).

Clusters **1** and **2** show similar bonding features (Table 1), with strong Au–Al bonding and weaker Au–Au interactions. The Au–H WBI indicates a substantial covalency. The Au and Al NBO charges reflect their actual oxidation states, and the rather moderate negative charge of the hydride is consistent with the Au–H covalency.

TD-DFT calculations on the lowest energy isomers of **1** and **2** were performed with the PBE0 hybrid functional and the Def2SVP basis set with the solvent effect (see Computational Details). The simulated spectra are in sufficient agreement with the experimental data (Figures S62 and S63).

In the case of **1**, the three bands of lowest energy have maxima at 521, 442, and 377 nm. The first one corresponds to a HOMO–1 → LUMO–1 transition, thus being of 1D → 1F nature. The second one (mainly HOMO → LUMO+3 and HOMO–2 → LUMO+2) and the third one (mainly HOMO–1 → LUMO+5, HOMO–1 → LUMO+6, and HOMO–2 → L+6) are also of a 1D → 1F nature. The intense band of higher energy peaking at 313 nm corresponds to π*(Cp*) → 1F, i.e., LMCT, transitions.

A comment about isomerism should be made at this point of the discussion. Although we found several low-energy minima in the cases of both **1** and **2**, we cannot certify that their numbers are not larger than those we found. In fact, all the computed isomers can be described as approximately spherical 18-electron superatoms, and it is likely that their shape is soft enough to allow the generation of several structures of the same type in the reaction medium, with possible interconversion between them. Such a feature, possibly leading to inseparable and cocrystallizing isomers, is consistent with the experimental structural data obtained by the XRD study of **2** (Figure S58), which can be described by a disorder model.

The parentage between the 18-electron superatoms [HAu_7_Al_6_](Cp*)_6_ and [HCu_7_Al_6_](Cp*)_6_^11^, both made of a (Au/Cu)_7_ core embedded within an (AlCp*)_6_ octahedron, suggests the possibility of the existence of [Au_8_Al_6_](Cp*)_6_, isoelectronic to the isolated [Cu_8_Al_6_](Cp*)_6_.^11^ The latter is a 20-electron species (1S^2^ 1P^6^ 1D^10^ 1S^2^ configuration), featuring a Cu_4_@Cu_4_ tetracapped tetrahedron (contracted cube) encapsulated within an (AlCp*)_6_ octahedron. Our search for a stable closed-shell [Au_8_Al_6_](Cp*)_6_ species ended up with a structure exhibiting a quite regular (not contracted) Au_8_ cube encapsulated within an (AlCp*)_6_ octahedron and for which the unique favored closed-shell electron count is 18, corresponding to the dication {[Au_8_Al_6_](Cp*)_6_}^2+^ (Figure S59d). The difference between the “Cu_8_Al_6_” and “Au_8_Al_6_” species is their different availabilities of the superatomic 2S orbital. It is the HOMO in the Cu/Al case, but only the LUMO+4 in the Au/Al case, with the LUMO to LUMO+3 being part of the 1F set. The nonavailability of the 2S orbital in the Au species is consistent with the lower capacity of its Au_8_ core. Calculations on the isoelectronic hypothetical dihydride [H_2_Au_8_Al_6_](Cp*)_6_] provided similar results as for its [Au_8_Al_6_](Cp*)_6_]^2+^ dicationic parent, thus suggesting the possibility for these two 18-electron species to be observed.

### Synthesis and Characterization of [Au_2_Al_5_](Cp*)_5_ (3)

**Scheme 3:**
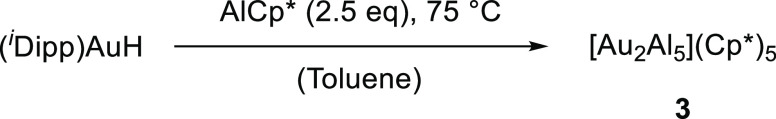
Synthesis of the [Au_2_Al_5_](Cp*)_5_ (**3**)

The reaction of ^*i*^DippAuH with excess of AlCp* (2.5 equiv) in toluene after prolonged reaction times (18 h) leads to the formation of the cluster [Au_2_Al_5_](Cp*)_5_ (**3**) (Scheme 3).

**Figure 2:**
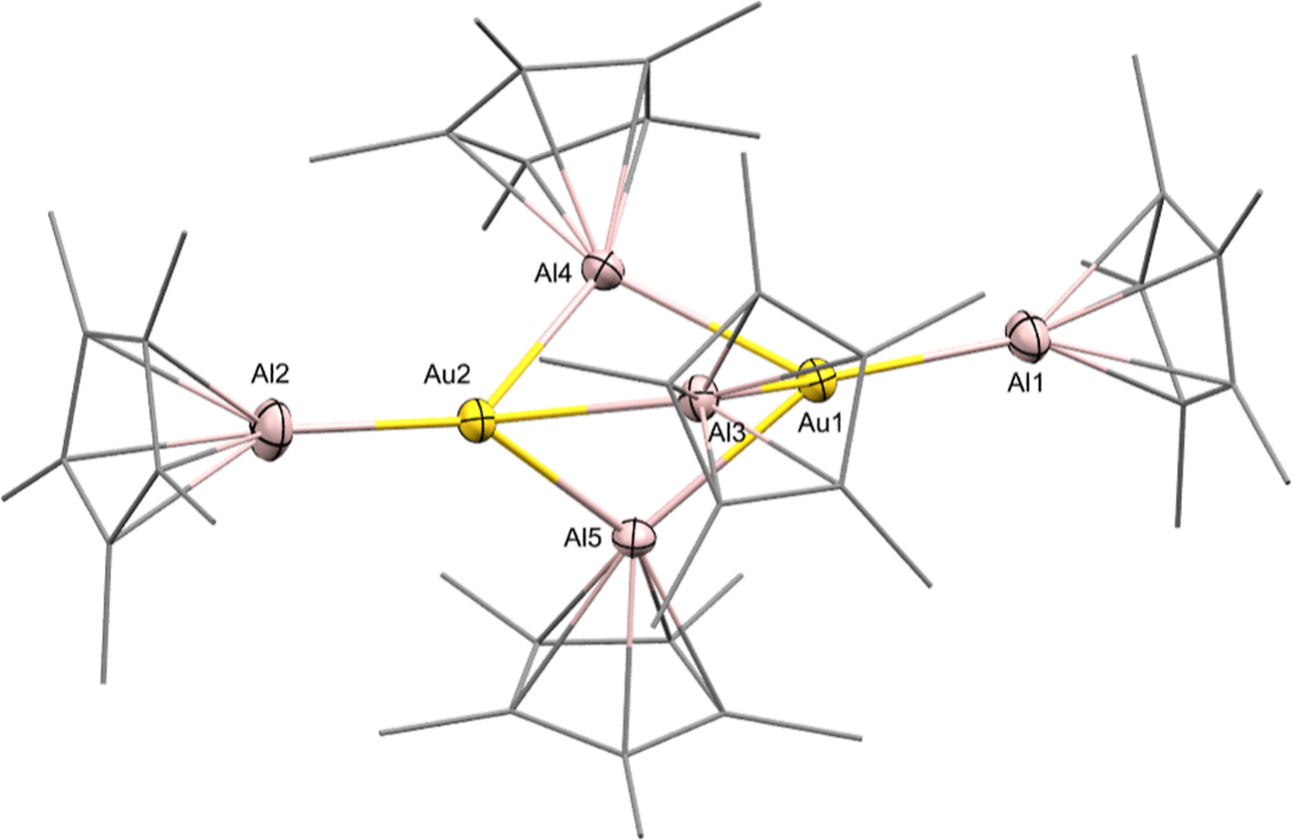
Molecular structure of [Au_2_Al_5_](Cp*)_5_. Au: yellow, Al: pink, and C: gray. H atoms and cocrystallized solvent molecules are omitted, and Cp* ligands drawn in wireframe representation for clarity. Thermal ellipsoids are shown at the 50% probability level.

The preparative purity of **3** is confirmed by ^1^H NMR analysis in benzene-d_6_, exhibiting two broad signals at 2.21 and 1.86 ppm with an integral ratio of 3:2, assigned to the bridging and terminal AlCp* ligands, respectively (see Figure S14). The ^13^C NMR spectrum of **3** in toluene-d_8_ (see Figure S15) shows the two signals of the -*C*H_3_ groups of the terminal and bridging AlCp* moieties at 13.65 and 10.23 ppm. However, only one signal at 114.19 ppm is observed for the quaternary carbon atoms. The elemental analysis yields satisfying data. LIFDI-MS analysis of the isolated product shows the molecular ion peak **3**-H^+^ as a weak signal at *m*/*z* = 1203.4 (see Figure S16) besides several fragment species of **3**, such as [AuAl_2_](Cp*)_2_^+^ (*m*/*z* = 521), [AuAl_3_](Cp*)_3_^+^(*m*/*z* = 683), and [HAuAl_4_](Cp*)_4_^+^ (*m*/*z* = 845). Obviously, **3** is quite unstable under the measurement conditions. The LIFDI-MS of isolated **3** shows the presence of trace impurities; these include the larger clusters **1** and **2**, as well as numerous [Au_*x*_(^*i*^Dipp)_*y*_]^+^ aggregates, whose trace impurities are much better ionizable than **3**. The IR spectrum of **3** (see Figure S17) shows the characteristic modes of the Cp* ligand as well as the Al-Cp* stretching frequency at 455 cm^–1^. There is no signal of surface bound hydride observed in the spectrum. The UV–vis spectrum of isolated **3** (see Figure S18) consists of a broad, weak band at 516 nm, a band centered at 417 nm, as well as a broad and intense band at 406 nm. In the UV region, a sharp band at 331 nm is detected. A concentrated solution of **3** in toluene has a green-brown color and turns yellow upon dilution. Cluster **3** forms black block-shaped crystallites, which are very air- and moisture-sensitive. It crystallizes in the monoclinic space group *P*21*/n* with four cluster molecules per unit cell and four molecules of cocrystallized toluene (Figure 2). Detailed crystallographic information is listed in Table S1. The structure of **3** consists of a digold-centered trigonal-bipyramidal structure with idealized *D*_*3h*_ symmetry. The central axis of **3** slightly deviates from linearity (Al2–Au2–Au1 = 175.1° and Al1–Au1–Au2:174.6°). The central Au_2_ unit (Au1–Au2 = 3.881 Å) is surrounded by two terminal AlCp* units and three bridging AlCp* units with obtuse angles between 98.9° (Au2–Al5–Au1) and 99.5° (Au2–Al3–Au1), spanning an almost equilateral triangle with Al–Al–Al angles ranging from 67.5 to 68.6°. Au-AlCp*_terminal_ = 2.397–2.420 Å and Au-AlCp*_bridging_ = 2.537–2.585 Å. The Au2–Al2 bond in **3** is 2.397 Å, the shortest molecular Au–Al bond reported so far and well within the sum of the covalent radii of the two elements (2.57 Å).^48^ The structure of **3** is comparable with the literature known compounds [Pd_2_(AlCp*)_5_], [M_2_(GaCp*)_5_] (M = Pd, Pt), and [Ni_2_(AlCp*)_5_], but deviation from linearity is more pronounced.^49,50^ The Au–Al distances in **3** (Figure S55) are well comparable to those in the trimetallic cluster [Ni(AuPPh_3_)_6_(AuCl)_3_(AlCp*)] [2.596(5) Å – 2.633(6) Å]^19^ and to the strongly polarized Au^δ−^–Al^δ+^ bond [2.402(3) Å] in the complex [(NON)Al(AuP^*t*^Bu_3_)].^51^ Overall, Au–Al bond distances in **3** are slightly shorter than in the solid-state alloy AuAl_2_ (2.58 Å) as one reference structure.^52^ Notably, the Au–Au distance in **3** (Au1–Au2 = 3.881 Å) is much longer than in related dimeric molecules or small clusters unambiguously featuring direct, covalent Au–Au bonding.^19,53,54^ The interesting bonding situation and electronic relationship to Ni_2_(CO)_5_ and Ni_2_(AlCp*)_5_ are discussed in a separate publication.^55^

The interconnection between larger clusters **1** and **2** and smaller cluster **3** was studied with in situ ^1^H NMR. The clusters **1** and **2** degrade upon heating in the presence of AlCp* and cluster **3** is formed (see Figures S23–S25). Treatment of isolated **3** with ^*i*^DippAuH (2 equiv) at room temperature results in complete consumption of **3** already after 2.5 h reaction time and the formation of [Au_6_Al_6_](Cp*)_6_ (**1**) (Figure S27).

## Conclusions

Three novel members of intermetalloid Au/Al clusters are prepared and characterized within the scope of this work. The geometric and electronic structures of the larger clusters [Au_6_Al_6_](Cp*)_6_ (**1**) and [HAu_7_Al_6_](Cp*)_6_ (**2**) were investigated through DFT calculations; both clusters can be described as 18-electron superatoms. While SC-XRD of **1** and **2** leads to unsatisfactory results, possibly due to cocrystallizing of cluster isomers, the smaller cluster [Au_2_Al_5_](Cp*)_5_**(3**) is well characterized by SC-XRD.

The synthesis of the described gold clusters can be controlled using various phosphines as additives. The presence of PPh_3_ changes the reduction mechanism of ^*i*^DippAuH, which is a key step in cluster formation: with an increasing amount of PPh_3_, the reduction by AlCp* is disfavored, and the reductive elimination of H_2_ becomes dominant. This has a remarkable impact on the reaction outcome and the formation of **2** becomes selective.

## Supporting Information Available

Experimental details, spectroscopic data (NMR, IR, and UV–vis), mass spectra, crystallographic information, details to DFT-calculations (PDF)
