# Supplementary material for: All-Hydrocarbon-Ligated Superatomic Gold/Aluminum Clusters
Source: Inorg Chem. 2024 Feb 9;63(8):3749–56. doi: 10.1021/acs.inorgchem.3c03790 (PMC10900290; doi:10.1021/acs.inorgchem.3c03790)
Supplement: Supplementary file 1 — ic3c03790_si_001.pdf [file ic3c03790_si_001.pdf]

Supporting Information

All-Hydrocarbon Ligated Superatomic Gold/Aluminium Clusters.

Ivan Antsiburov[‡ a,b], *Max Schütz*[‡ a,b], *Raphael Bühler*[a,b], *Maximilian Muhr*[a,b], *Johannes Stephan*[a,b], *Christian Gemel*[a,b], *Wilhelm Klein*[a,b], *Samia Kahlal*[c], *Jean-Yves Saillard*[*c], and *Roland A. Fischer*[*a,b]

[a] Technical University of Munich, Department of Chemistry and Catalysis Research Center, Chair of Inorganic and Metal-Organic Chemistry, Lichtenbergstr. 4, 85748 Garching, Germany. E-mail: roland.fischer@tum.de

[b] Univ Rennes, CNRS, ISCR-UMR 6226, F-35000 Rennes, France. E-mail: jean-yves.saillard@univ-rennes1.fr

[‡] *Ivan Antsiburov* and *Max Schütz* contributed equally.

1. Materials and Methods 3
2. Characterization of isolated gold Clusters 4
3. Mechanistic investigations 15
3.1 Size-focusing of cluster libraries depending on Au:Al ratio 15
3.2 Cluster growth and degradation reactions 20
3.3 Reduction to Au(0) in the course of the cluster generating reactions 22
3.4 Influence of PR_3 on the product distribution 23
4. Synthesis of starting materials 33
5. Crystallography 40
6. DFT-Calculations 46
7. References 49

1. Materials and Methods

General

Unless stated otherwise, all synthetic manipulations were carried out using standard Schlenk techniques under an atmosphere of argon 4.6 purified by BTC-catalyst and dried over 3 Å molecular sieves or in a glovebox under an atmosphere of purified argon. All reactions were carried out in standardized *Schlenk* flasks and tubes which were rinsed with 1,1,1,3,3,3-hexamethyldisilyzane (HMDS), heated with a heat gun to 650 °C and cooled under vacuum argon. All synthesis of light sensitive compounds were performed under the red light. All solvents were carefully dried over molecular sieves and deuterated solvents were degassed prior to their use. All non-dried solvents used were distilled prior to their use. All the reagents were purchased from commercial sources and used as such without further purification.

Analytical Methods

NMR spectra were recorded on a Bruker Avance III 400US (^{1}H, 400 MHz; ^{13}C 101MHz), Bruker Avance III 300 (^{1}H, 300 MHz) and a Bruker Avance II 500 with a cryo-probe (^{1}H, 500 MHz; ^{13}C, 125 MHz). Chemical shifts are given relative to TMS and were referenced to the residual solvent peak as internal standards. Chemical shifts are reported in parts per million, downfield shifted from TMS, and are consecutively reported as position (δH or δC), relative integral, multiplicity (s = singlet, d = doublet, q = quartet, sept = septet and m = multiplet) and assignment. Variable temperature (VT) NMR spectra were recorded on a Bruker Avance 400 spectrometer (^{1}H, 400 MHz). FT-IR spectra were measured on an ATR setup with a Bruker Alpha FTIR spectrometer under an inert gas atmosphere in a glove-box. The mass spectra were taken using a Linden CMS LIFDI as ionization source and a ThermoFisher Scientific Exactive Plus Orbitrap as detector. The sample application was performed via a fumed silica capillary from a glovebox under an argon atmosphere to enable the measurement of highly air-sensitive compounds. The recorded mass spectra were evaluated using the FreeStyle 1.3 program from ThermoFisher Scientific and a fitting program developed by *Dr. Christian Gemel*.

2. Characterization of isolated gold Clusters

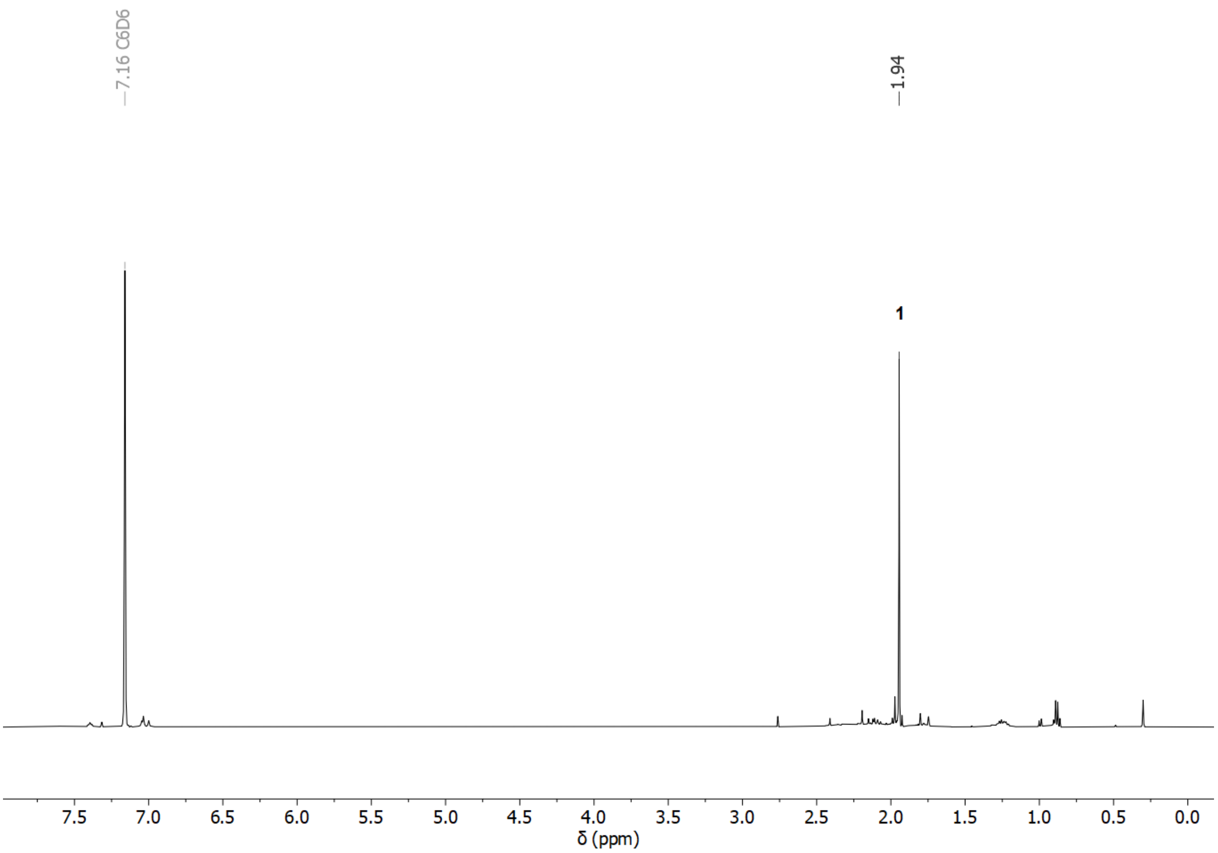

Figure S1: ^{1}H-NMR (benzene-d$_6$) spectrum of isolated **1**.

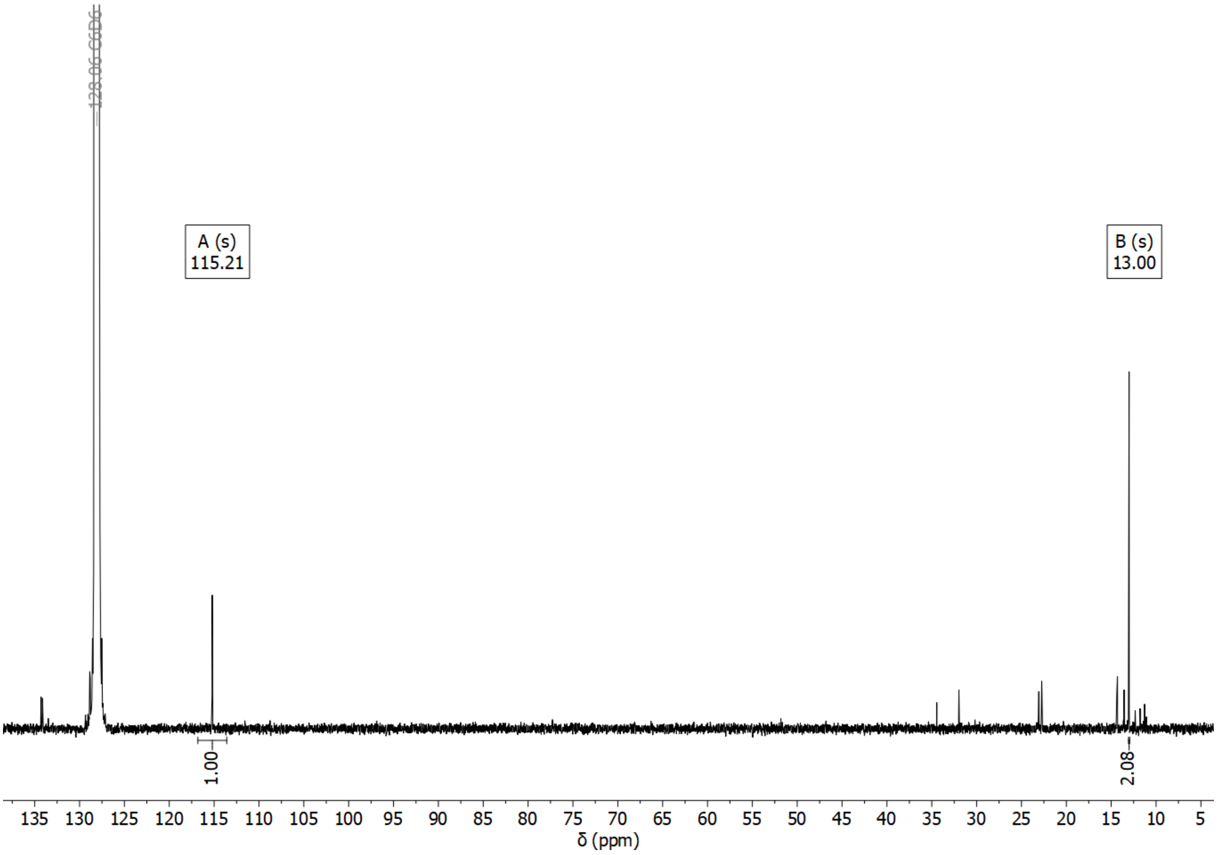

Figure S2: ^{13}C-NMR (benzene-d$_6$) spectrum of isolated **1**.

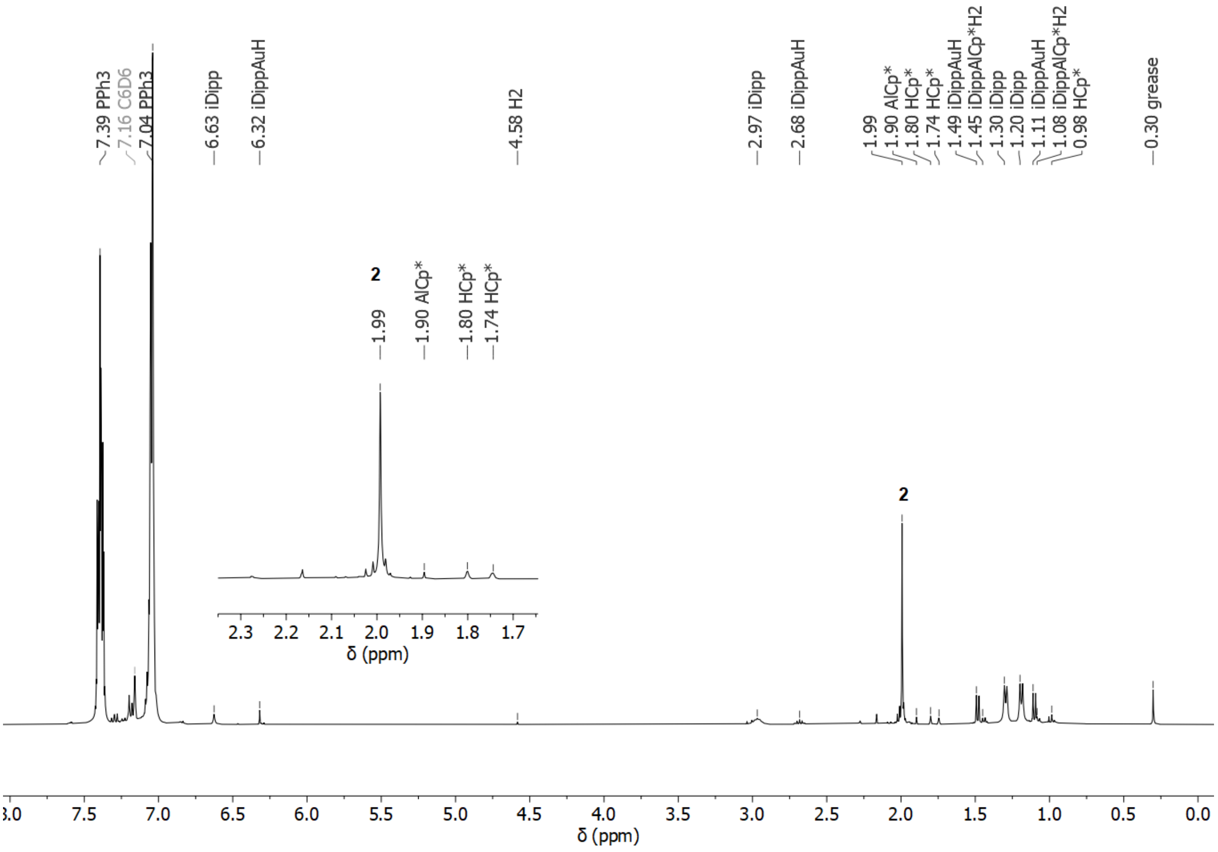

Figure S3: *In situ* ^{1}H-NMR spectra (benzene-d$_6$) of the reaction [^{i}DippAuH] + AlCp* + 10 PPh$_3$ (1:1, 75 °C, 2 h). The selective formation of **2** is obtained.

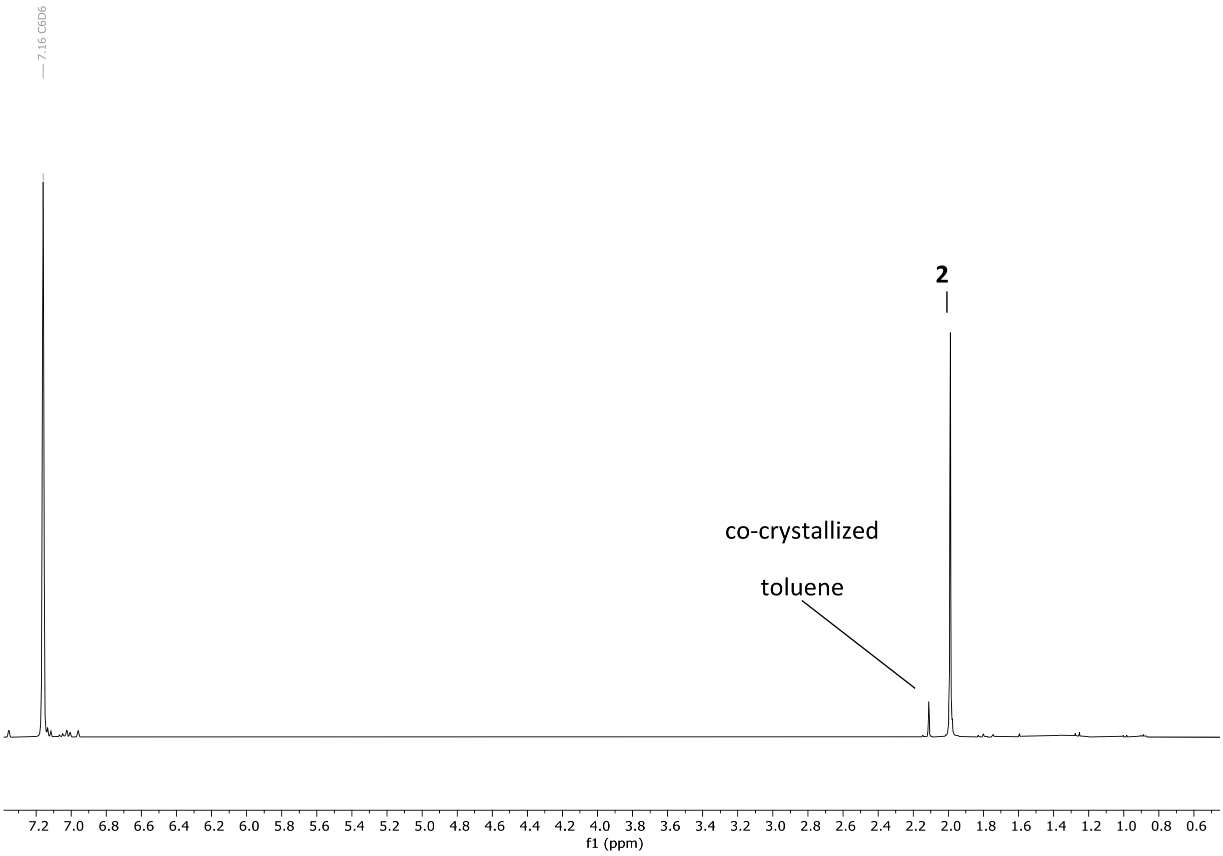

Figure S4: ^{1}H-NMR (benzene-d$_6$) spectrum of isolated **2**.

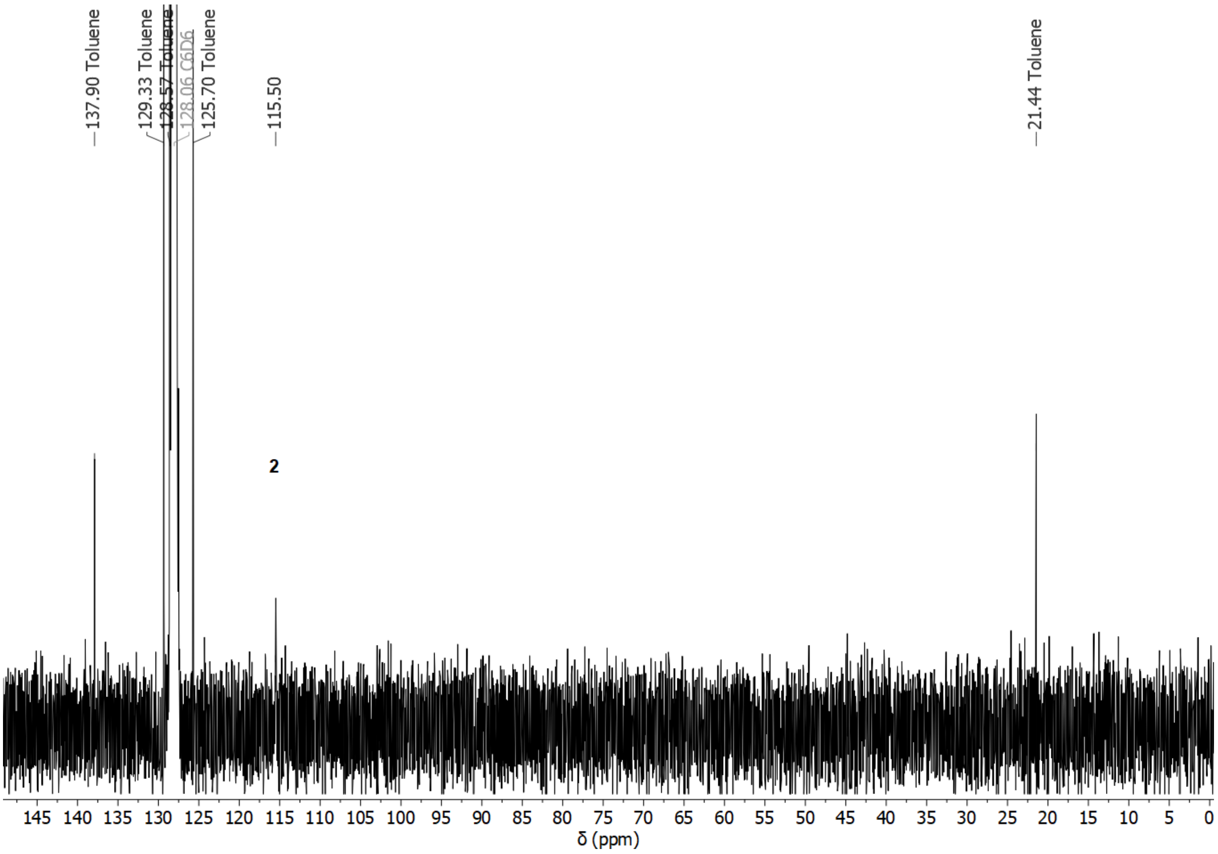

Figure S5: ^{13}C-NMR (benzene-d$_6$) spectrum of isolated **2**.

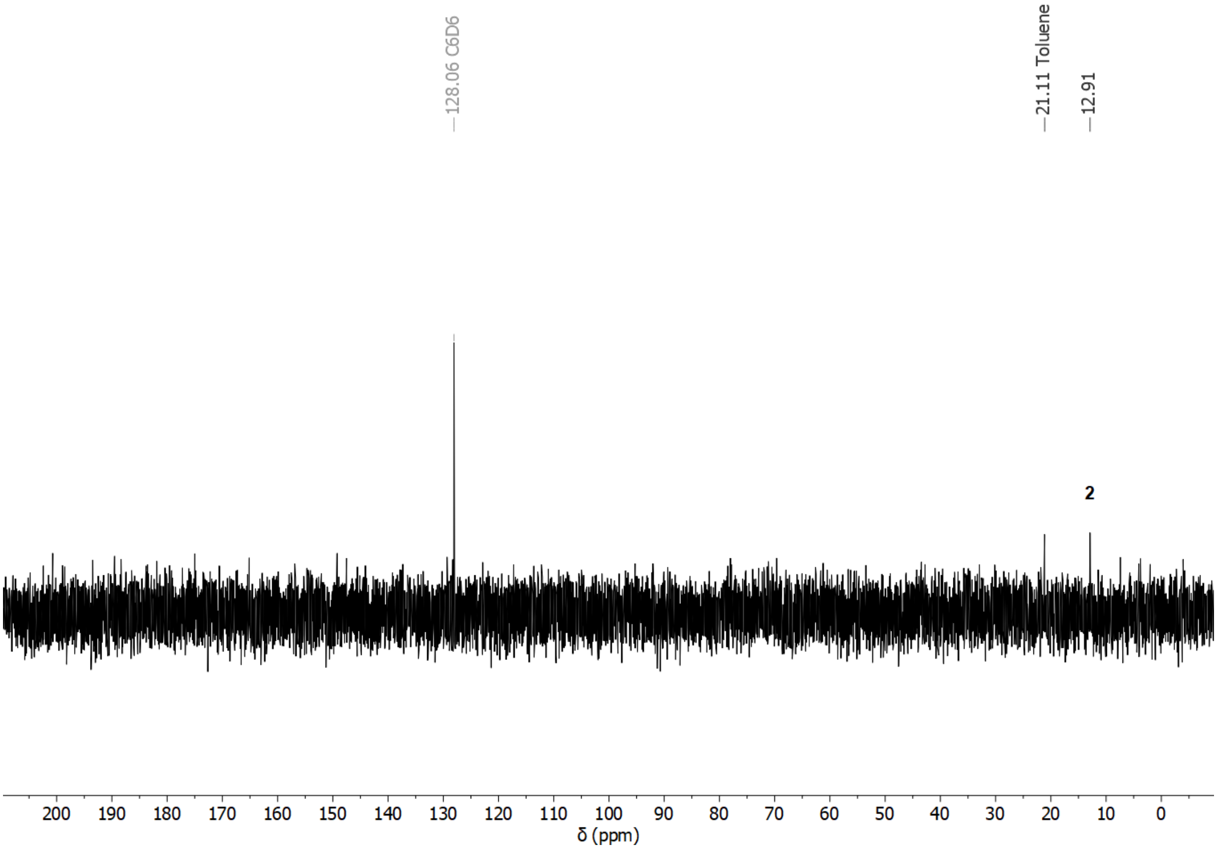

Figure S6: DEPT 135° spectrum (benzene-d_6) of isolated **2**.

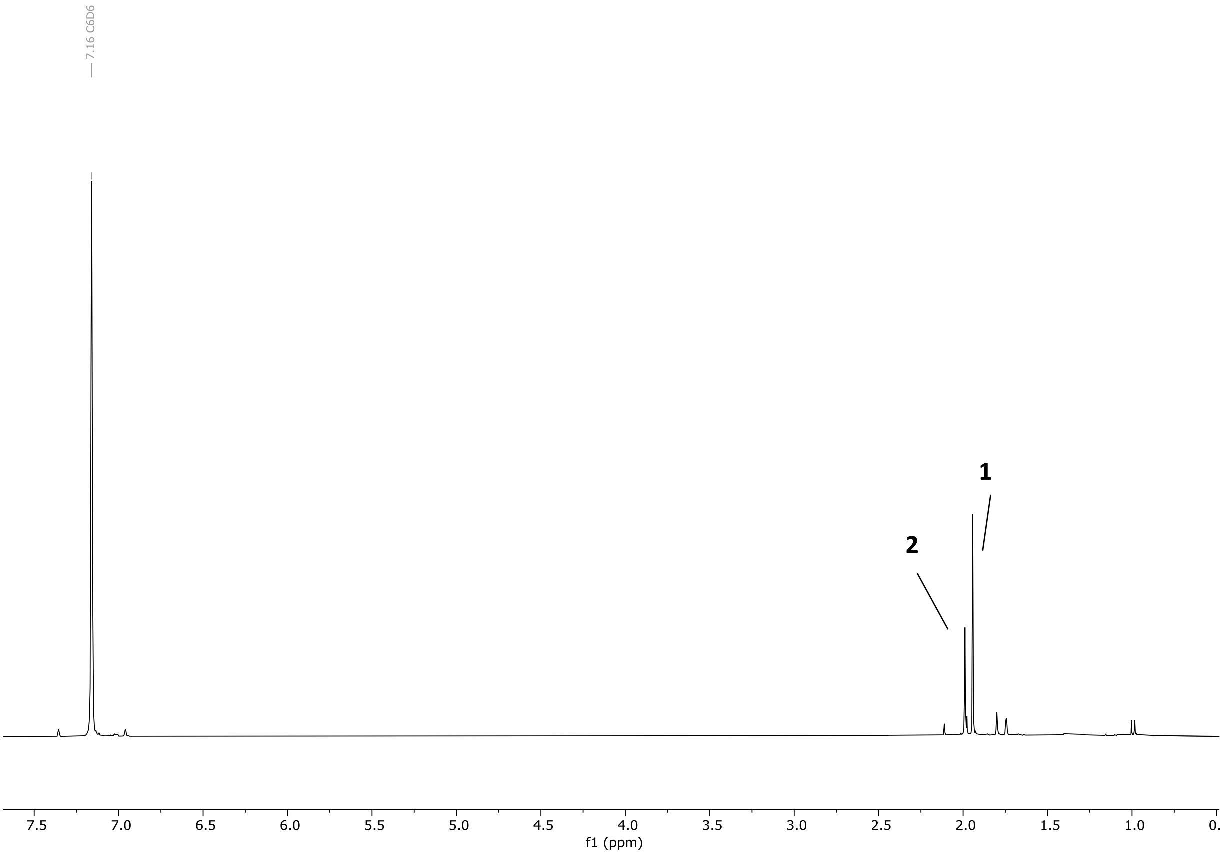

Figure S7: ^{1}H-NMR (benzene-d_6) spectrum of isolated **1/2.**

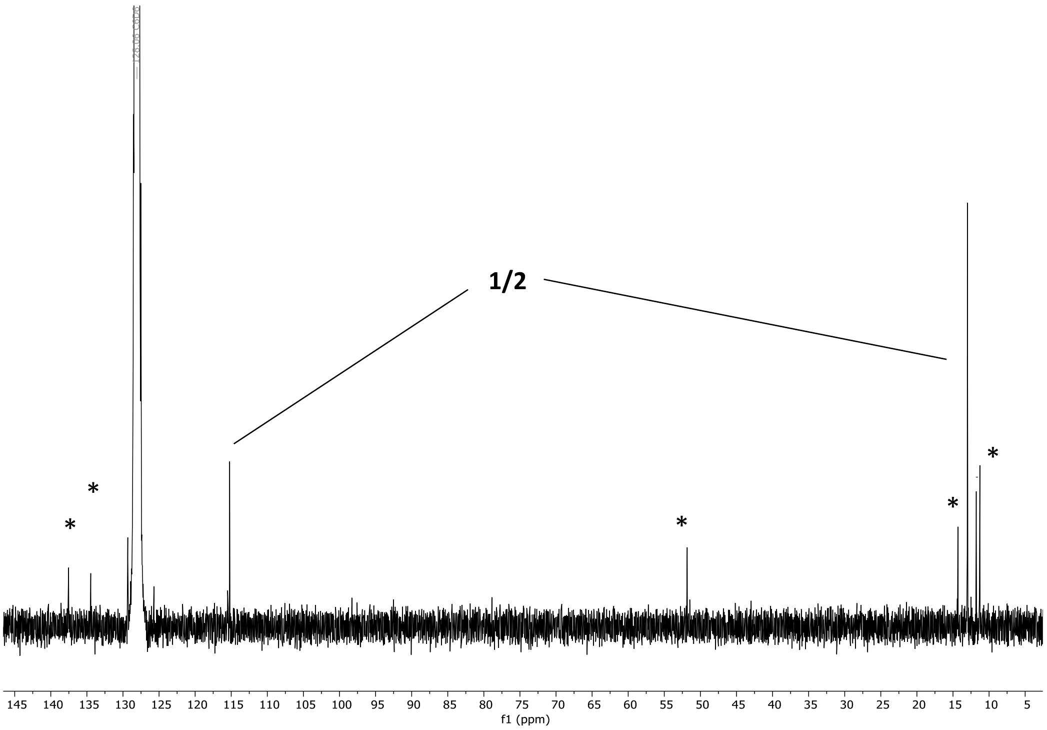

Figure S8: ^{13}C-NMR spectrum (benzene-d$_6$) of isolated **1/2**. Signals marked with * are attributed to free pentamethylcyclopentadiene (HCp*).

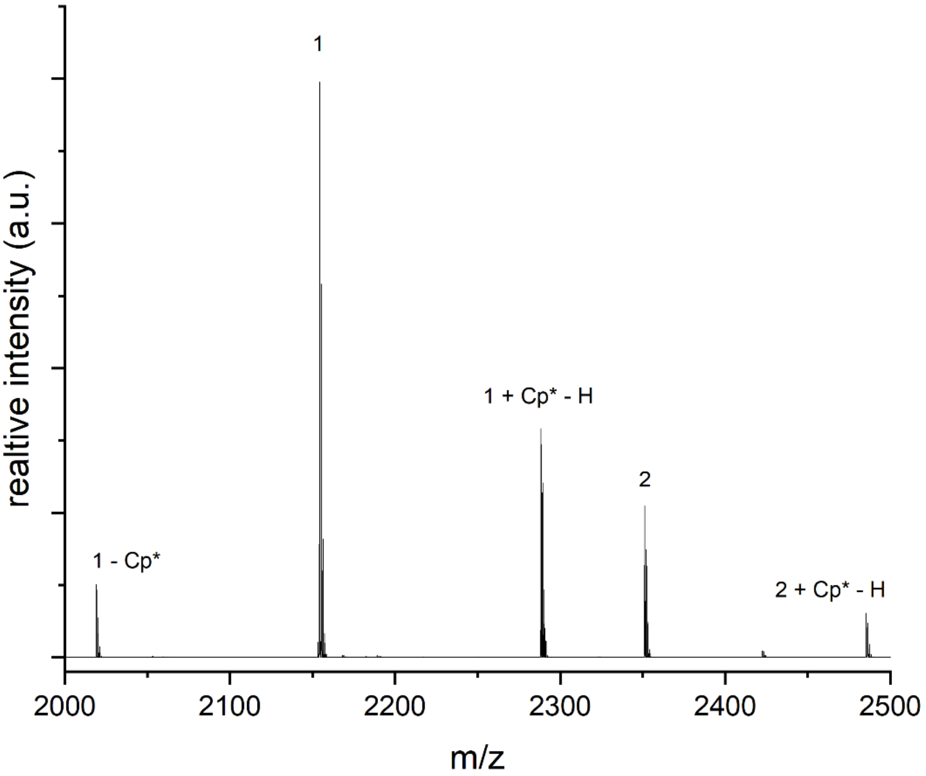

Figure S9: LIFDI-MS spectrum of isolated **1**.

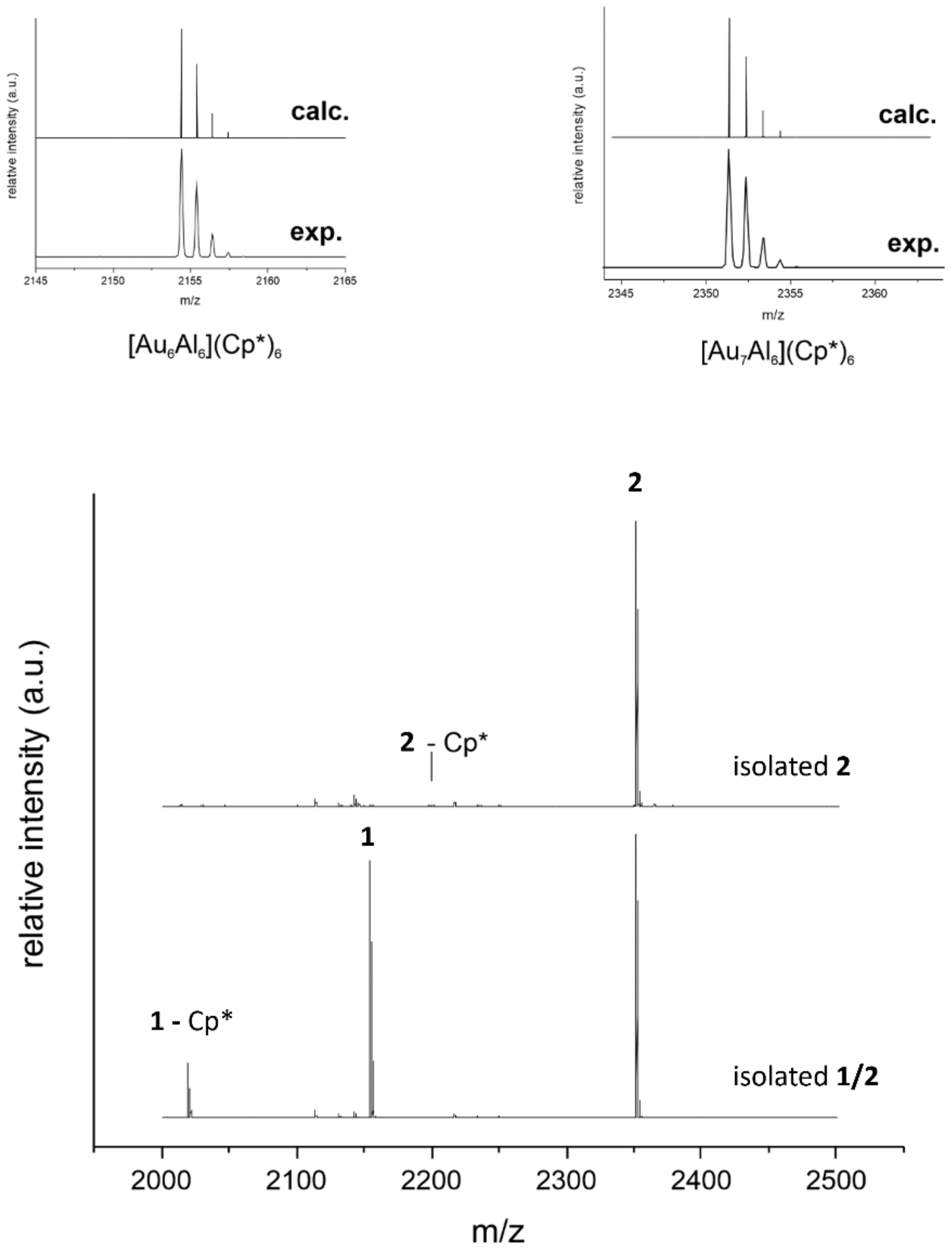

Figure S10: Bottom: Comparison of LIFDI-MS spectra of isolated **1/2** and pure **2**. Top: Comparison of calculated and experimental patterns of $[Au_6Al_6](Cp^*)_6$ and $[Au_7Al_6](Cp^*)_6$.

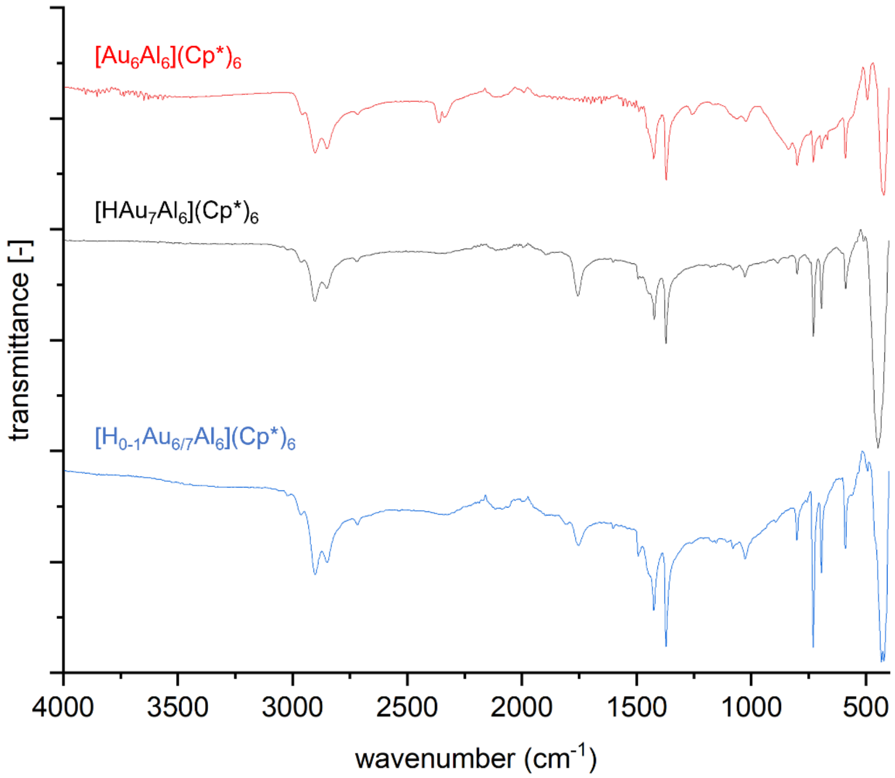

Figure S11: Comparison of ATR-IR spectra of $[Au_6Al_6](Cp^*)_6$ **(1)**, $[HAu_7Al_6](Cp^*)_6$ **(2)** and $[H_{0\text{-}1}Au_{6/7}Al_6](Cp^*)_6$ **(1/2).** The characteristic Au-H band in **2** and **1/2** can be seen at 1753 and 1756 cm^{-1} respectively.

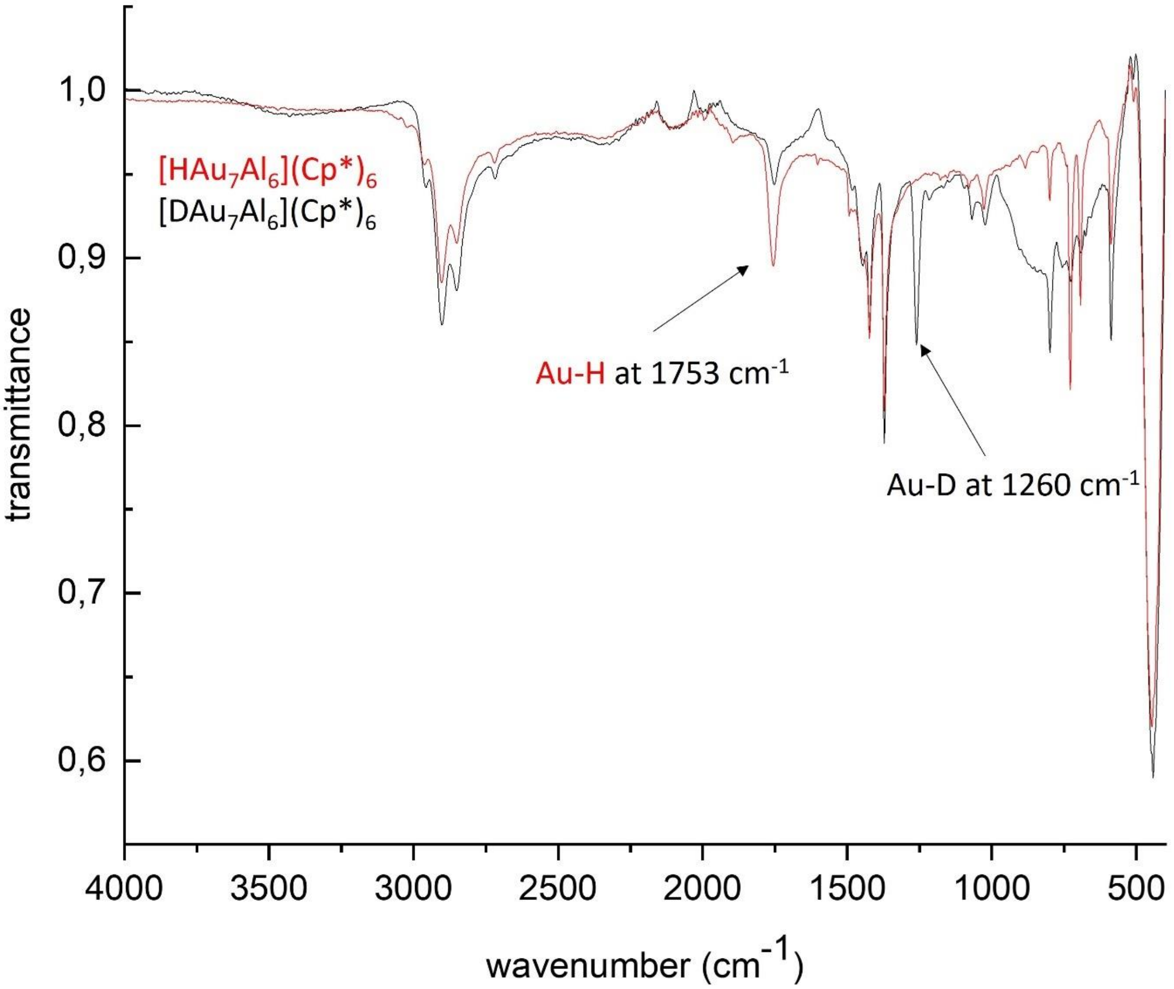

Figure S12: Comparison of ATR-IR spectra of [Hau$_7$Al$_6$](Cp*)$_6$ **(2)** and [DAu$_7$Al$_6$](Cp*)$_6$ **(2D)**. The characteristic Au-H and Au-D bands can be seen at 1753 and 1260 cm^{-1} respectively.

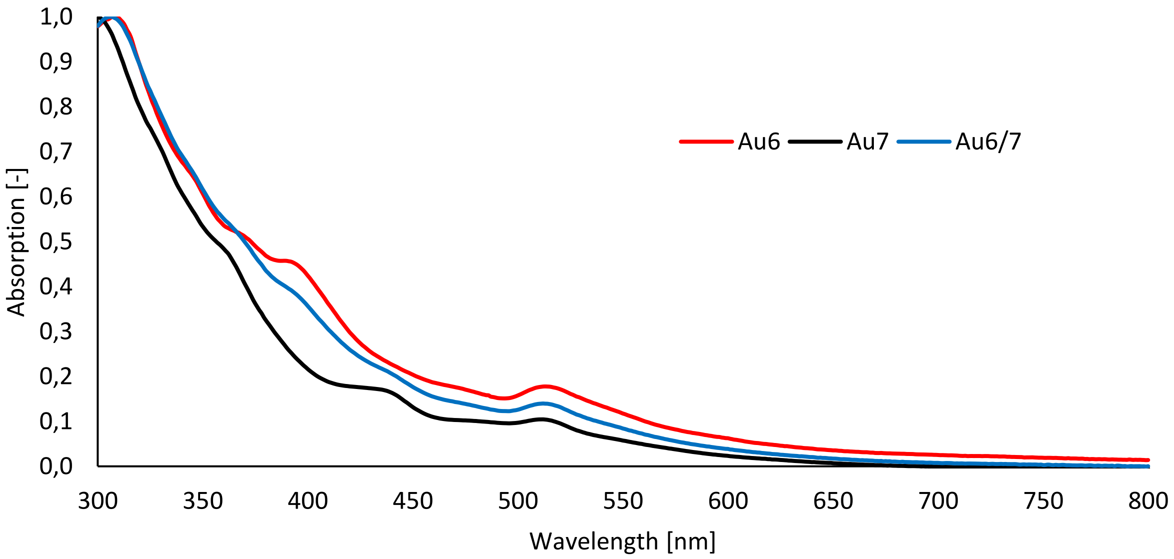

Figure S13: Comparison of UV-Vis spectra of isolated **1** (red), **2** (black) and **1**/**2** (blue).

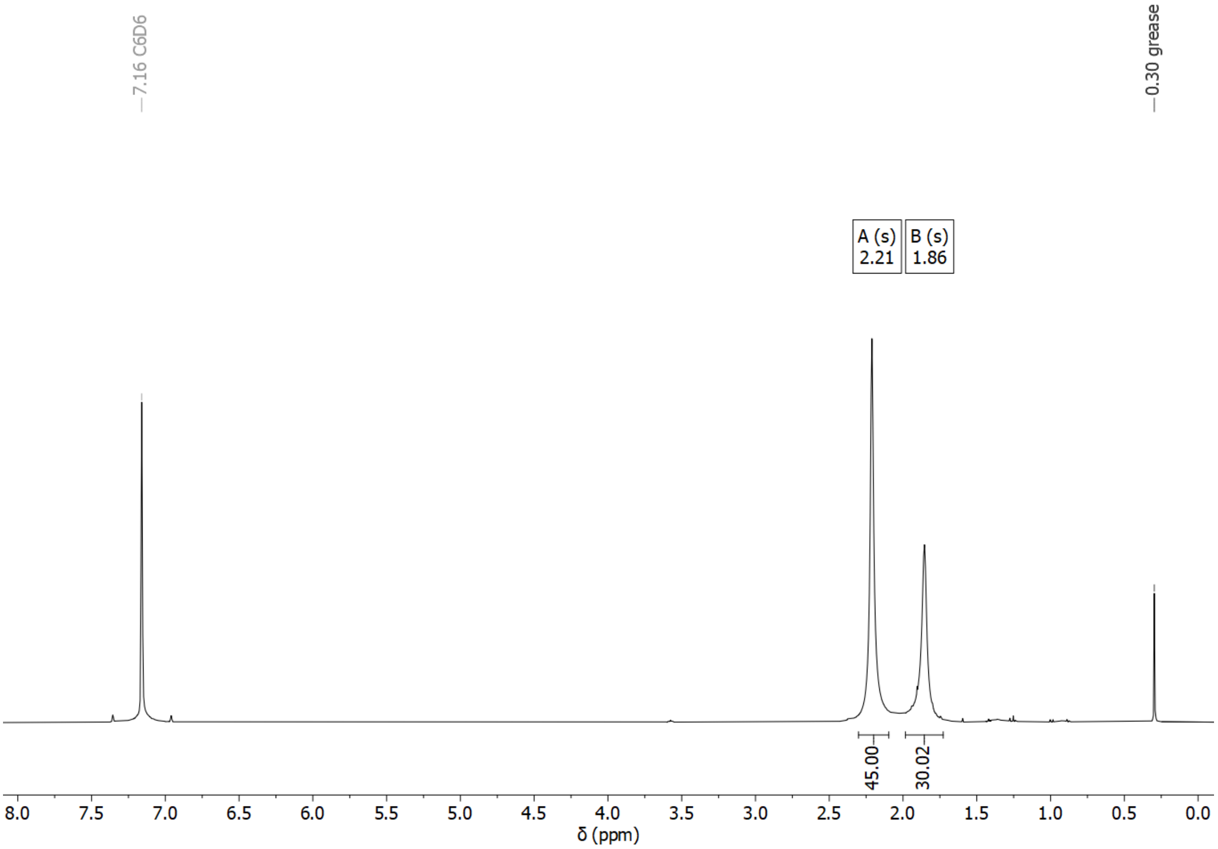

Figure S14: ^{1}H-NMR (benzene-d_6) spectrum of isolated **3**.

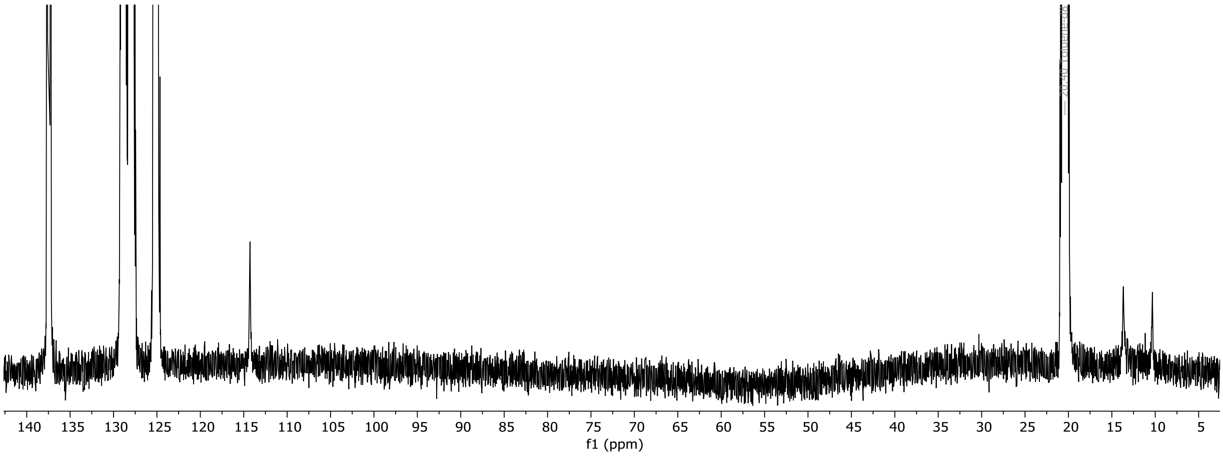

Figure S15: ^{13}C-NMR spectrum (toluene-d_8) of $[Au_2Al_5](Cp^*)_5$ (**3**). Peaks at 21 ppm, 125 ppm, 128 ppm and 137.5 ppm are solvent residual signal.

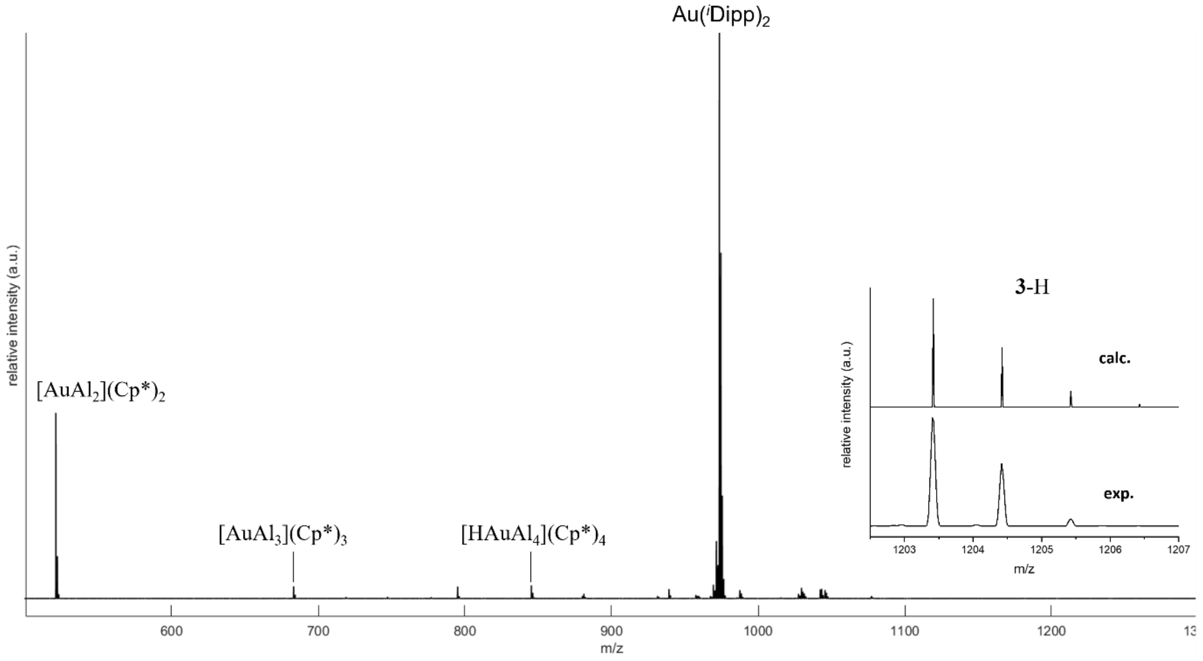

Figure S16: Relevant part of the LIFDI-MS spectrum of $[Au_2Al_5](Cp^*)_5$ (**3**).

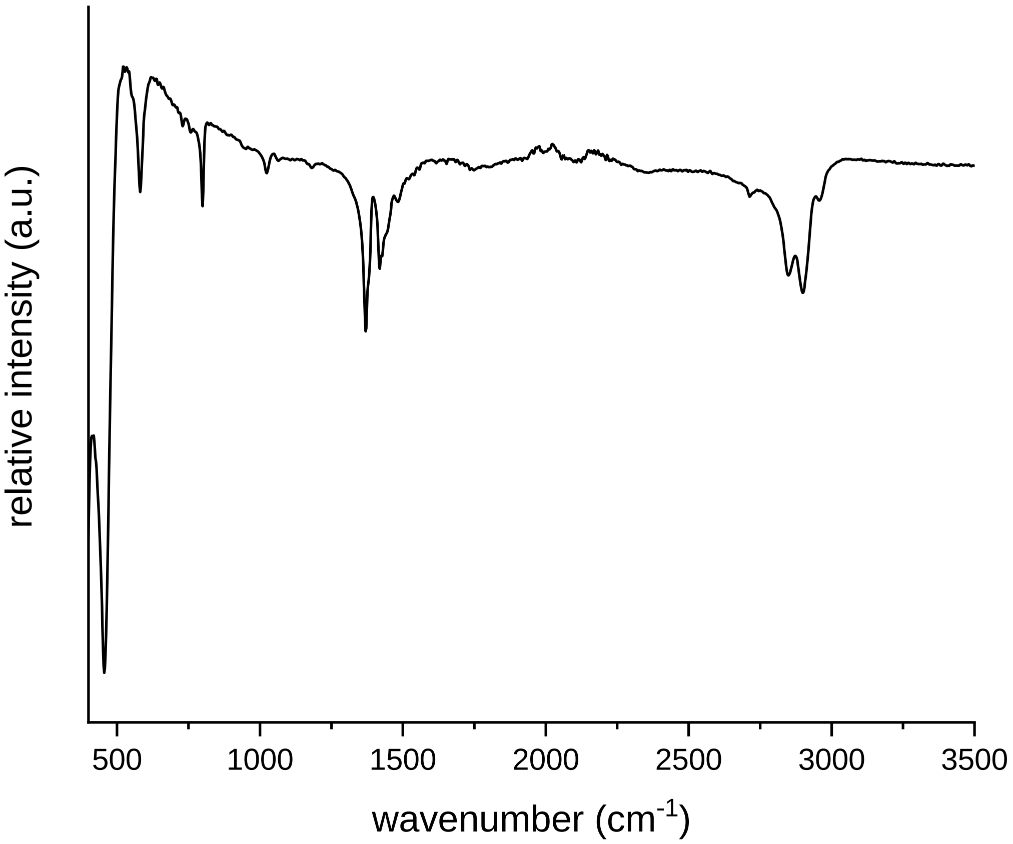

Figure S17: ATR-IR spectrum of $[Au_2Al_5](Cp^*)_5$ (**3**).

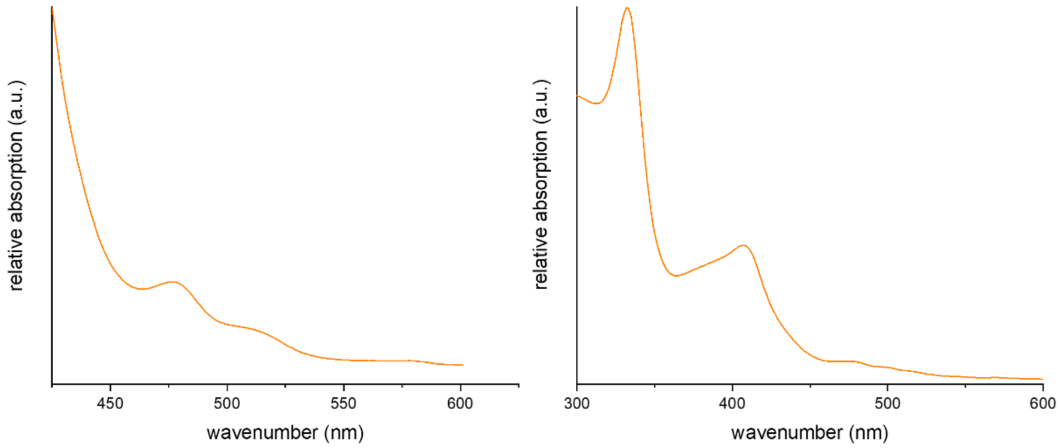

Figure S18: UV-Vis spectrum of isolated $[Au_2Al_5](Cp^*)_5$ (**8**). Left: Enlarged Vis region. Right: Full-range spectrum.

3. Mechanistic investigations

3.1 Size-focusing of cluster libraries depending on Au:Al ratio

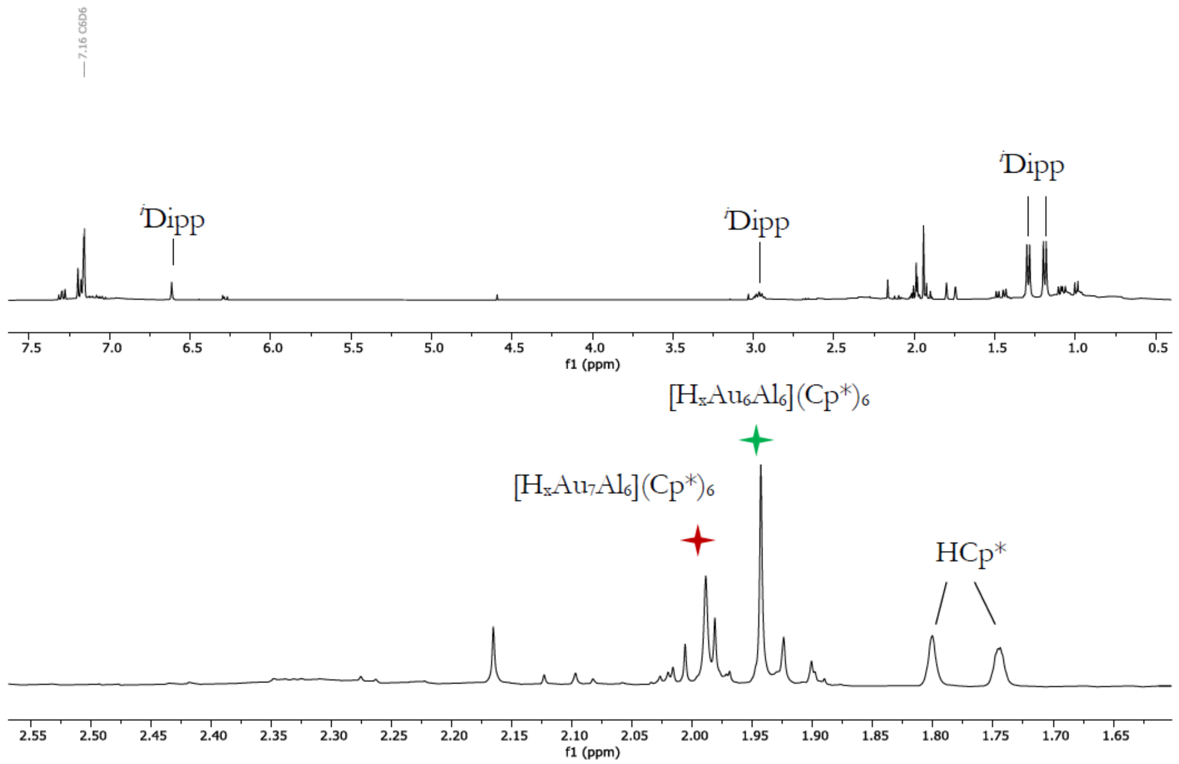

Figure S19: *In situ* ^{1}H-NMR spectra (benzene-d$_6$) of the reaction [^{i}DippAuH] + AlCp* (1:0.75, 75 °C, 2 h). The reaction is unselective and leads to the formation of cluster mixture **1/2**.

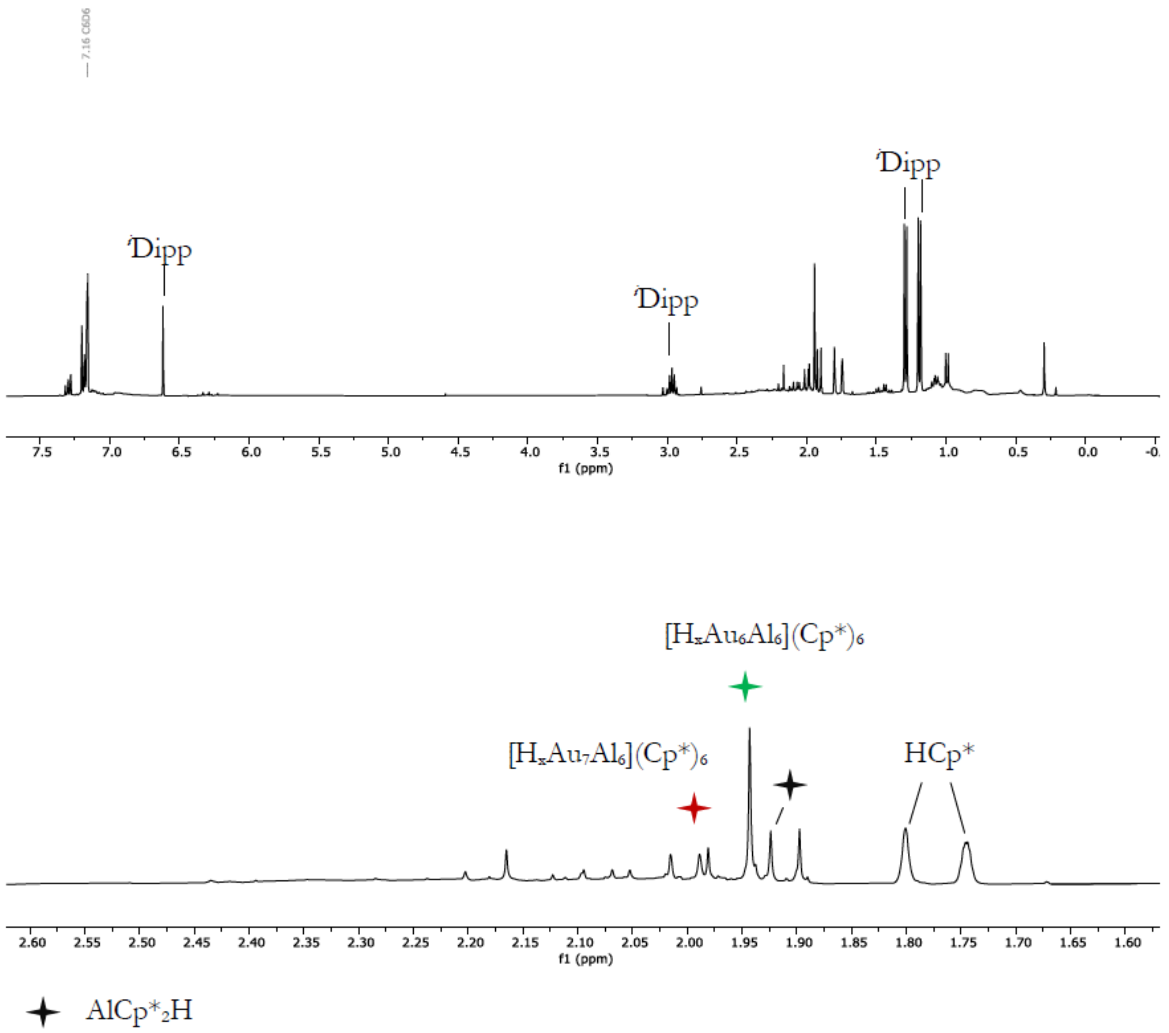

Figure S20: *In situ* ^{1}H-NMR spectra (benzene-d$_6$) of the reaction [^{i}DippAuH] + AlCp* (1:1.25, 75 °C, 2 h). Increased amount of AlCp* enhances the formation of **1**.

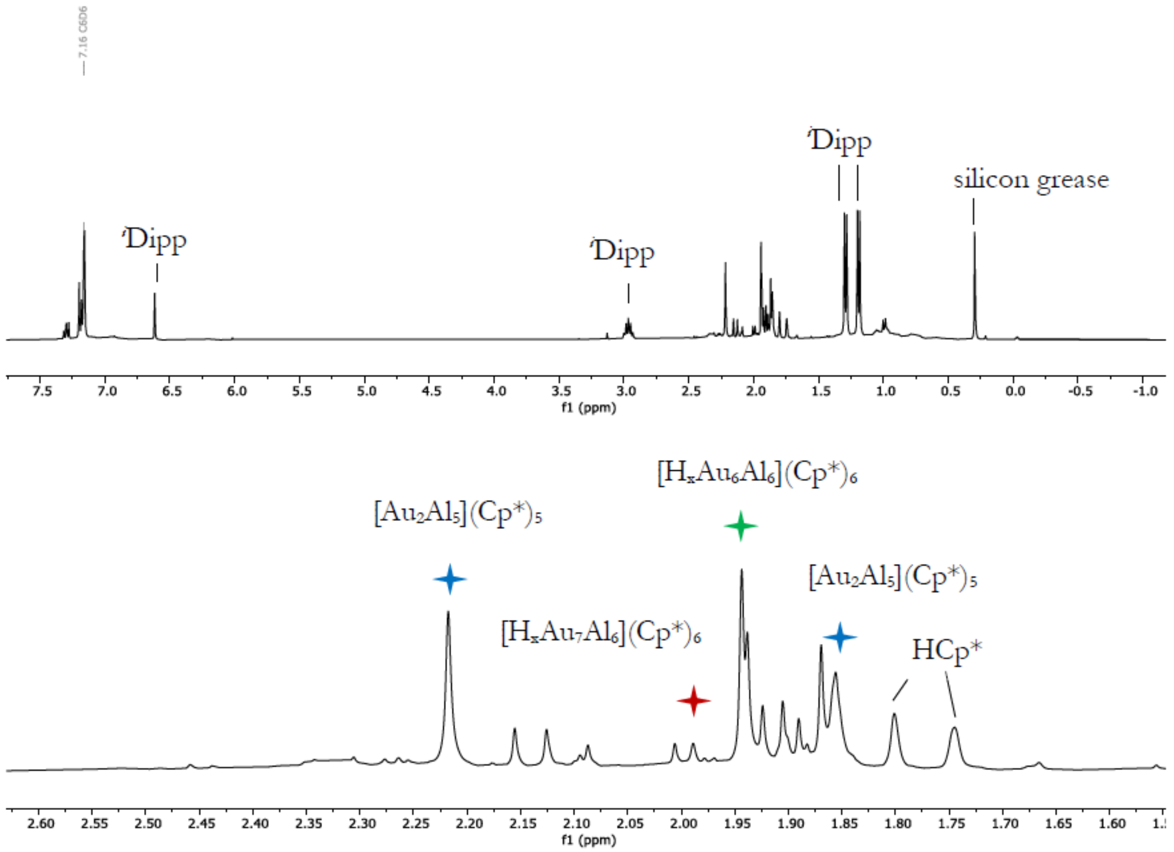

Figure S21: *In situ* ^{1}H-NMR spectra (benzene-d$_6$) of the reaction [^{i}DippAuH] + AlCp* (1:1.5, 75 °C, 2 h). Excess of AlCp* leads to the formation of **3**.

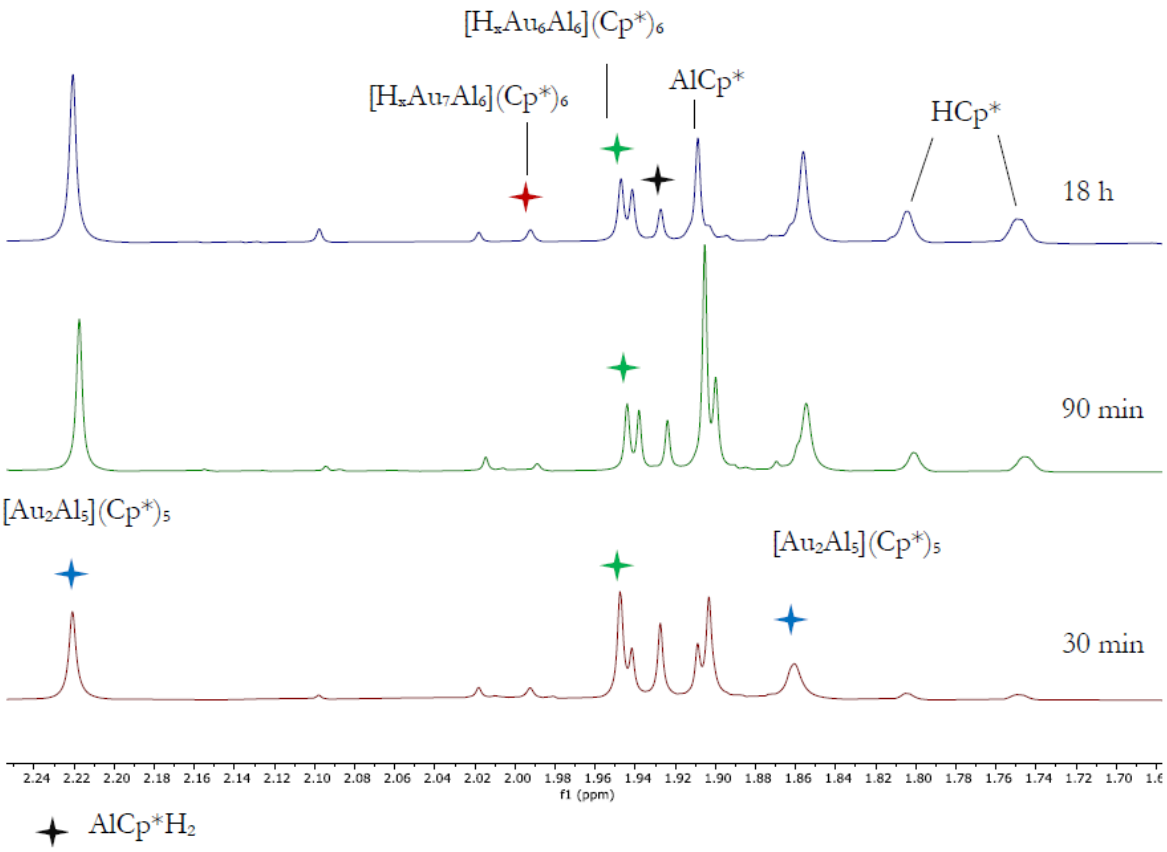

Figure S22: Time-dependent *in situ* ^{1}H-NMR spectra (benzene-d$_6$) of the reaction [^{i}DippAuH] + AlCp* (1:2, 75 °C).

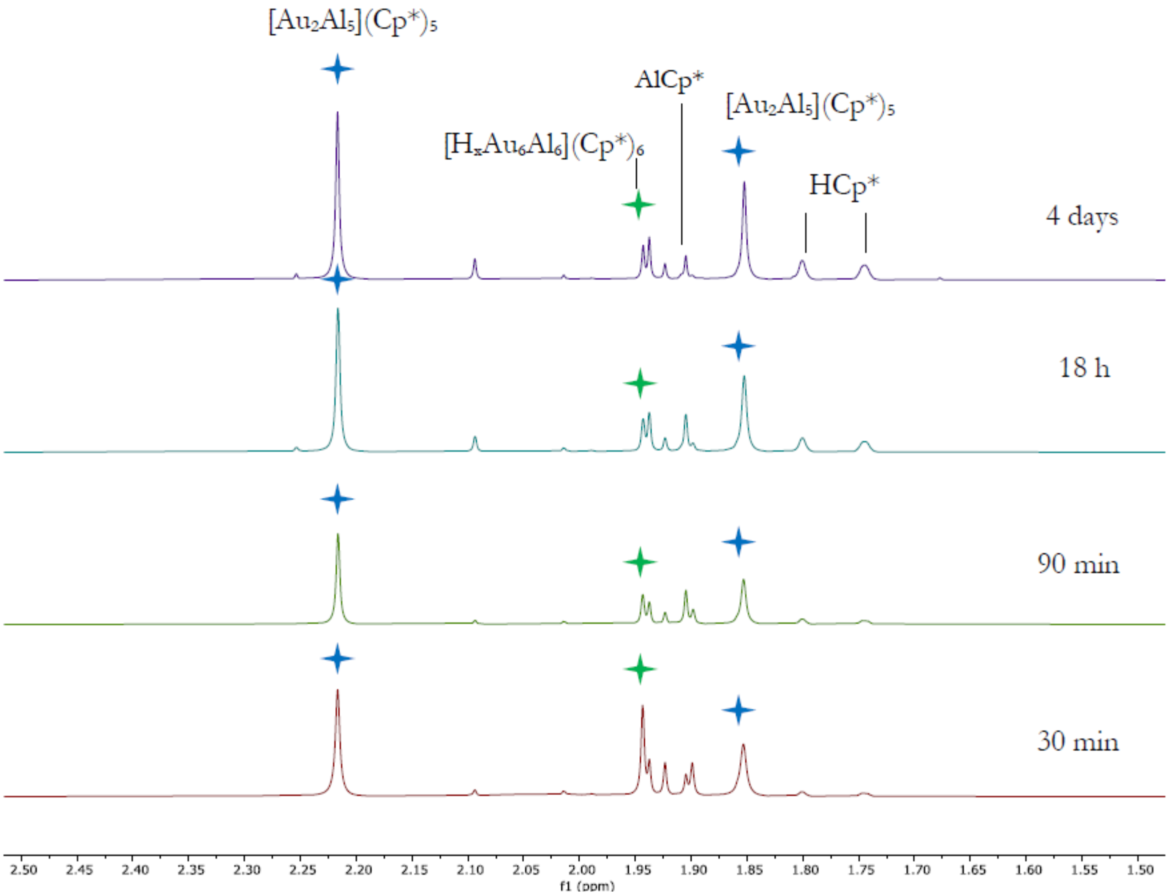

Figure S23: Time-dependent *in situ* ^{1}H-NMR spectra (benzene-d$_6$) of the reaction [^{i}DippAuH] + AlCp* (1:2.5, 75 °C). Prolonged heating is necessary for the high yield of **3**.

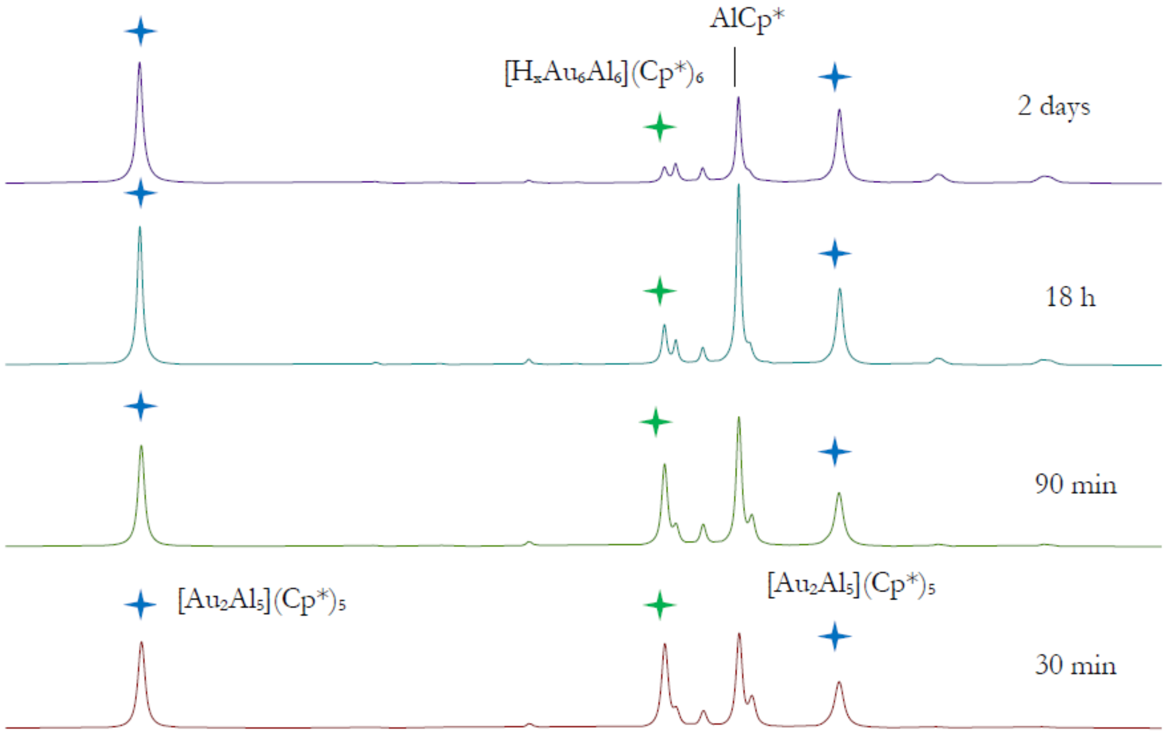

Figure S24: Time-dependant *in situ* ^{1}H-NMR spectra (benzene-d_6) of the reaction [^{i}DippAuH] + AlCp* (1:3, 75 °C). An excess of AlCp* is unnecesarry for the synthesis of **3**.

3.2 Cluster growth and degradation reactions

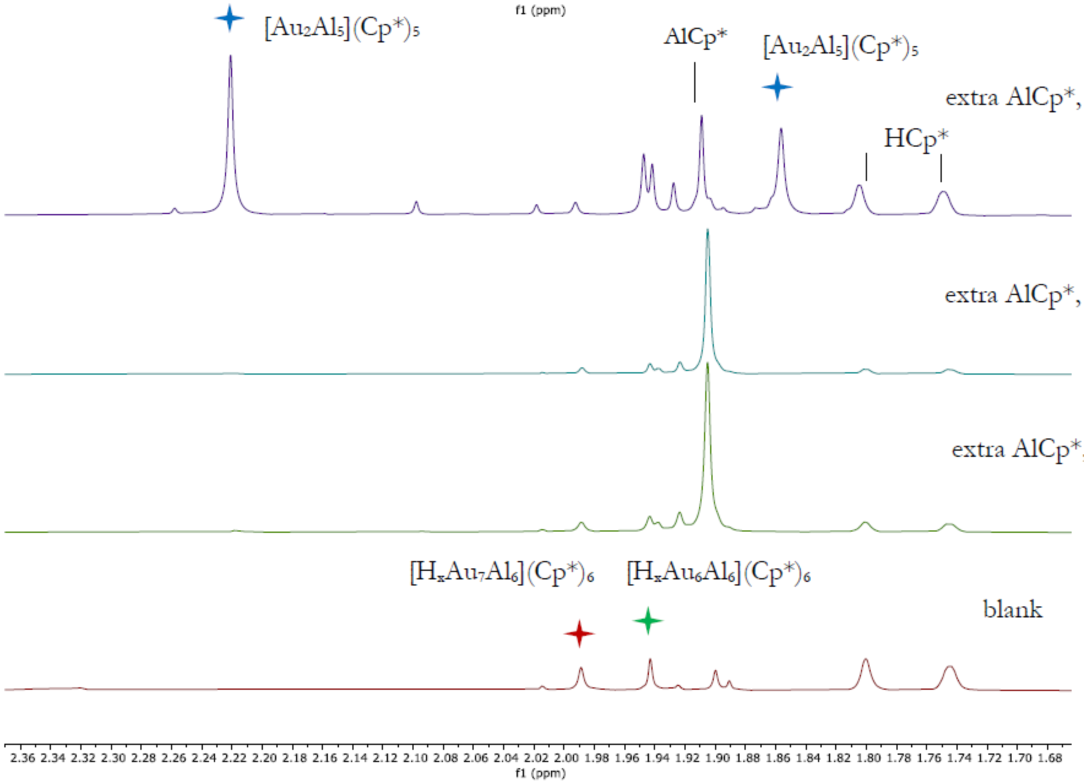

Figure S25: Time dependant *in situ* ^{1}H-NMR spectra (benzene-d$_6$) of the conversion of *in situ* generated [$H_{0\text{-}1}Au_{6/7}Al_6$]($Cp^*$)$_6$ (**1/2**) (blank) with extra AlCp*.

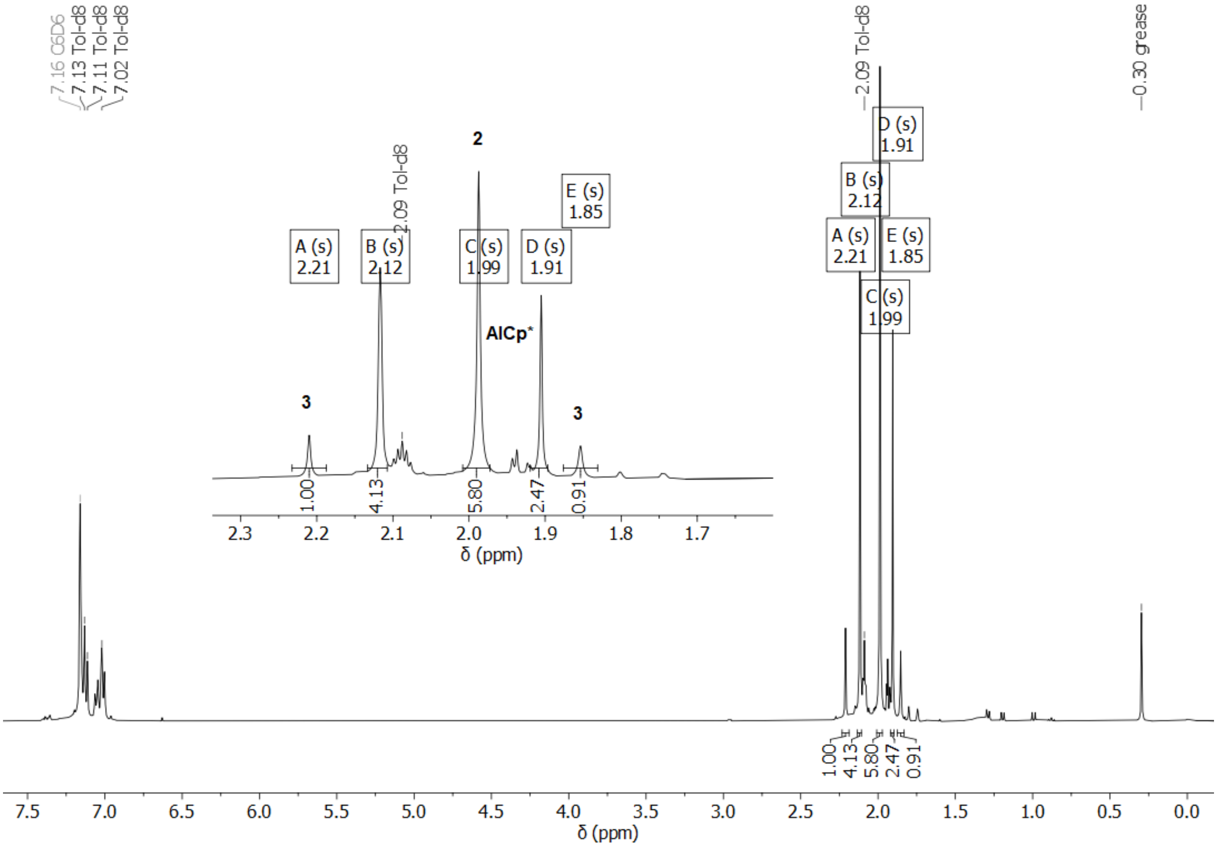

Figure S26: *In situ* ^{1}H-NMR spectra (benzene-d$_6$) of the reaction **2** + AlCp* (1:1, 75 °C, 2 h). The larger cluster **2** degrades towards smaller cluster **3**.

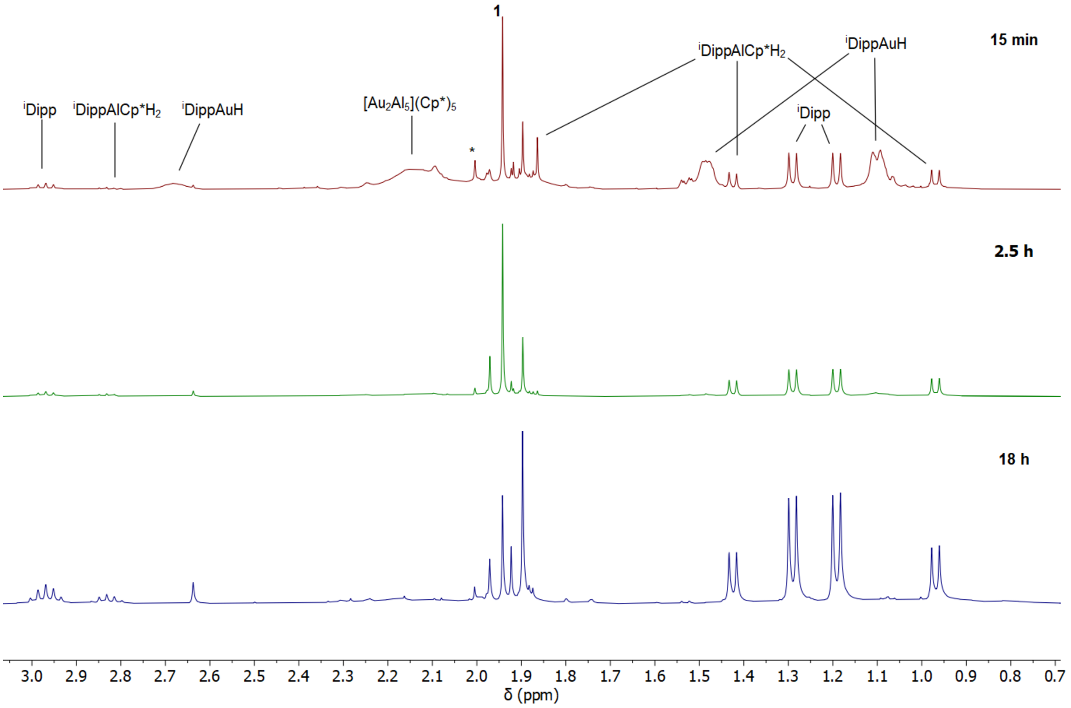

Figure S27: Time-dependant *in situ* ^{1}H-NMR spectra (benzene-d$_6$) of the reaction **3** + ^{i}DippAuH (1:2, r.t.). The cluster **3** serves as building block for the formation of **1/2** upon addition of gold source.

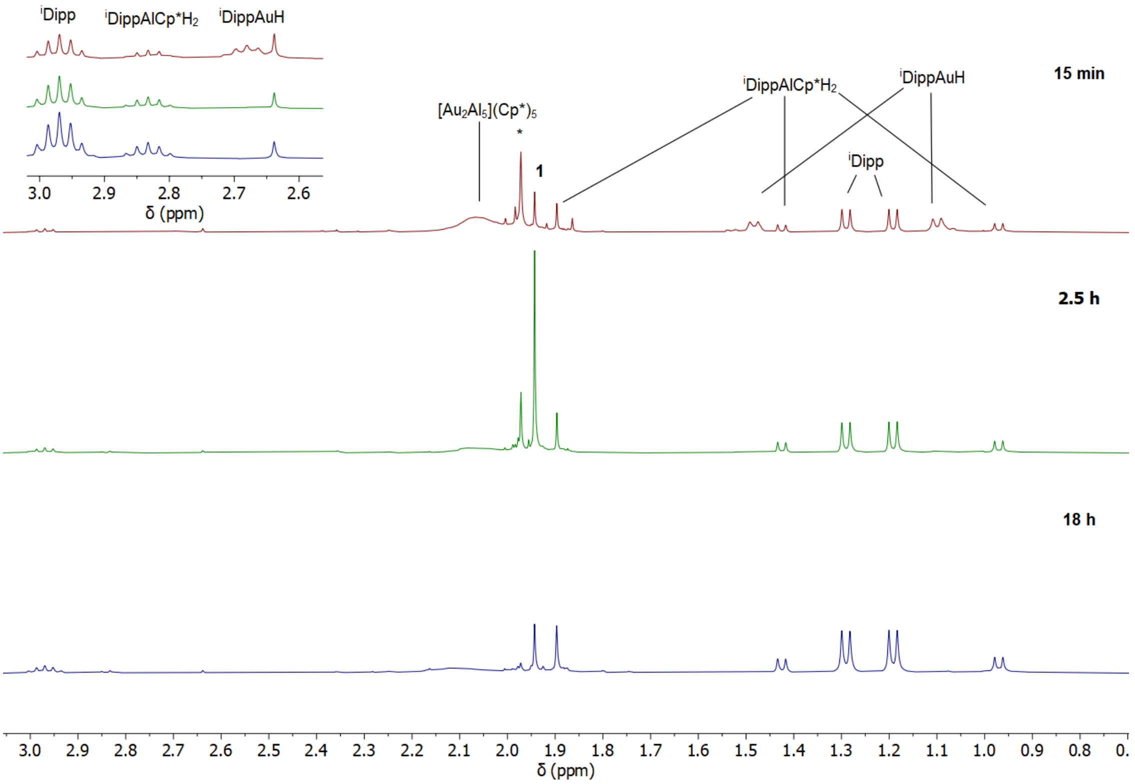

Figure S28 Time-dependant *in situ* ^{1}H-NMR spectra (benzene-d$_6$) of the reaction **3** + ^{i}DippAuH + PPh_3 (1:2:10, r.t.).

3.3 Reduction to Au(0) in the course of the cluster generating reactions

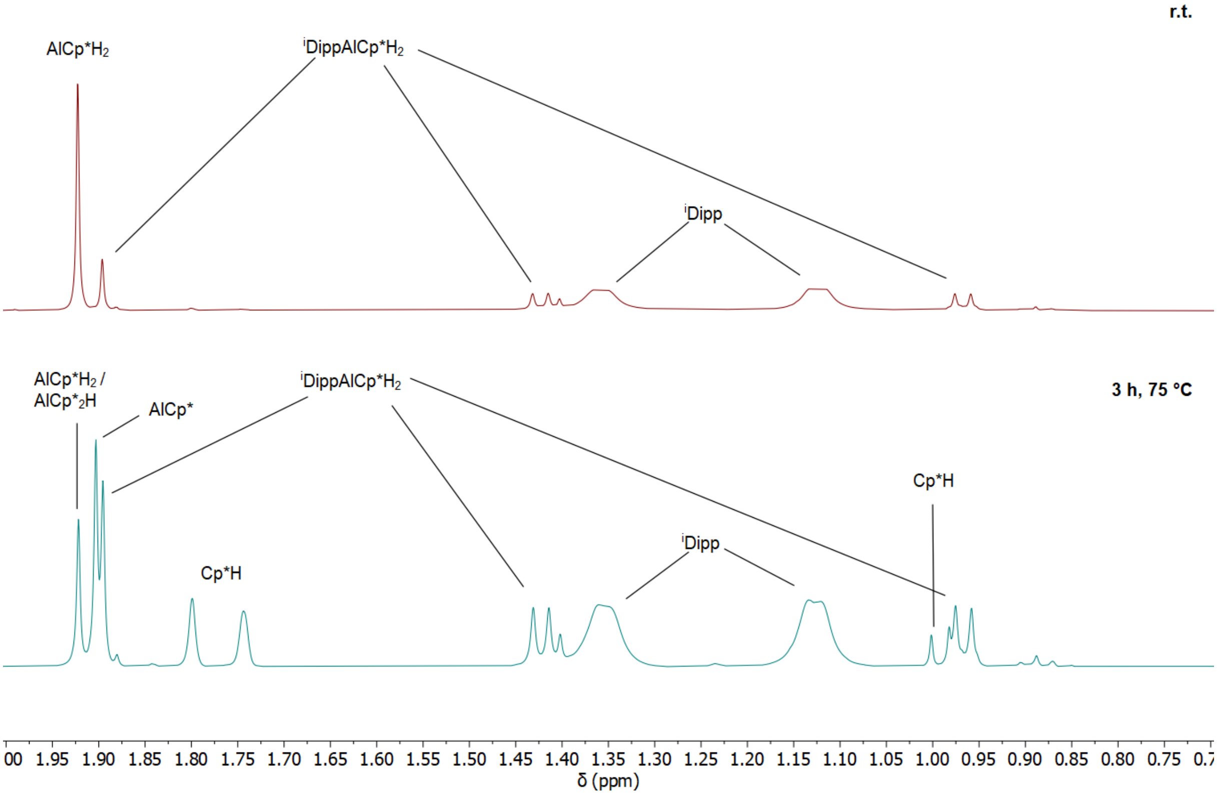

Figure S29: *In situ* ^{1}H-NMR spectra (benzene-d$_6$) of the reaction ^{i}Dipp + $AlCp^{*}H_2$ (1:1) at r.t. (top) and after heating at 75 °C for 3 h (bottom). Different Al-organyls share an equilibrium in solution at elevated temperature.

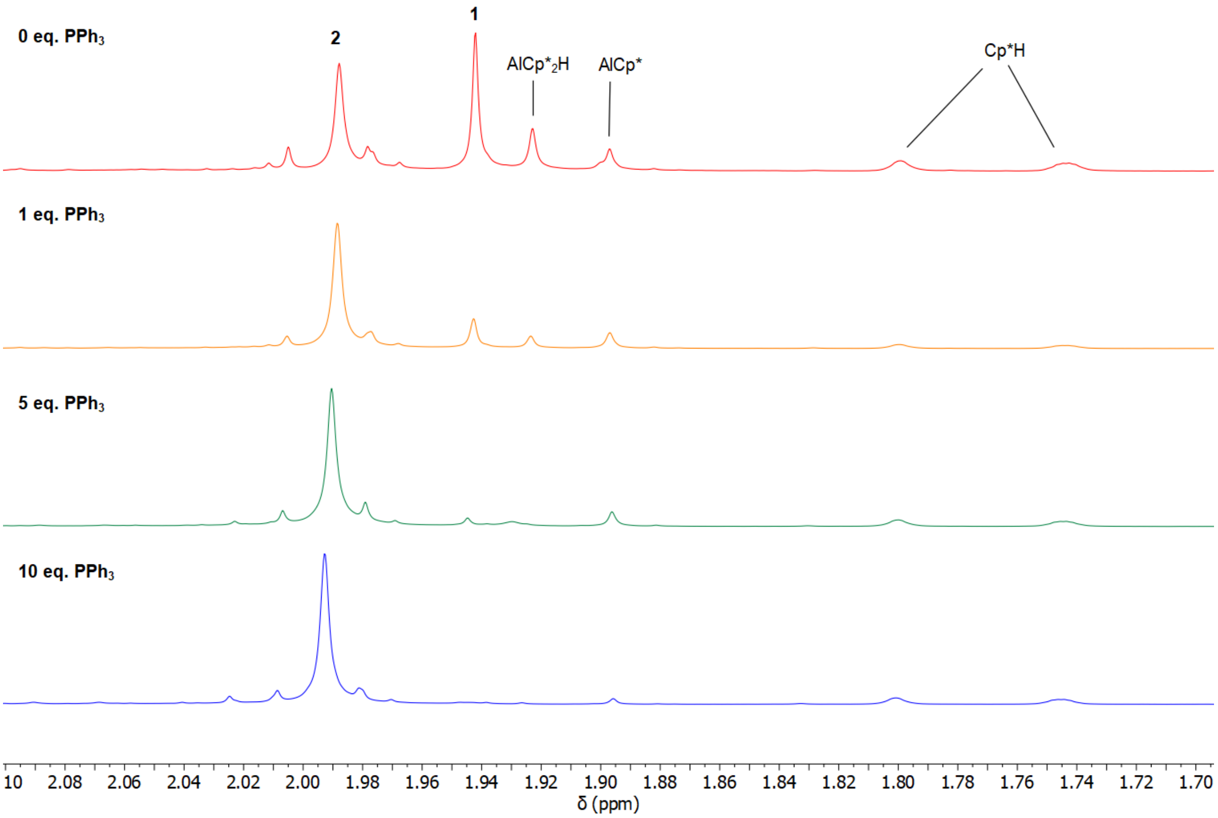

Figure S30: *In situ* ^{1}H-NMR spectra (benzene-d$_6$) of the reaction [^{i}DippAuH] + AlCp* (1:1, 75 °C, 2 h) upon variation of PPh_3 equivalents. Increased PPh_3 concentration leads to selective formation of **2** and suppresses the formation of Al(III) species.

3.4 Influence of PR_3 on the product distribution

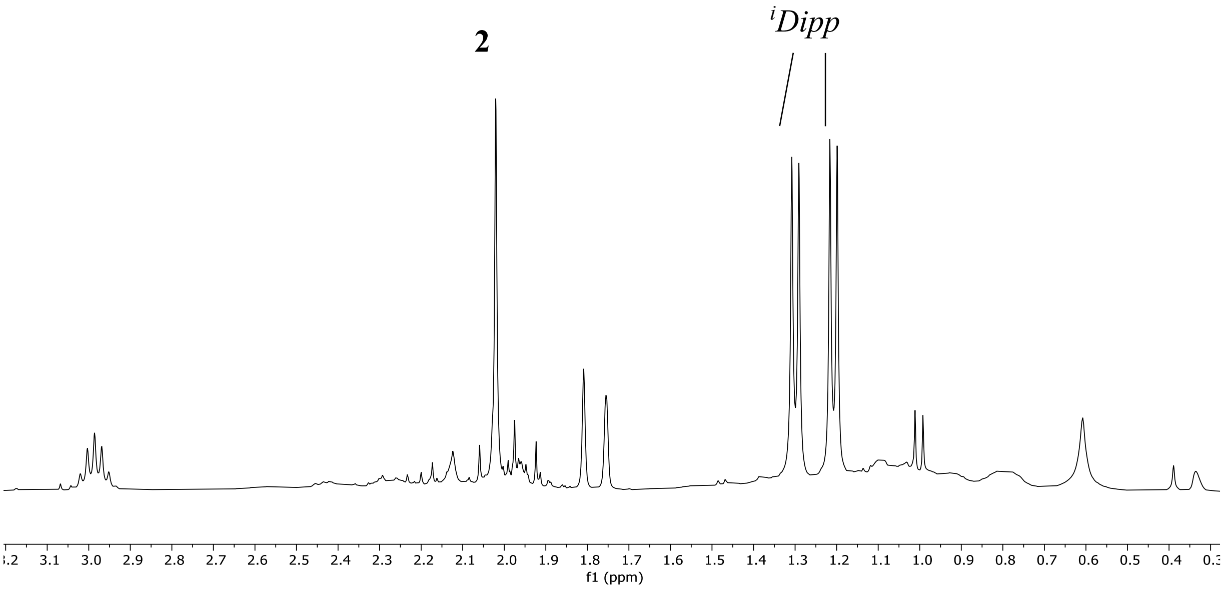

Figure S31: *In situ* ^{1}H-NMR spectra (benzene-d$_6$) of the reaction [^{i}DippAuH] + AlCp* + PPh_3 (1:1:50, 75 °C, 2 h). The cluster **2** is formed selectively at high PPh_3 concentration.

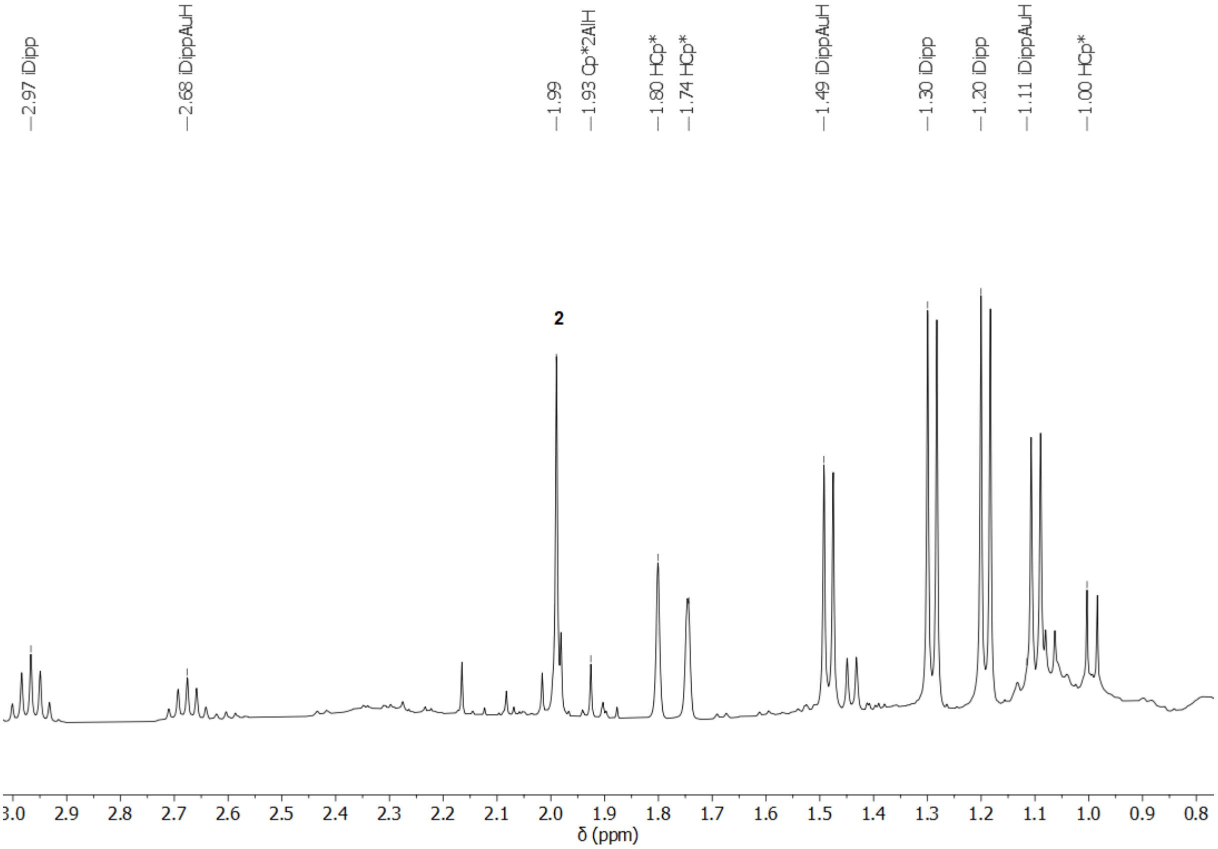

Figure S32: *In situ* ^{1}H-NMR spectra (benzene-d$_6$) of the reaction [^{i}DippAuH] + AlCp* + PPh_3 (1:0.75:1, 75 °C, 2 h). The selective formation of **2** is possible with only 1 eq. of PPh_3, if an excess of ^{i}DippAuH is used.

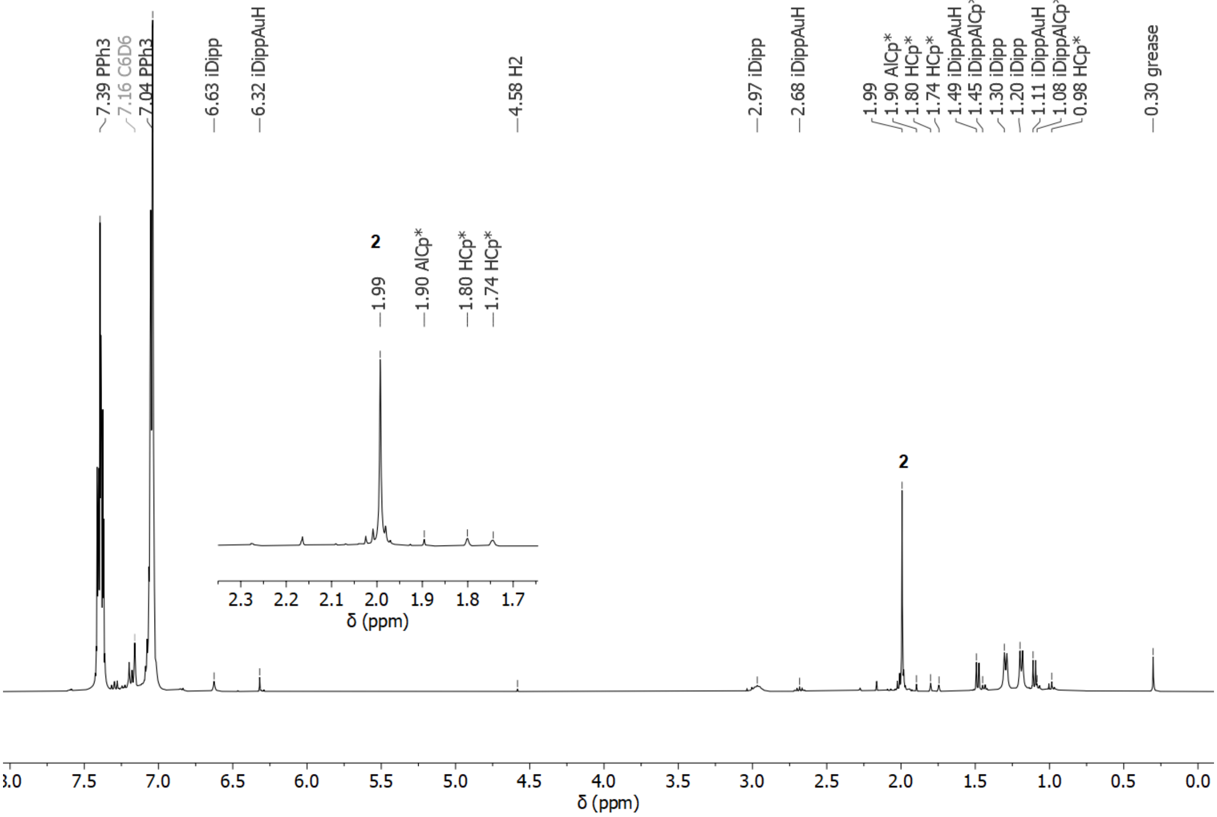

Figure S33: *In situ* ^{1}H-NMR spectra (benzene-d$_6$) of the reaction [^{i}DippAuH] + AlCp* + 10 PPh_3 (1:1, 75 °C, 2 h). Selective formation of **2**.

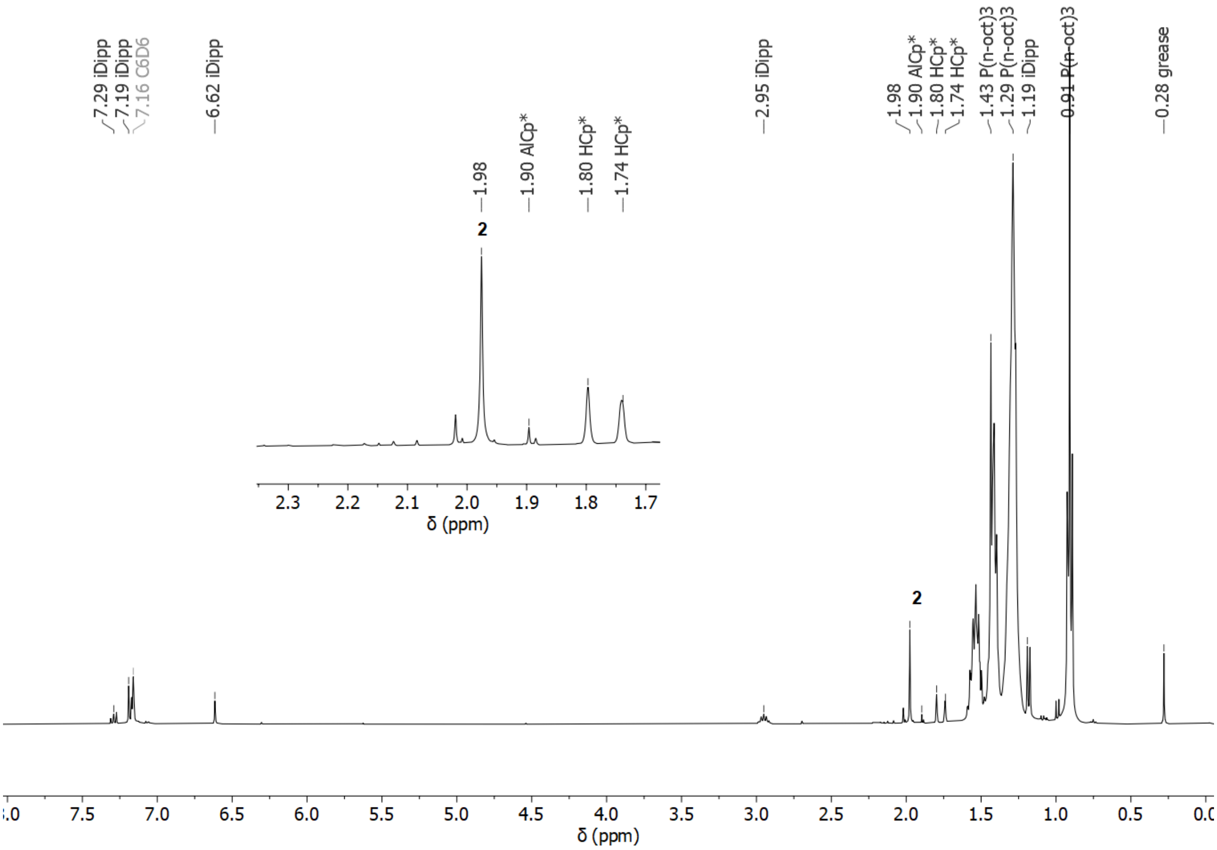

Figure S34: *In situ* ^{1}H-NMR spectra (benzene-d$_6$) of the reaction [^{i}DippAuH] + AlCp* + 10 P(*n*-Oct)$_3$ (1:1, 75 °C, 2 h). Selective formation of **2**.

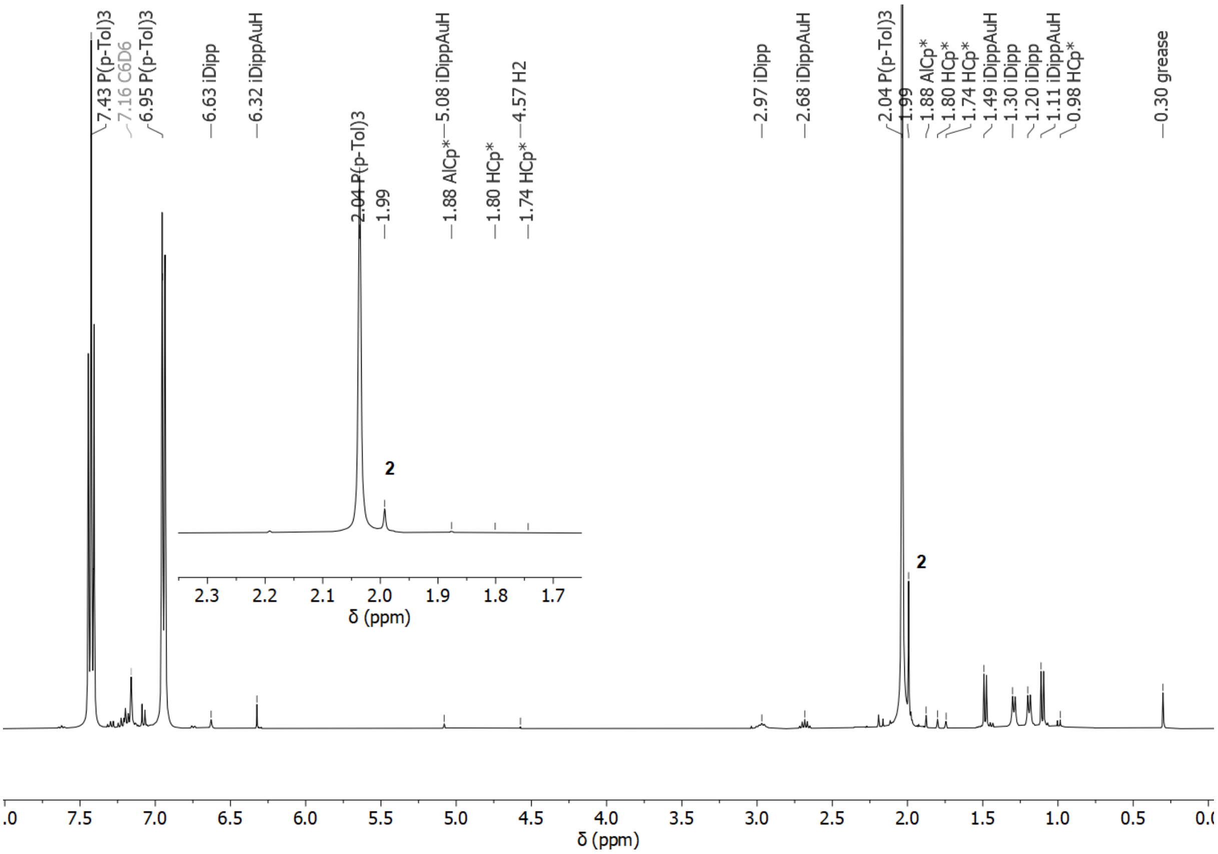

Figure S35: *In situ* ^{1}H-NMR spectra (benzene-d$_6$) of the reaction [^{i}DippAuH] + AlCp* + 10 P(*p*-Tol)$_3$ (1:1, 75 °C, 2 h). Selective formation of **2**.

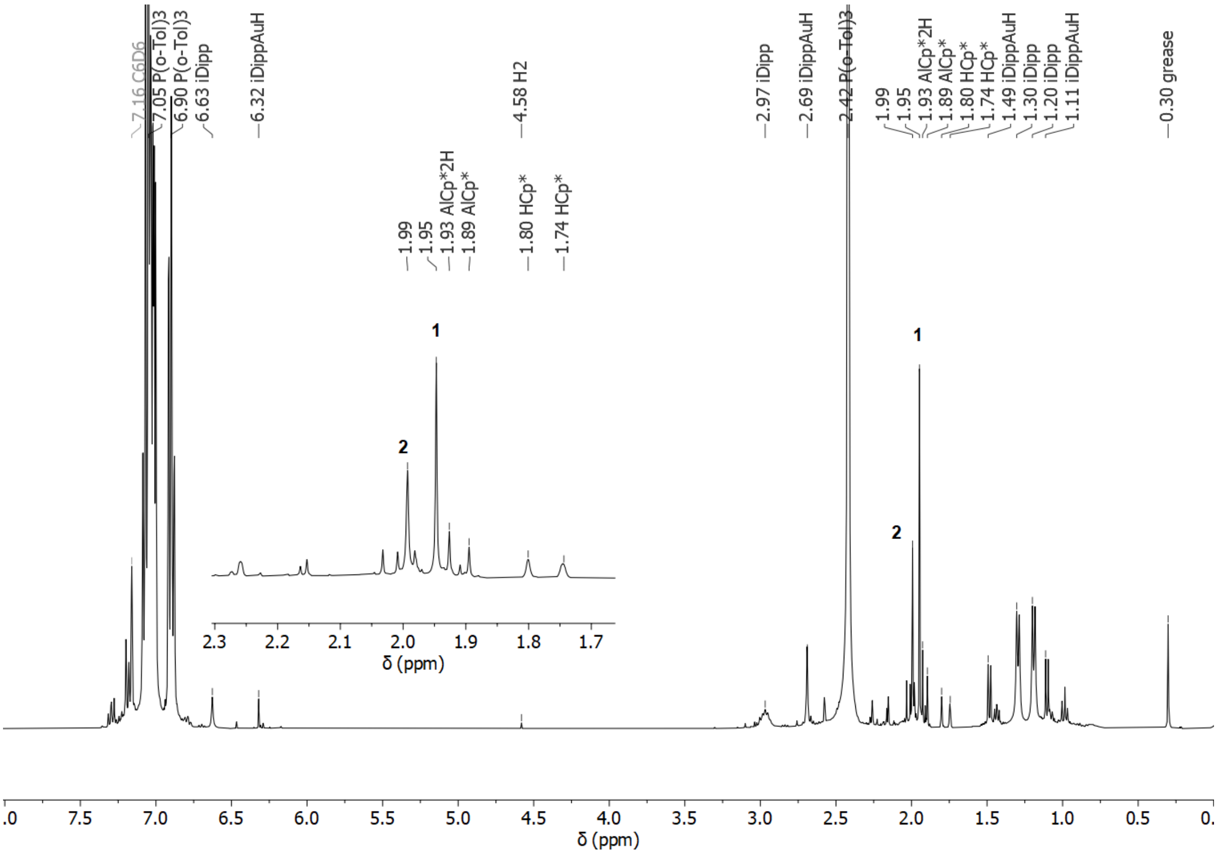

Figure S36: *In situ* ^{1}H-NMR spectra (benzene-d$_6$) of the reaction [^{i}DippAuH] + AlCp* + 10 P(*o*-Tol)$_3$ (1:1, 75 °C, 2 h). The **1**/**2** mixture is formed with bulky P(o-Tol)$_3$.

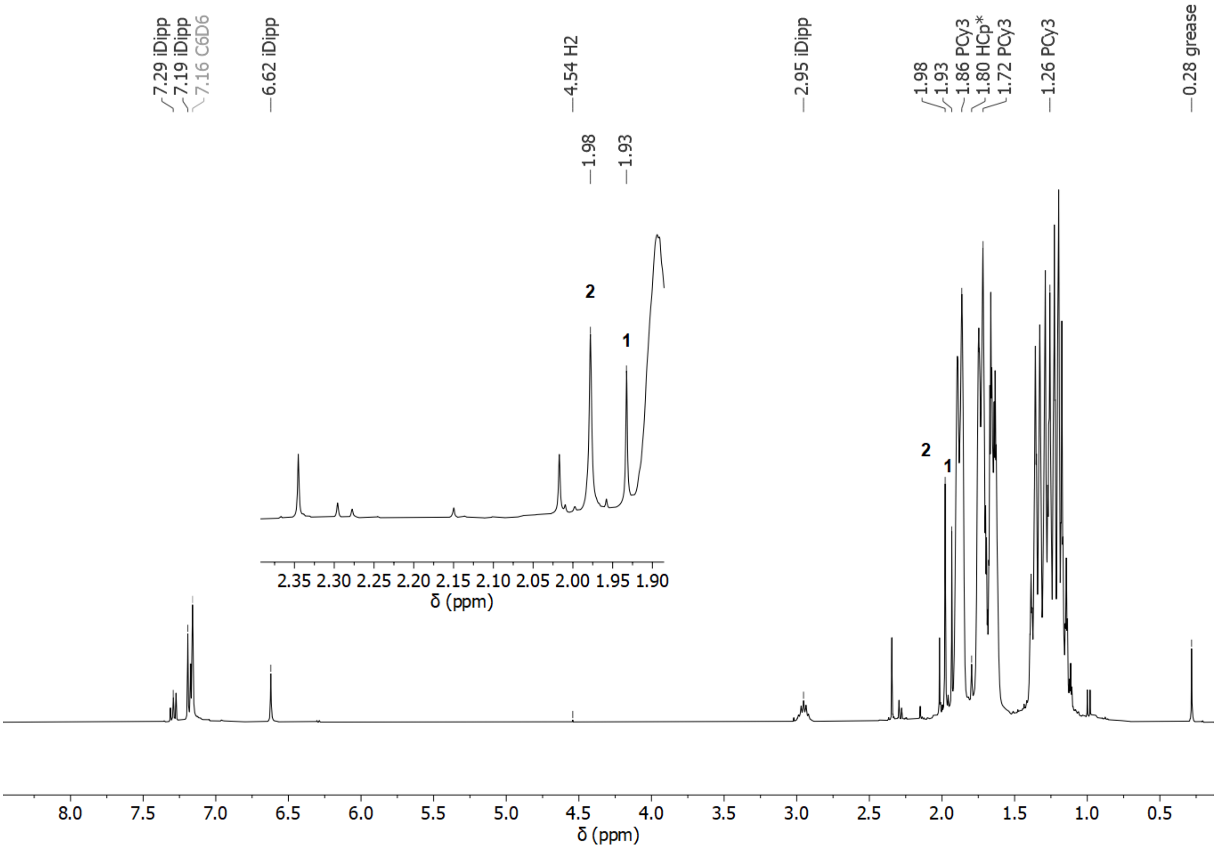

Figure S37: *In situ* ¹H-NMR spectra (benzene-d$_6$) of the reaction [^{i}DippAuH] + AlCp* + 10 P(Cy)$_3$ (1:1, 75 °C, 2 h). The **1/2** mixture is formed with bulky PCy$_3$.

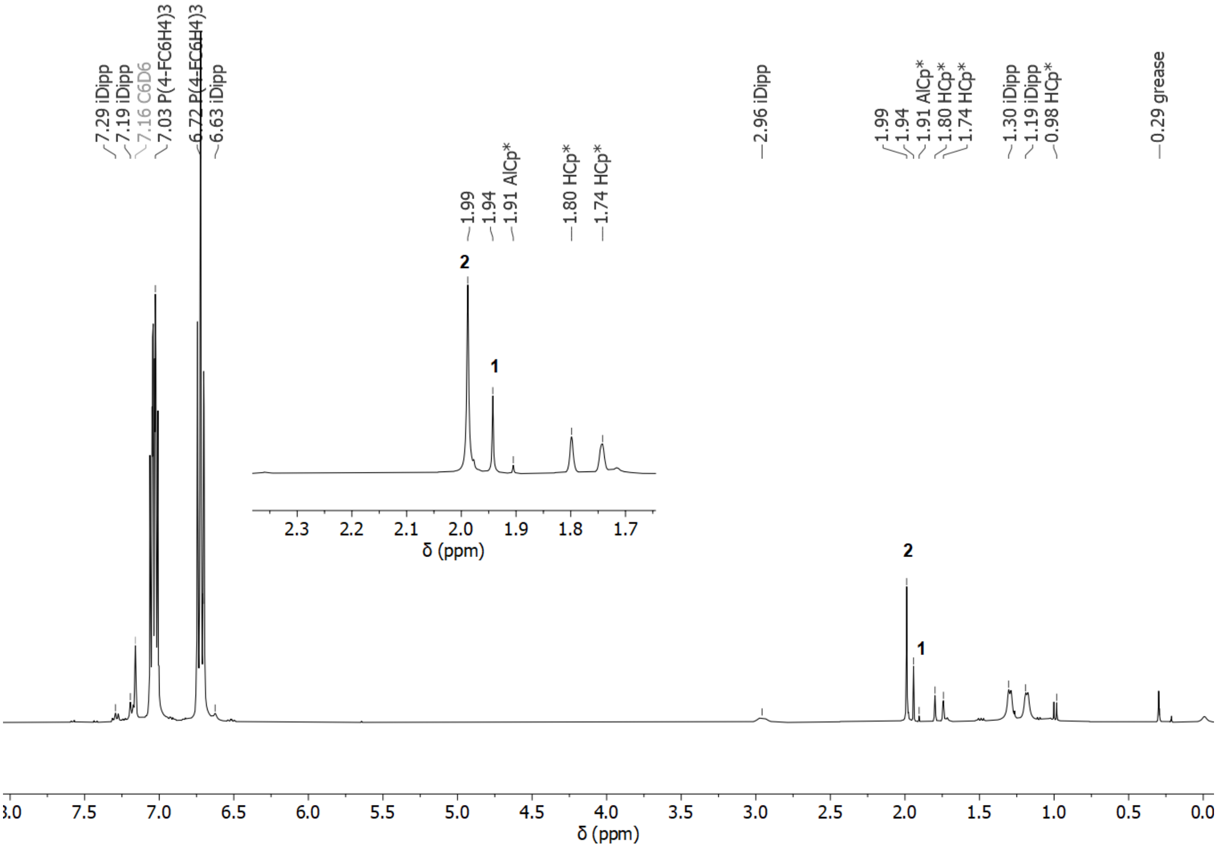

Figure S38: *In situ* ¹H-NMR spectra (benzene-d$_6$) of the reaction [^{i}DippAuH] + AlCp* + 10 P(4-FC$_6$H$_4$)$_3$ (1:1, 75 °C, 2 h). Preferred formation of **2** against **1**.

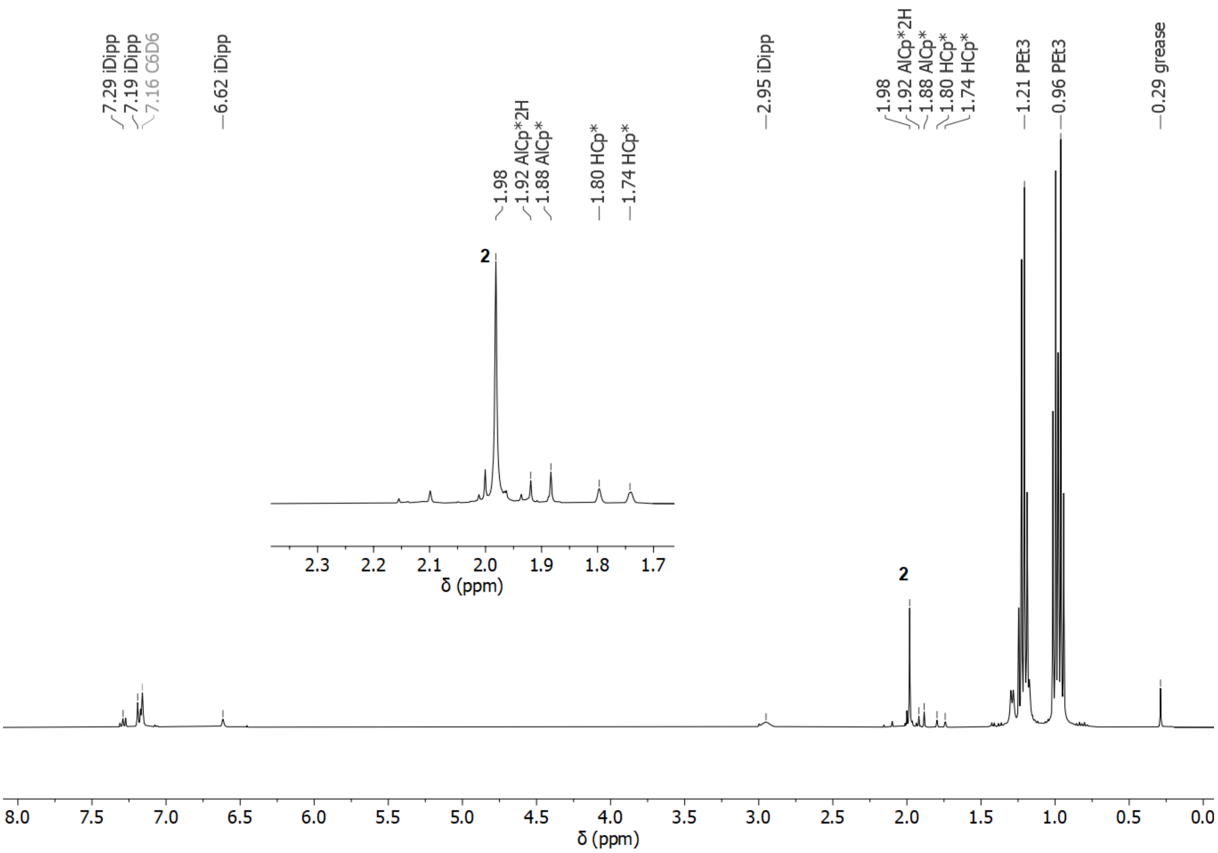

Figure S39: *In situ* ^{1}H-NMR spectra (benzene-d$_6$) of the reaction [^{i}DippAuH] + AlCp* + 10 PEt$_3$ (1:1, 75 °C, 2 h). Selective formation of **2**.

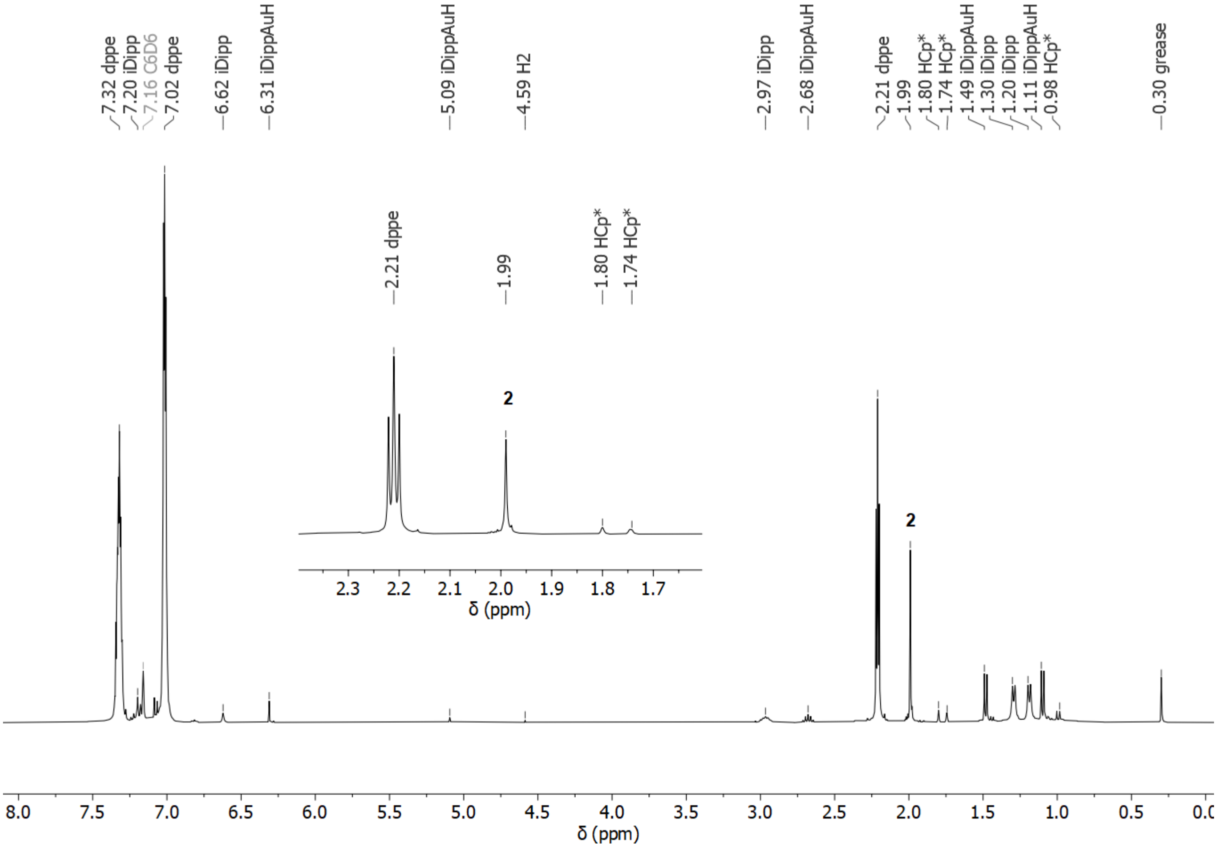

Figure S40: *In situ* ^{1}H-NMR spectra (benzene-d$_6$) of the reaction [^{i}DippAuH] + AlCp* + 5 dppe (1:1, 75 °C, 2 h). Selective formation of **2**.

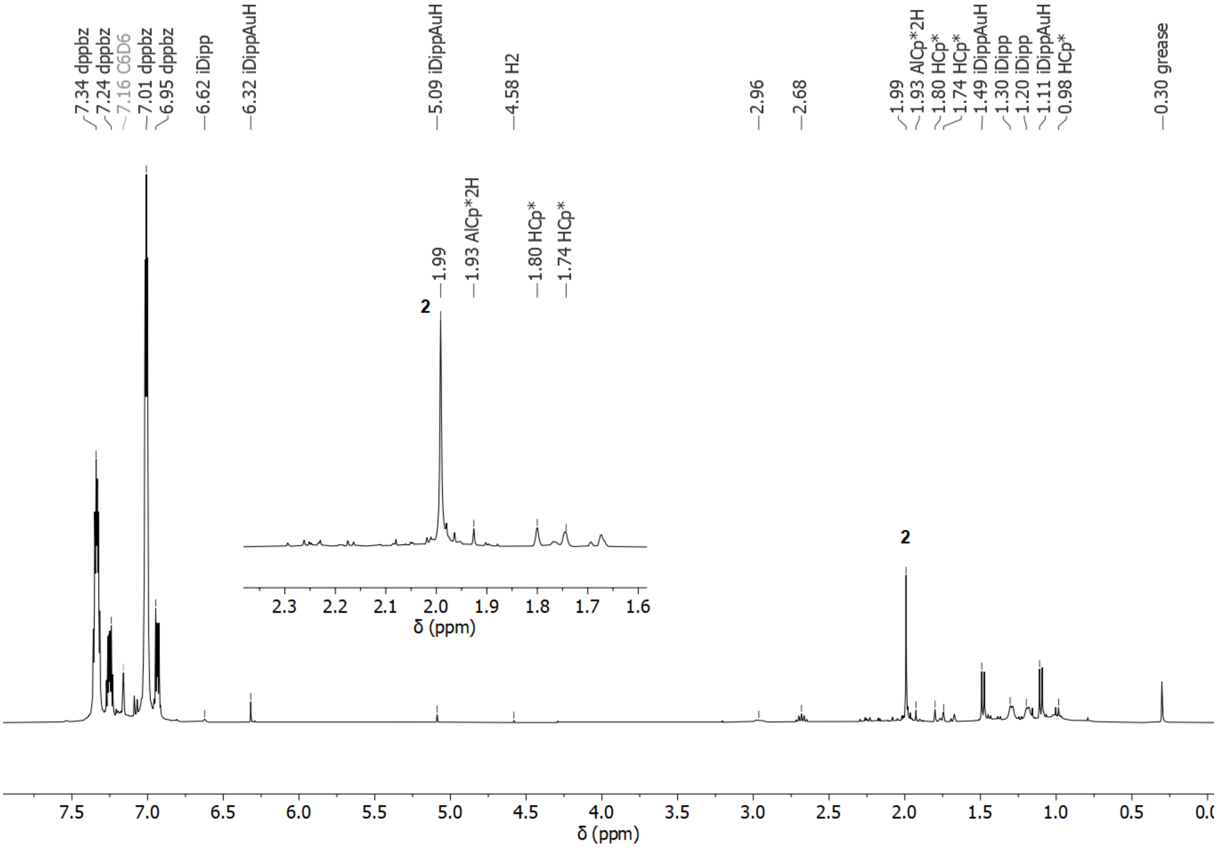

Figure S41 *In situ* ^{1}H-NMR spectra (benzene-d$_6$) of the reaction [^{i}DippAuH] + AlCp* + 5 dppbz (1:1, 75 °C, 2 h). Selective formation of **2**.

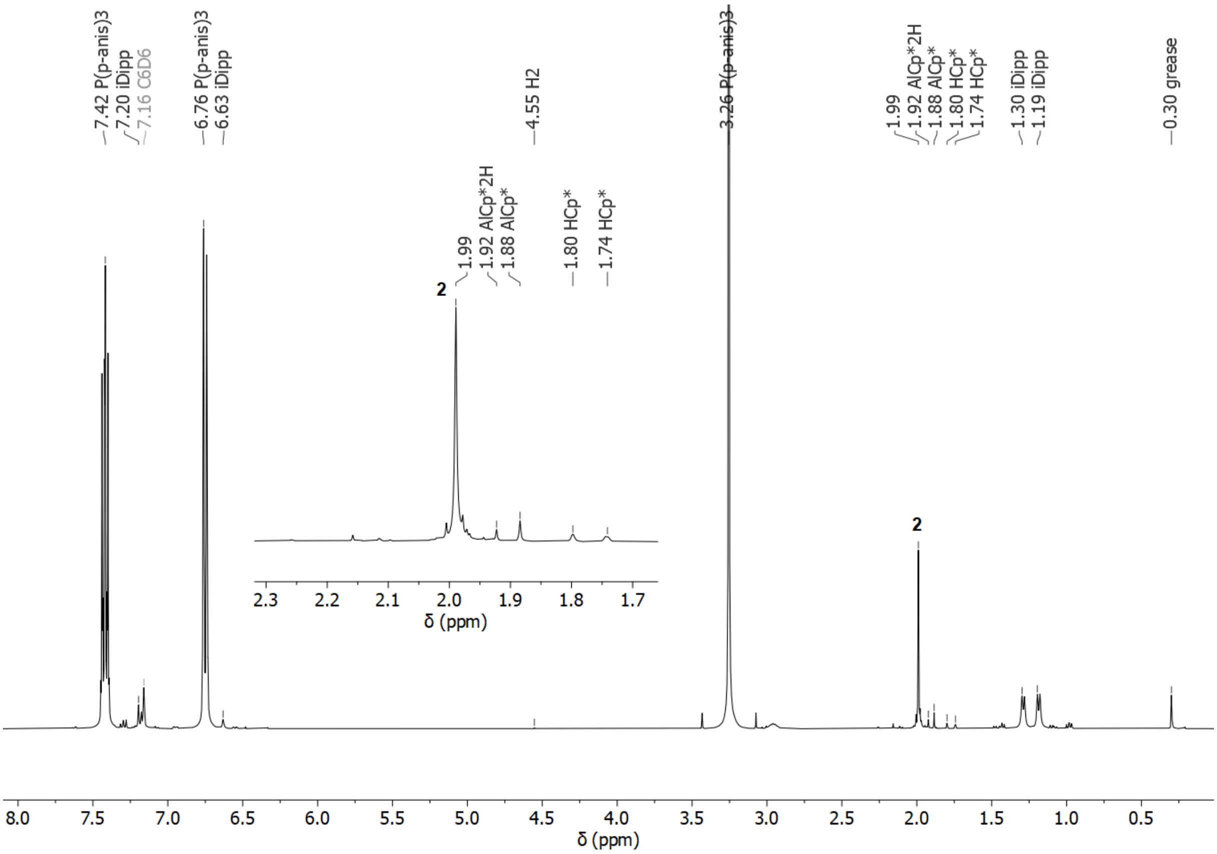

Figure S42: *In situ* ^{1}H-NMR spectra (benzene-d$_6$) of the reaction [^{i}DippAuH] + AlCp* + 10 P(*p*-anis)$_3$ (1:1, 75 °C, 2 h). Selective formation of **2**.

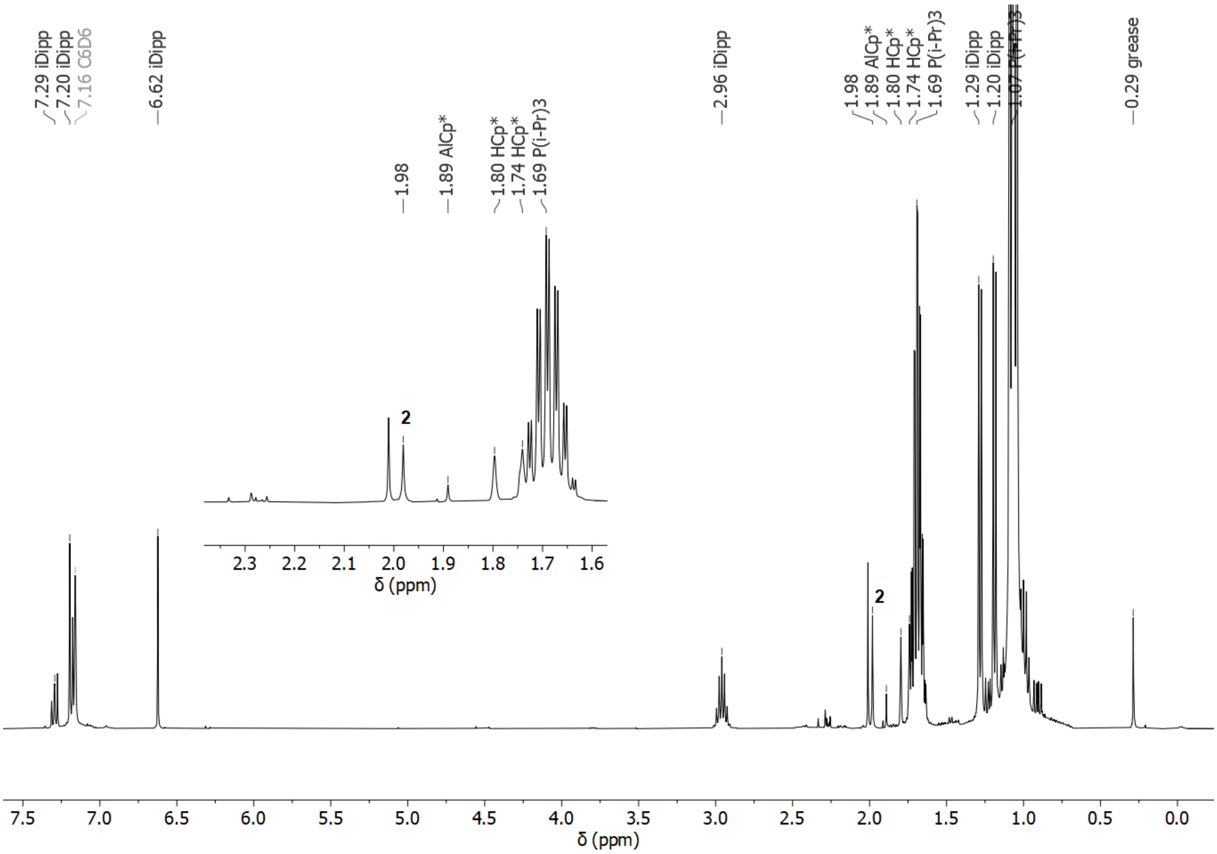

Figure S43: *In situ* ^{1}H-NMR spectra (benzene-d$_6$) of the reaction [^{i}DippAuH] + AlCp* + 10 P(i-Pr)$_3$ (1:1, 75 °C, 2 h). Selective formation of **2**.

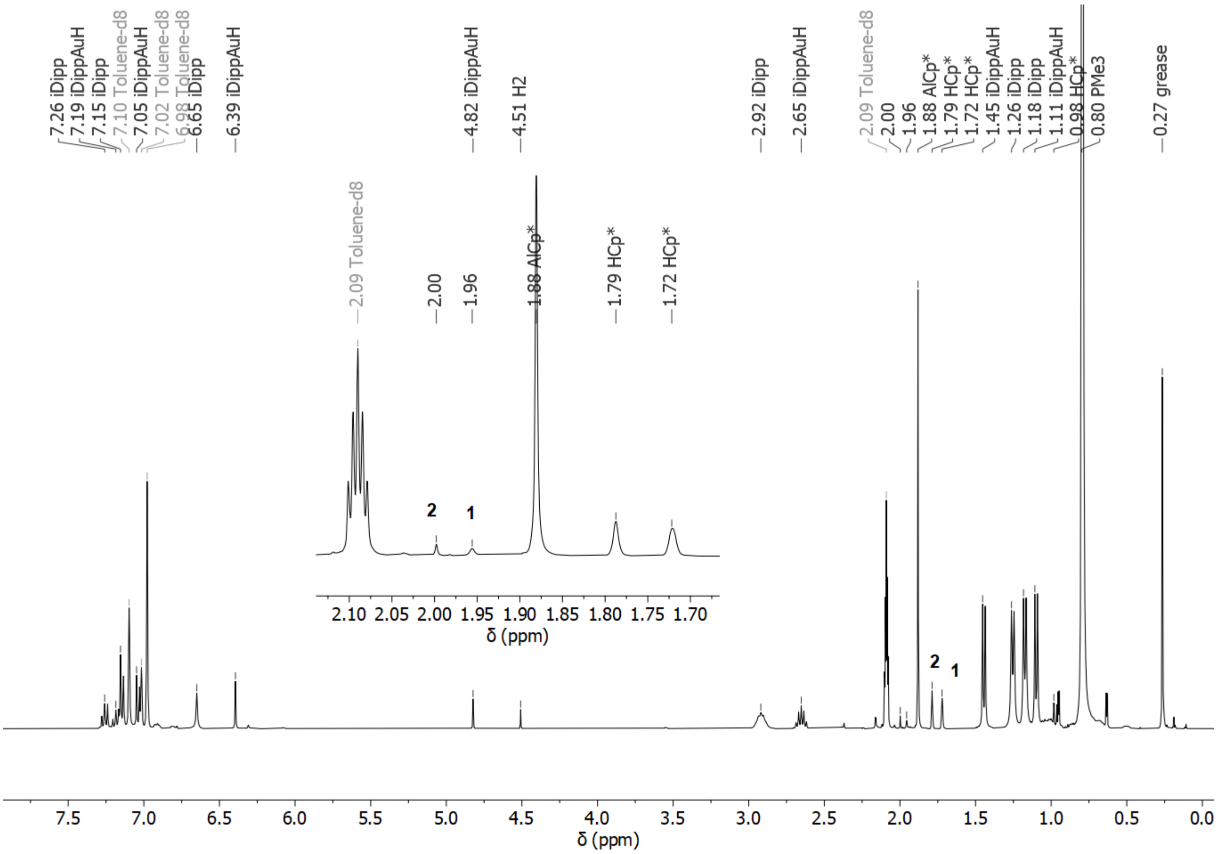

Figure S44: *In situ* ^{1}H-NMR spectra (toluene-d$_8$) of the reaction [^{i}DippAuH] + AlCp* + 10 PMe$_3$ (1:1, 75 °C, 2 h). The clusters **1** and **2** are formed as side products, gold mirror was observed.

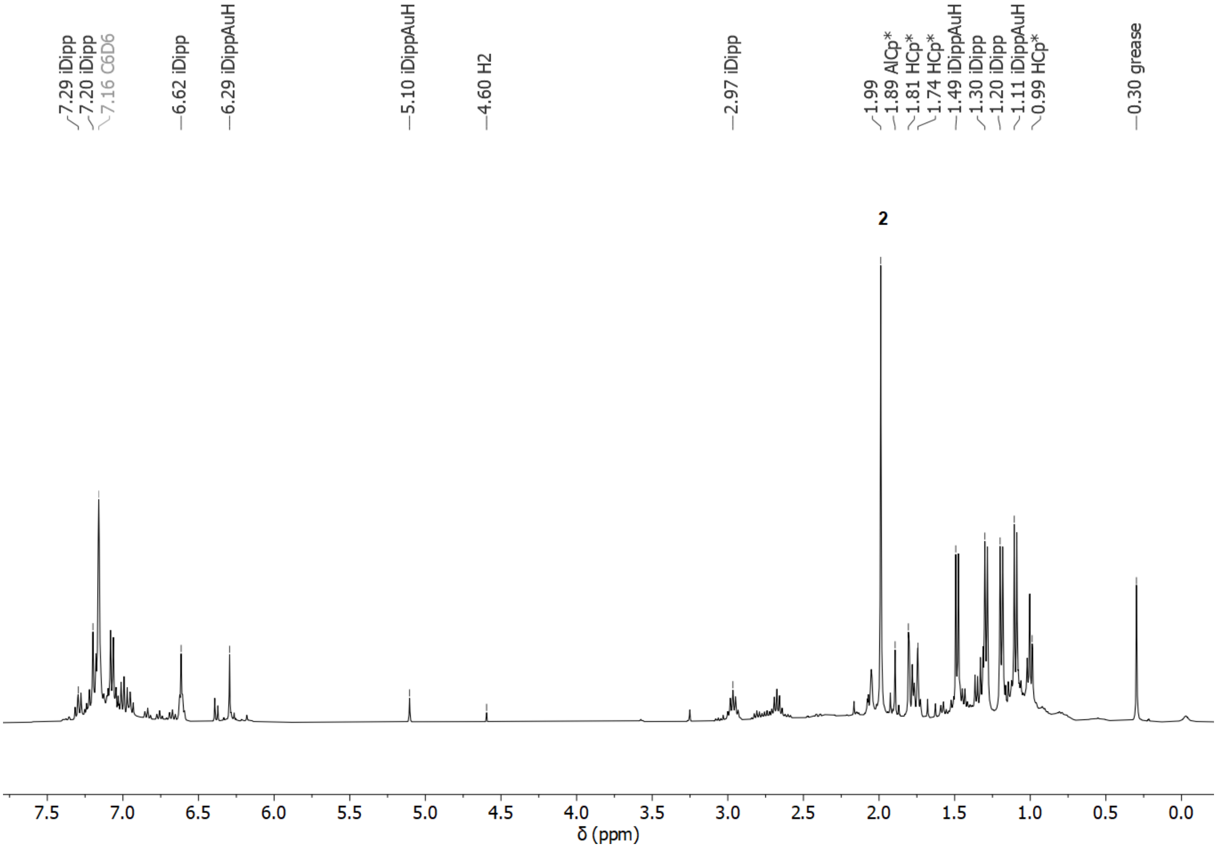

Figure S45: *In situ* ^{1}H-NMR spectra (benzene-d_6) of the reaction [^{i}DippAuH] + AlCp* + 1 $P(OPh)_3$ (1:1, 75 °C, 2 h). The cluster **2** is a major reaction product, but various decomposition products are present.

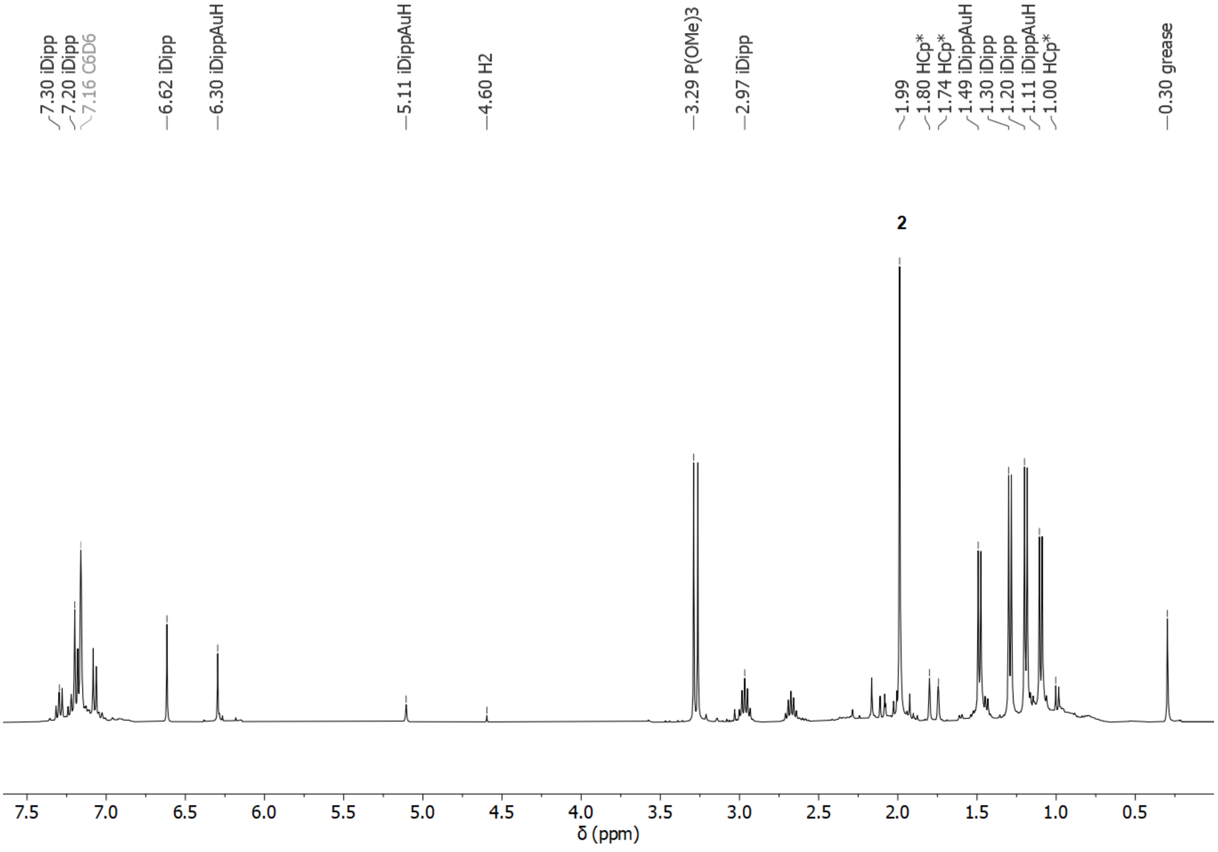

Figure S46: *In situ* ^{1}H-NMR spectra (benzene-d_6) of the reaction [^{i}DippAuH] + AlCp* + 1 $P(OMe)_3$ (1:1, 75 °C, 2 h). The cluster **2** is a major reaction product, but various decomposition products are present.

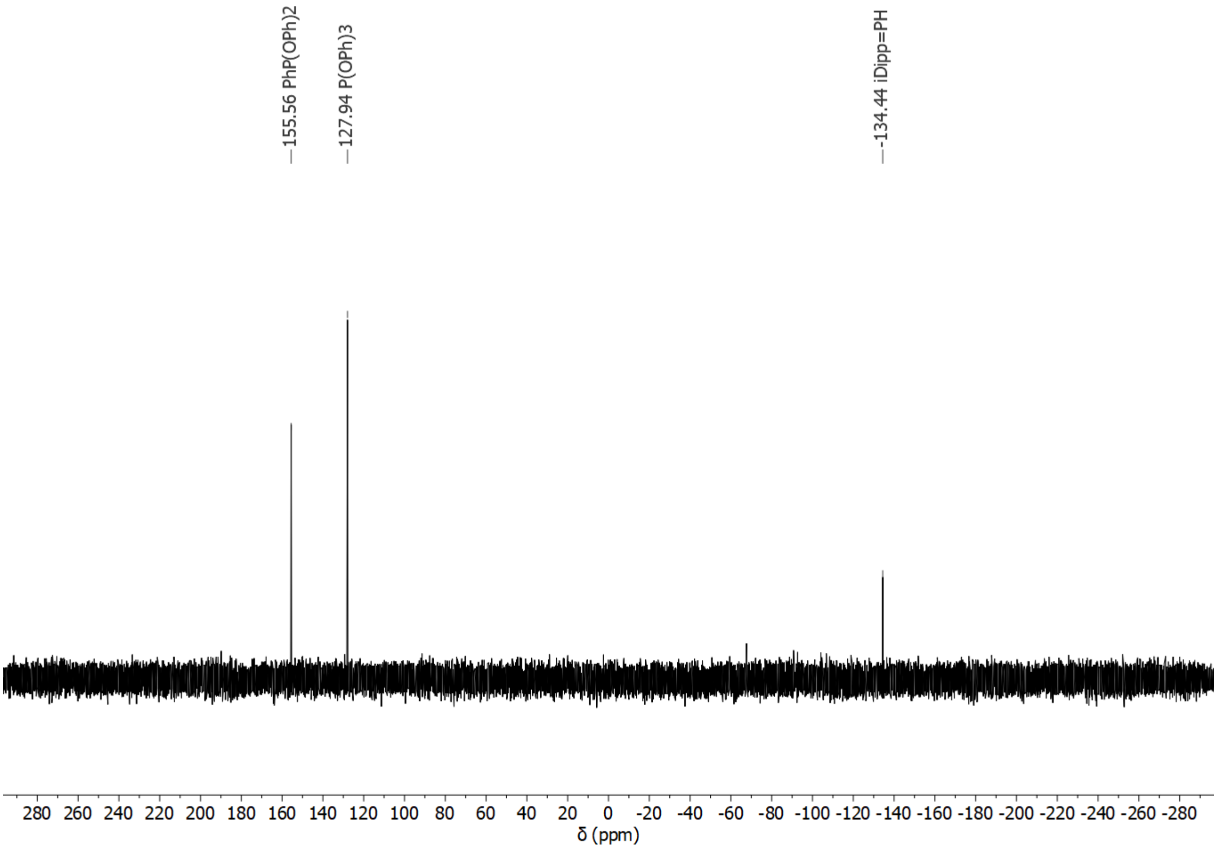

Figure S47: *In situ* ^{31}P-NMR spectra (benzene-d_6) of the reaction [^{i}DippAuH] + AlCp* + 1 $P(OPh)_3$ (1:1, 75 °C, 2 h).

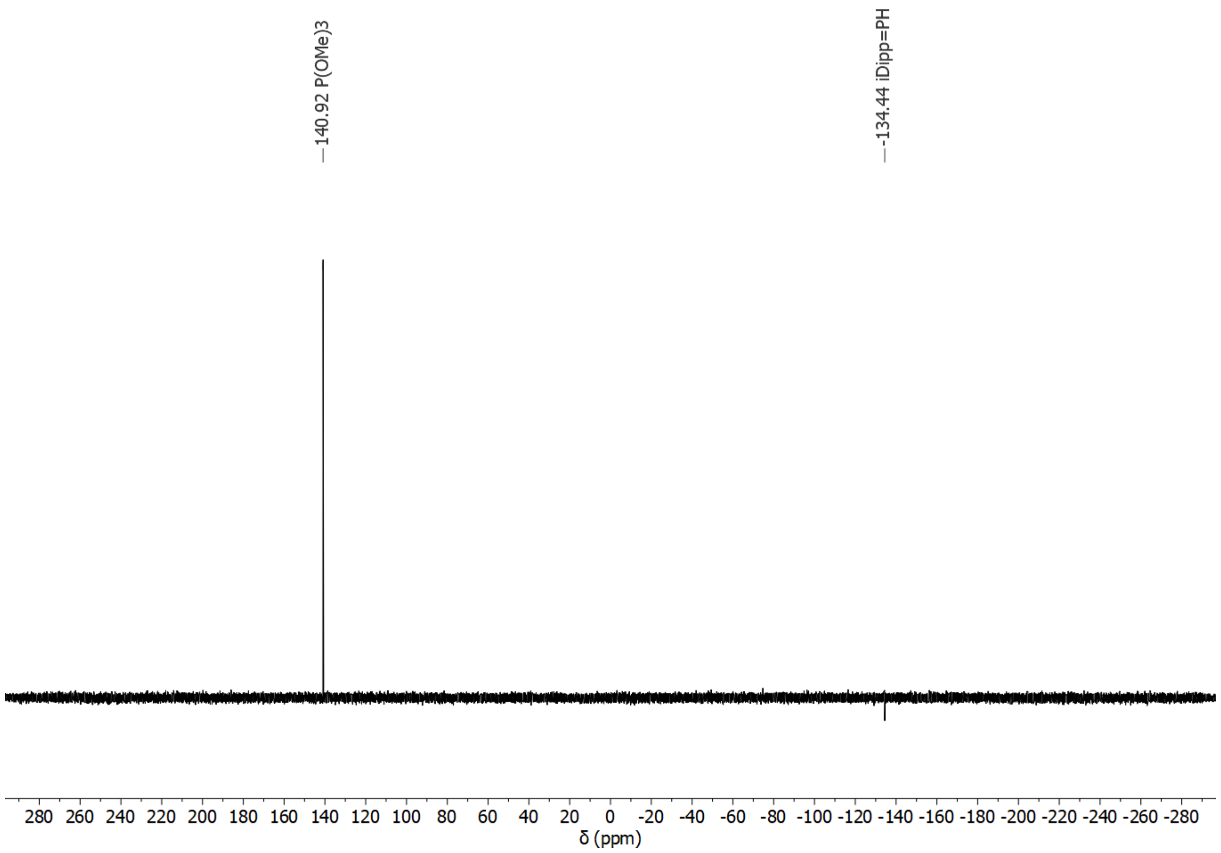

Figure S48: *In situ* ^{31}P-NMR spectra (benzene-d$_6$) of the reaction [^{i}DippAuH] + AlCp* + 1 $P(OMe)_3$ (1:1, 75 °C, 2 h).

4. Synthesis of starting materials

Synthesis of ^{i}DippAuH and AlCp* were performed according to the literature known procedures.[1, 2]

Synthesis of $LiBEt_3D$

Synthesis of $LiBEt_3D$ was performed according to the literature procedure.[3] LiD (40 mg, 4.47 mmol, 1.49 eq.) was stirred in 1.0 M solution of BEt_3 (3 mL, 3.00 mmol, 1 eq.) in THF for 24 h at r.t. The resulting suspension was filtered through syringe filter giving a clear solution. This solution was titrated with 0.1 M HCl and phenolphthalein as an indicator, the found concentration of $LiBEt_3D$ was 0.82 M. For the next synthesis 0.55 mL of this solution was diluted with 4.5 mL THF.

Synthesis of ^{i}DippAuD

Synthesis of ^{i}DippAuD was performed according to modified literature procedure.[1] ^{i}DippAuCl (250 mg, 0.40 mmol, 1.0 eq.) was dissolved in 15 mL THF and cooled to – 78 °C. Then a solution of $LiBEt_3D$ (5.05 mL, 0.44 mmol, 1.1 eq.) was slowly added under exclusion of light.

The dark reaction mixture was stirred for 1 h and then wormed up to – 17 °C for 1h (ice/salt mixture) and at the end stirred at 22°C for 3 h. The solvent was removed *in vacuo* and the crude product was extracted with benzene (10 + 5 + 5 mL) and filtered through celite. The resulting dark red solution was stirred over 0.2 mL quicksilver, in order to remove gold nanoparticles. Careful decantation leads to almost colourless solution, which was dried in vacuo giving off white product (162 mg, 0.28 mmol, 69%).

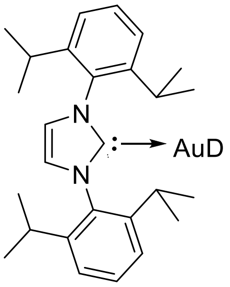

$C_{27}H_{36}DAuN_2$
M= 587,58 g/mol

^{1}H NMR (400 MHz, Benzene-d_6) δ 7.21 (t, $^3J_{HH}$ = 7.8 Hz, 2H, *p*-ArH), 7.07 (d, $^3J_{HH}$ = 7.8 Hz, 4H, *m*-ArH), 6.30 (s, 2H, NCH), 5.10 (s, 0.14H, AuH), 2.68 (sept, $^3J_{HH}$ = 6.8 Hz, 4H, CH), 1.48 (d, $^3J_{HH}$ = 6.8 Hz, 12H, CH_3), 1.11 (d, $^3J_{HH}$ = 6.8 Hz, 12H, CH_3).

Synthesis of (PPh_3)AuCp*

Synthesis of (PPh_3)AuCp* was performed according to modified literature procedure.[4] (PPh_3)AuCl (440 mg, 0.89 mmol, 1.0 eq.) was dissolved in 15 mL THF and cooled to – 78 °C and was slowly added to a suspension of KCp* (155 mg, 0.89 mmol, 1.0 eq.) in 20 mL THF at – 78 °C. The yellow reaction mixture was stirred for 2 h and then at – 42 °C for 30 min and at the end at 0 °C for 1 h. The reaction mixture was filtered, and the solvent was removed *in vacuo* giving the yellow raw product. It was dissolved in 20 mL toluene and filtered, the solvent was removed affording pure (PPh_3)AuCp* as a yellow solid (318 mg, 0.53 mmol, 60%). The target compound is very air- moisture and light sensitive as well as thermal instable even at – 32 °C.

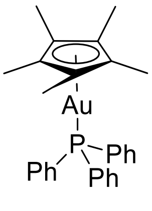

$C_{28}H_{30}AuP$
M = 594,49 g/mol

^{1}H NMR (400 MHz, Benzene-d_6) δ 7.37 – 7.28 (m, 6H, Ph), 6.94 – 6.86 (m, 9H, Ph), 2.40 (d, $^4J_{HP}$ = 1.6 Hz, 15H, CH_3).

^{13}C NMR (101 MHz, Benzene-d_6) δ 134.42 (d, $^2J_{CP}$ = 13.9 Hz, Ph), 131.80 (d, $^1J_{CP}$ = 48.7 Hz, Ph), 130.91 (d, $^4J_{CP}$ = 2.4 Hz, Ph), 128.98 (d, $^3J_{CP}$ = 10.7 Hz), 119.96 (d, $^2J_{CP}$ = 10.6 Hz, Cp*), 13.91 (d, $^3J_{CP}$ = 1.5 Hz, CH_3).

^{31}P{^{1}H} NMR (162 MHz, Benzene-d_6) δ 36.29.

Synthesis of (PCy$_3$)AuO*t*-Bu

(PCy$_3$)AuCl (500 mg, 0.98 mmol, 1.00 eq.) and KO*t*-Bu (115 mg, 1.02 mmol, 1.05 eq.) were suspended in 25 mL toluene and stirred for 2 h at r.t. The reaction mixture was filtered through celite, grown back with 20 mL toluene. The solvent was removed *in vacuo* giving (PCy$_3$)AuO*t*-Bu as a white powder (480 mg, 0.87 mmol, 89%).

(PCy$_3$)AuO*t*-Bu $C_{22}H_{42}AuOP$
M = 550,52 g/mol

^{1}H NMR (400 MHz, Benzene-d_6) δ 1.85 (s, 9H, *t*-Bu), 1.77 – 1.68 (m, 6H), 1.65 – 1.51 (m, 9H), 1.50 – 1.39 (m, 3H), 1.28 – 1.14 (m, 6H), 1.05 – 0.87 (m, 9H).

^{31}P{^{1}H} NMR (162 MHz, Benzene-d_6) δ 45.16.

^{13}C{^{1}H} NMR (101 MHz, Benzene-d_6) δ 72.28 (d, $^3J_{CP}$ = 2.2 Hz, *C*(CH$_3$)$_3$), 37.79 (d, $^4J_{CP}$ = 1.8 Hz, C(*C*H$_3$)$_3$), 33.33 (d, $^1J_{CP}$ = 30.9 Hz, C1), 30.83 (s, C3), 27.18 (d, $^2J_{CP}$ = 11.7 Hz, C2), 26.23 (s, C4).

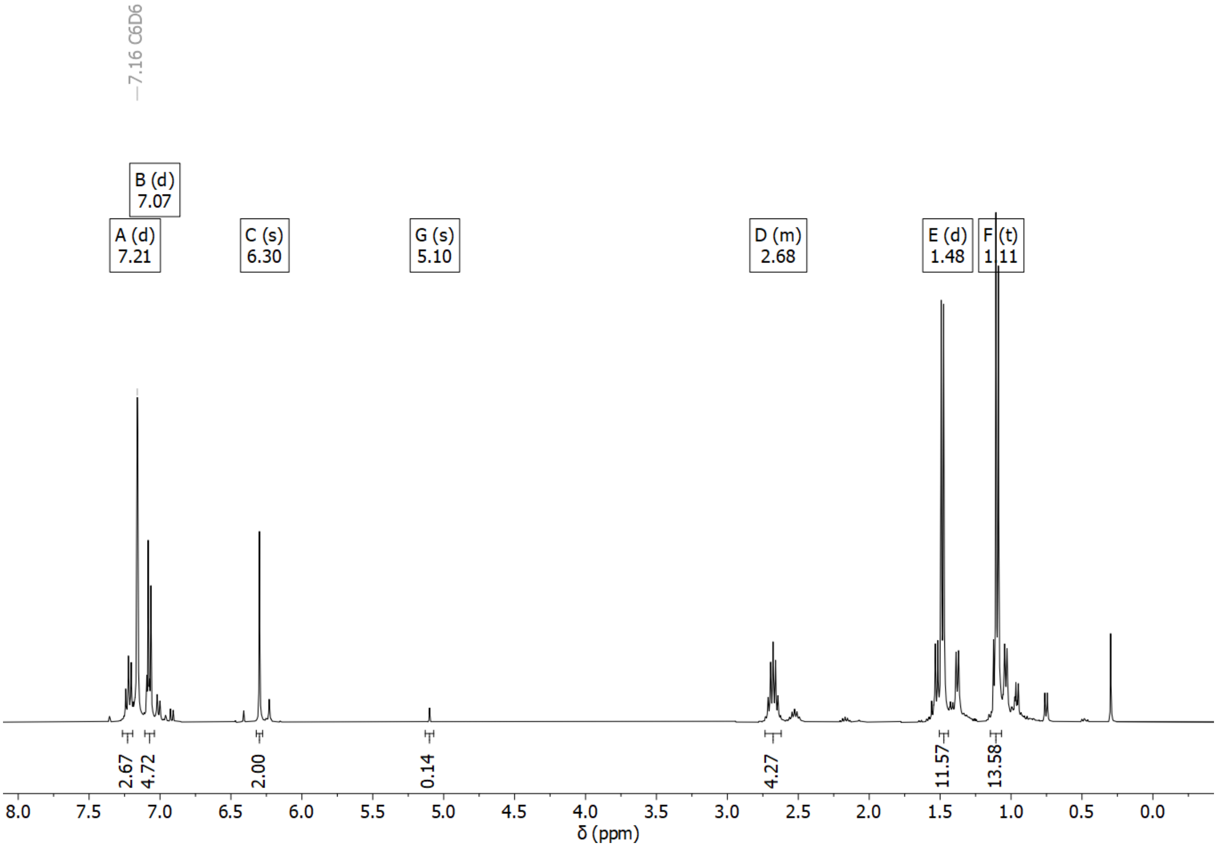

Figure S49: ^{1}H-NMR spectrum of ^{i}DippAuD.

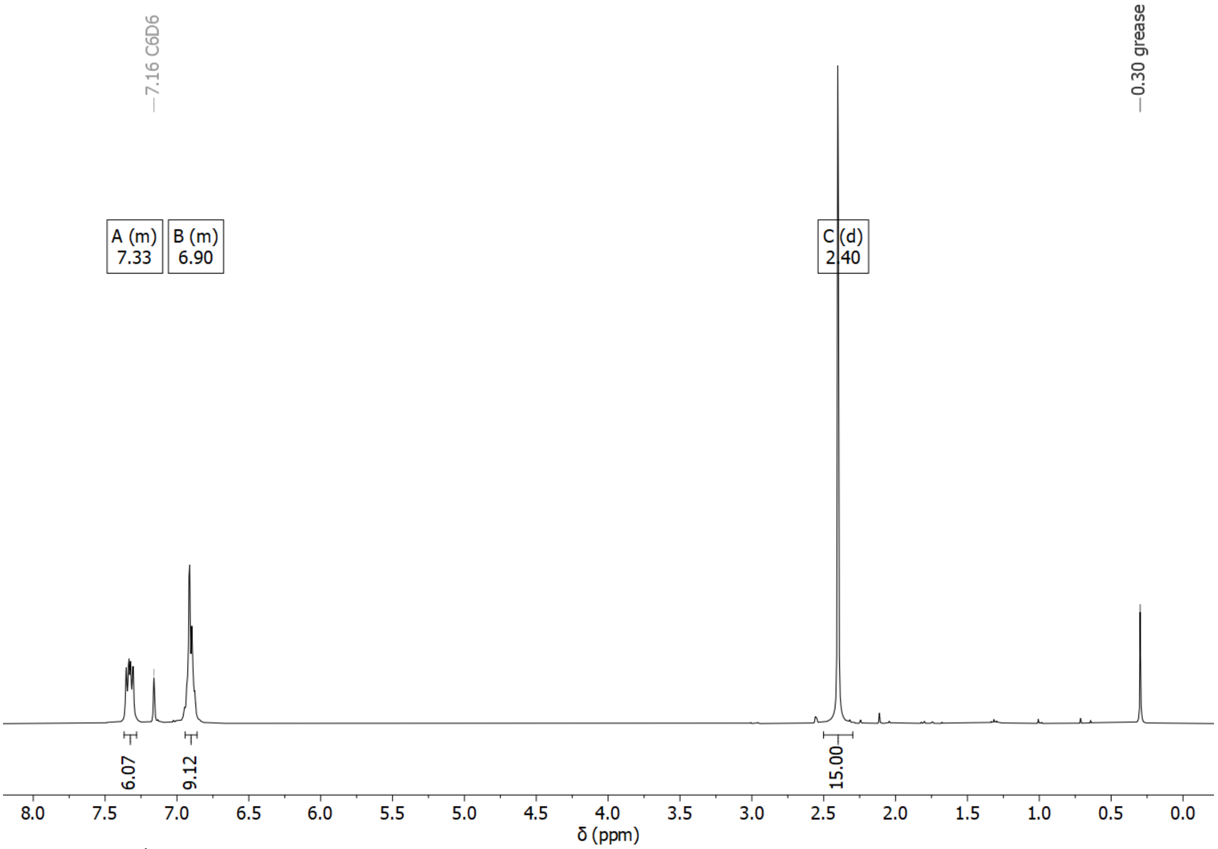

Figure S50: ^{1}H-NMR spectrum of $(PPh_3)AuCp^*$.

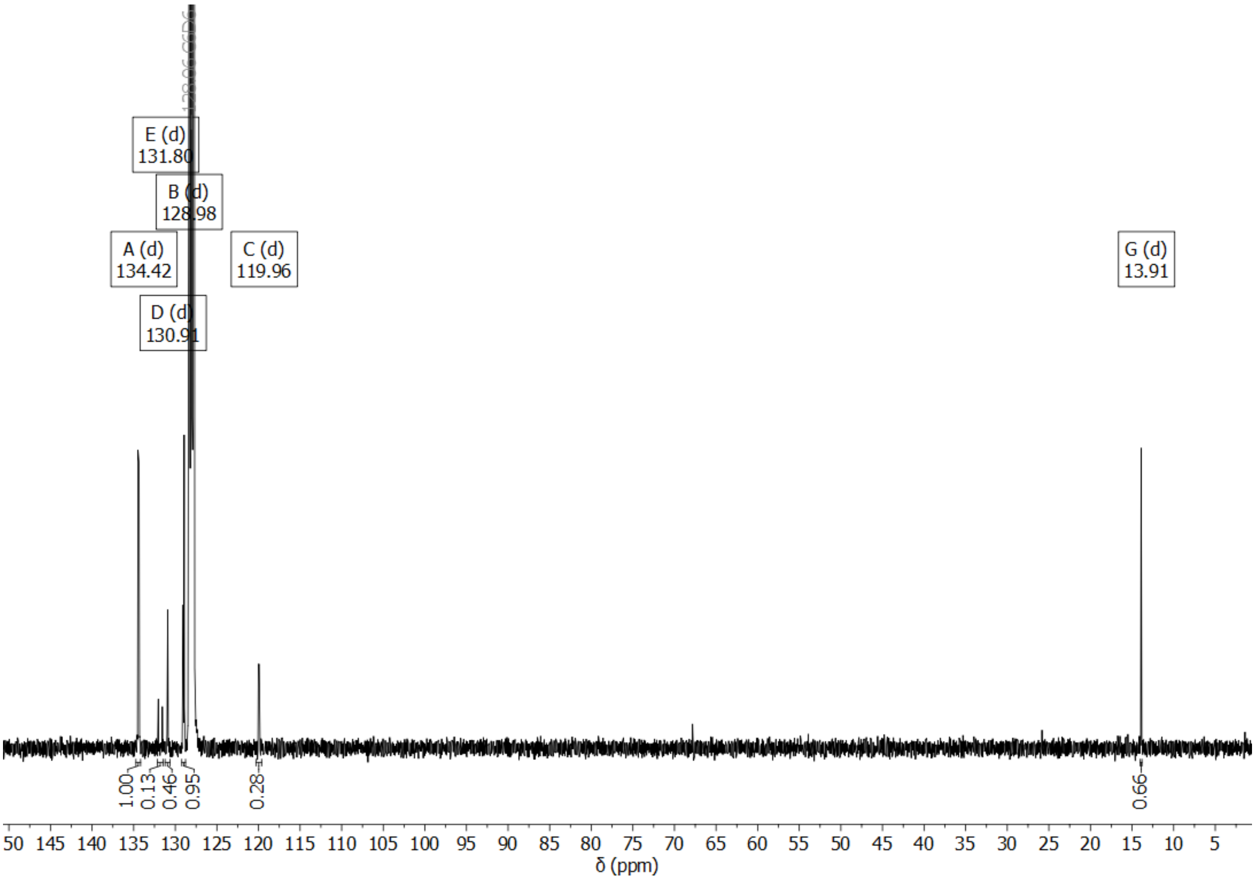

Figure S51: $^{13}C\{^{1}H\}$-NMR spectrum of $(PPh_3)AuCp^*$

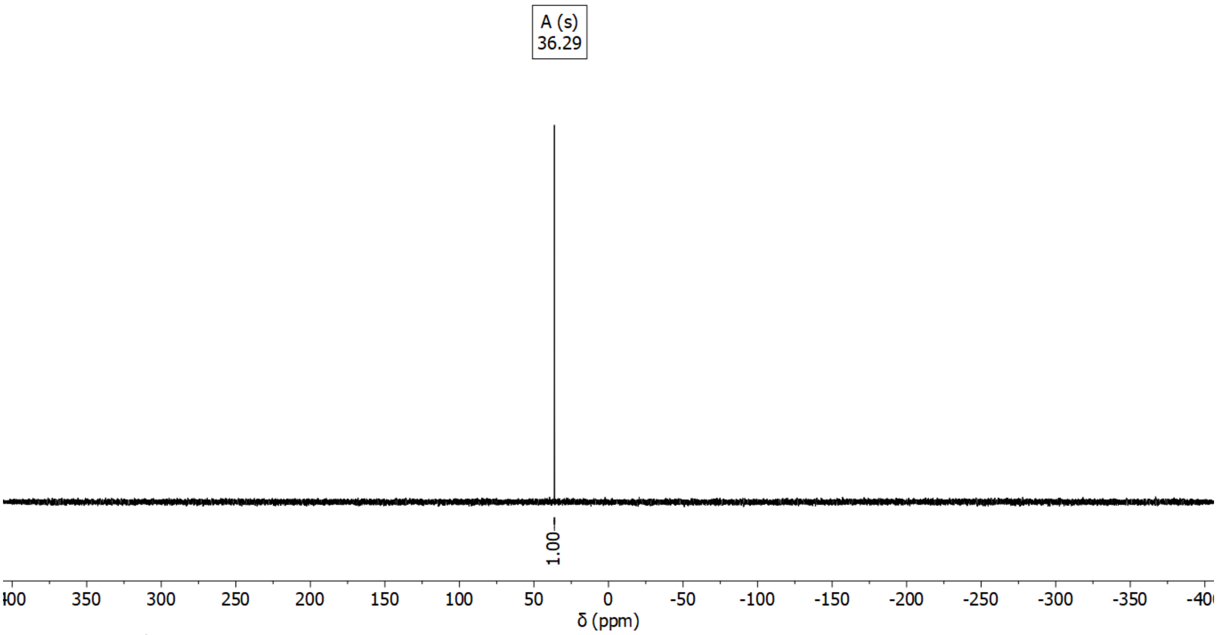

Figure S52: $^{31}P\{^{1}H\}$-NMR spectrum of $(PPh_3)AuCp^*$.

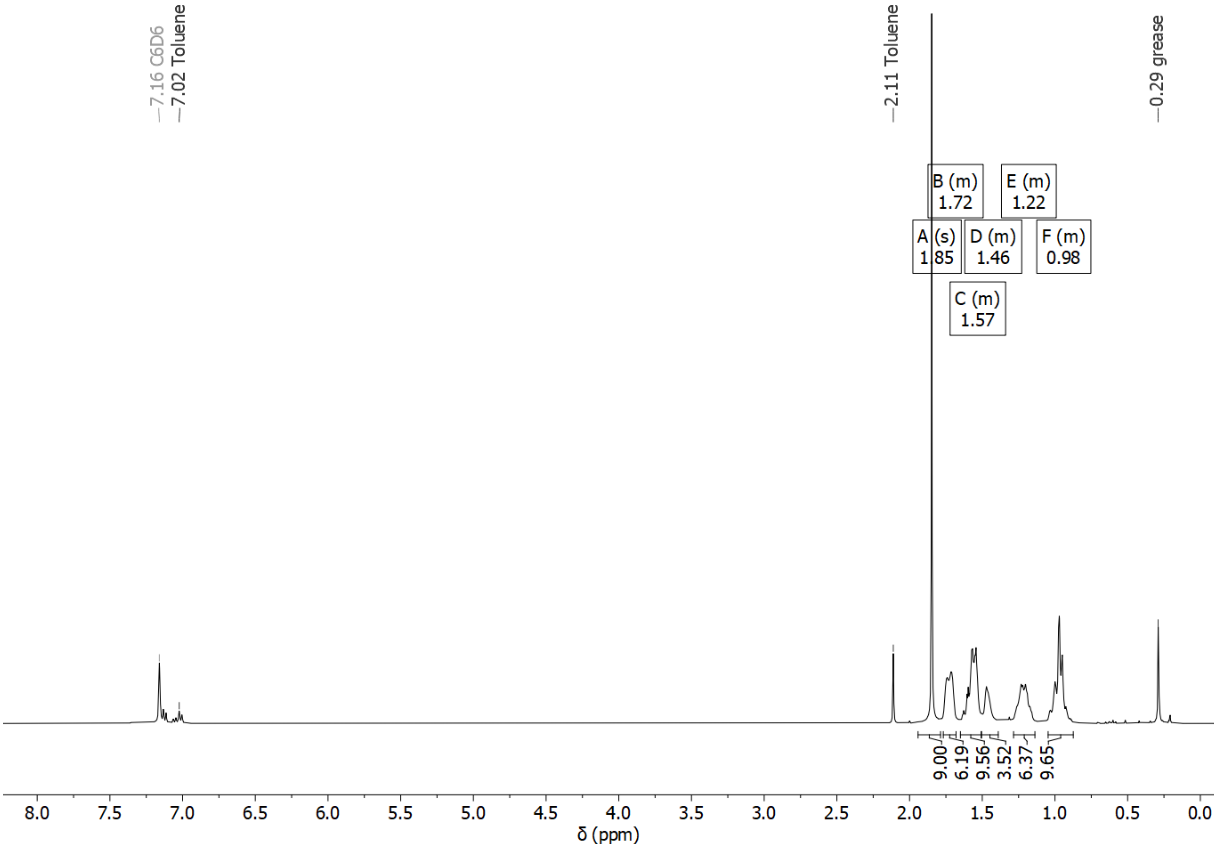

Figure S53: ^{1}H-NMR spectrum of $(PCy_3)AuO\textit{t}$-Bu

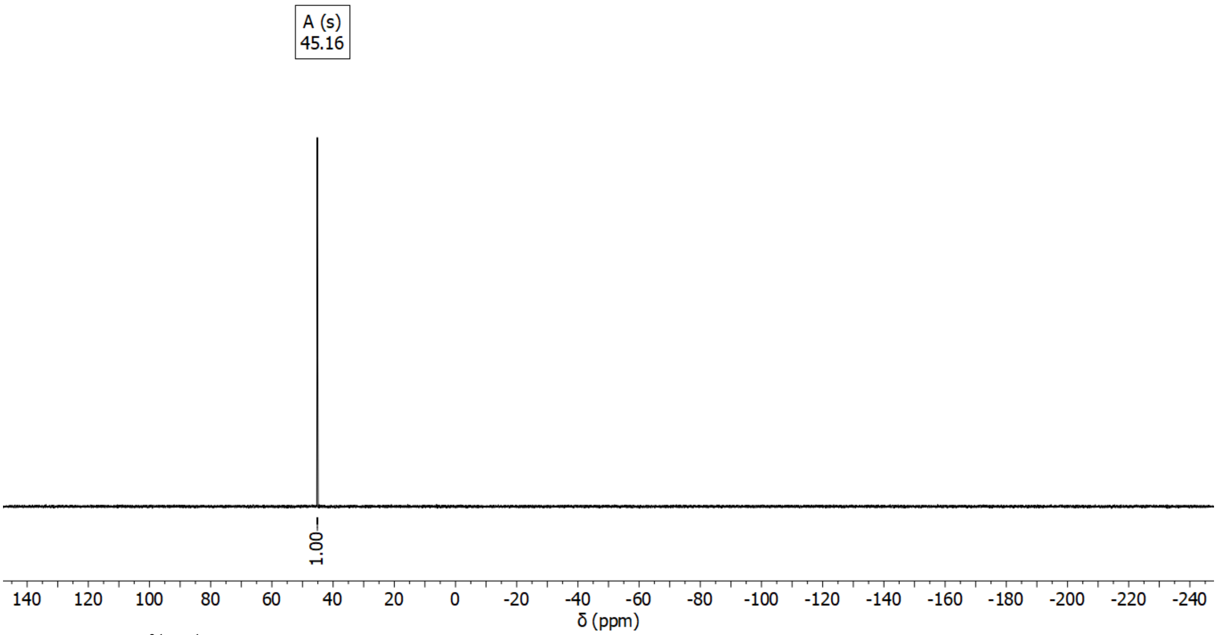

Figure S54: $^{31}P\{^{1}H\}$-NMR spectrum of (PCy$_3$)AuO*t*-Bu.

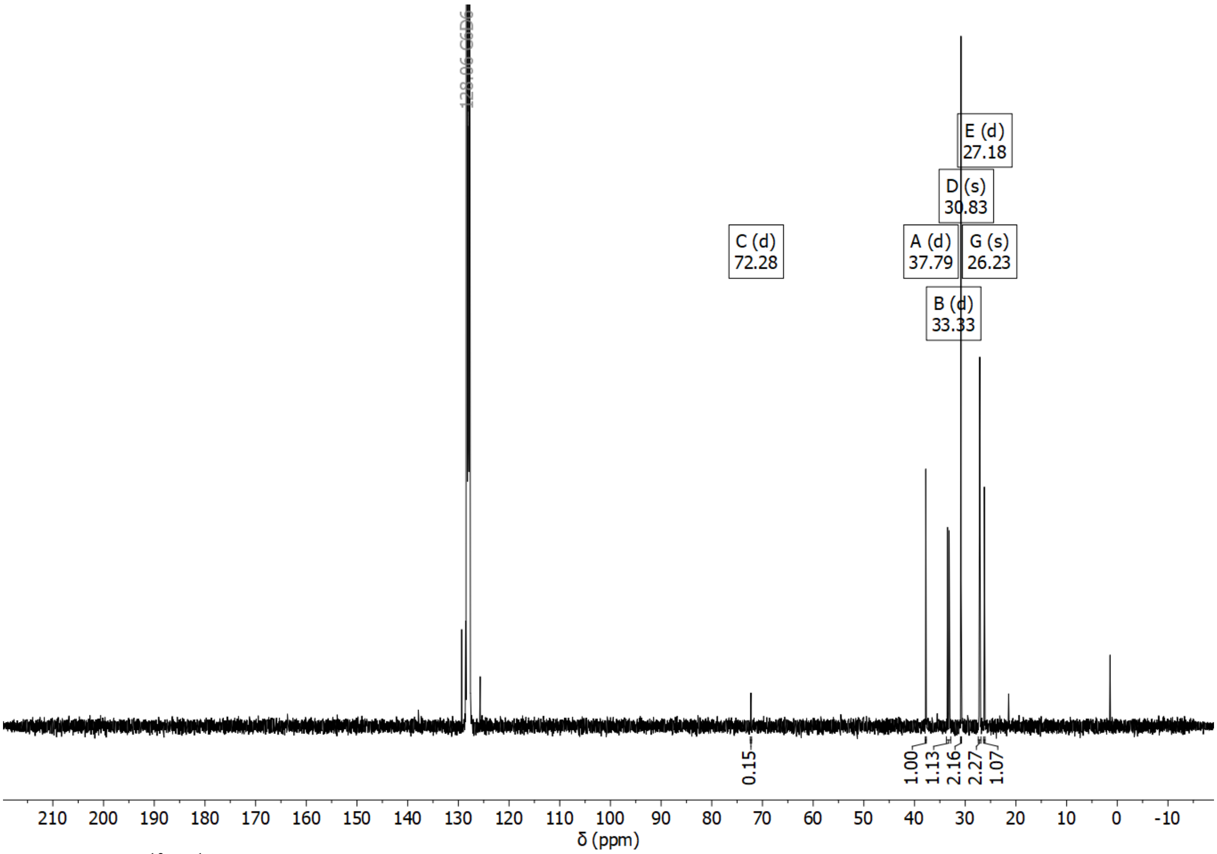

Figure S55: $^{13}C\{^{1}H\}$-NMR spectrum of (PCy$_3$)AuO*t*-Bu.

.

5. Crystallography

SC-XRD data were collected on a Bruker D8-Venture single crystal x-ray diffractometer equipped with a CMOS detector (Bruker APEX IV, κ-CMOS), a TXS rotating anode with MoK_{α} radiation (λ = 0.71073 Å) and a Helios optic using the Bruker APEX4 software package.[4] Single crystals were coated with perfluorinated ether, fixed on top of a micro sampler and frozen under a stream of cold nitrogen. Reflections were corrected for Lorentz and polarisation effects, scan speed, and background using SAINT.[5] Absorption correction, including odd and even ordered spherical harmonics was performed using SADABS.[6] The structures were solved using SHELXT with the aid of successive difference Fourier maps, and were refined against all data using SHELXL-2014/2017 in conjunction with SHELXLE.[7-9] Hydrogen atoms were calculated in ideal positions as follows: Methyl hydrogen atoms were refined as part of rigid rotating groups, with a C–H distance of 0.98 Å and $U_{iso}(H) = 1.5 \cdot U_{eq}(C)$. Other H atoms were placed in calculated positions and refined using a riding model, with methylene and aromatic C–H distances of 0.99 Å and 0.95 Å, respectively, other C–H distances of 1.00 Å, all with $U_{iso}(H) = 1.2 \cdot Ueq(C)$. Full-matrix least-squares refinements were carried out by minimizing $\Sigma w(F_o^2 - F_c^2)^2$ with the SHELXL weighting scheme. Neutral atom scattering factors for all atoms and anomalous dispersion corrections for the non-hydrogen atoms were taken from the *International Tables for Crystallography*.[10] A split layer refinement was used for disordered groups and additional restraints on distances, angles and anisotropic displacement parameters were employed to ensure convergence within chemically reasonable limits, if necessary.[8] Images of the crystal structures were generated with *Mercury* and *Povray*.[11] CCDC 2219636 contains the supplementary crystallographic data for $[Au_2Al_5](Cp^*)_5$ (**3**). These data can be obtained free of charge from The Cambridge Crystallographic Data Centre via www.ccdc.cam.ac.uk/structures.

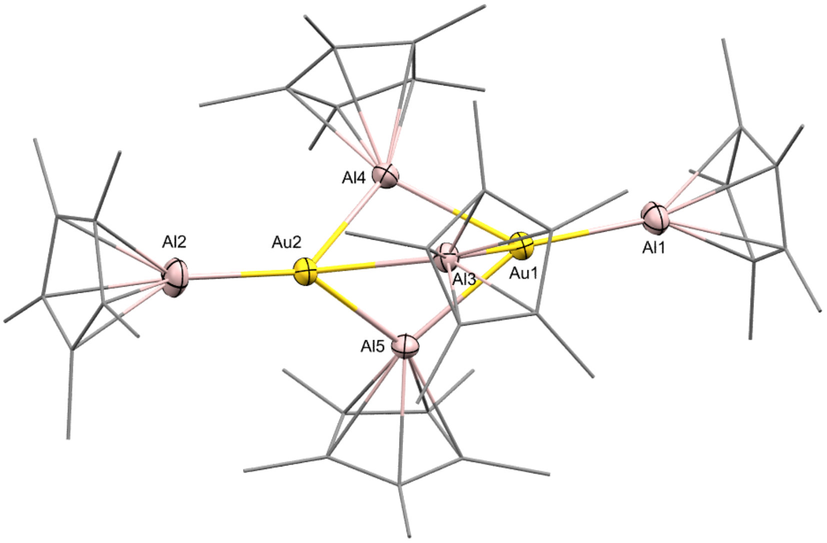

Figure S56: Molecular structure of $[Au_2Al_5](Cp^*)_5$ (**3**). Au: yellow, Al: pink, C: grey, H-atoms and co-crystallized solvents are omitted for clarity. Thermal ellipsoids are shown at the 50 % probability level.

Table S1: Crystallographic information for the compound **3**

Chemical formula	$C_{57}H_{83}Al_5Au_2$	
Formula weight	1297.06	
Temperature	100(2) K	
Wavelength	0.71073 Å	
Crystal size	0.113 x 0.155 x 0.314 mm	
Crystal system	monoclinic	
Space group	P 1 21/n 1	
Unit cell dimensions	a = 11.5893(9) Å	α = 90°
	b = 37.149(3) Å	β = 104.188(3)°
	c = 13.9089(11) Å	γ = 90°
Volume	5805.6(8) $Å^3$	
Z	4	
Density (calculated)	1.484 g/cm^3	
Absorption coefficient	5.157 mm^{-1}	
F(000)	2592	
Diffractometer	Bruker D8 Venture	
Radiation source	TXS rotating anode, Mo	
Theta range for data collection	1.89 to 25.91°	
Index ranges	-14<=h<=14, -45<=k<=45, -17<=l<=17	
Reflections collected	213543	
Independent reflections	11270 [R(int) = 0.0460]	
Coverage of independent reflections	99.8%	
Max. and min. transmission	0.7453 and 0.5437	
Structure solution technique	direct methods	
Structure solution program	SHELXS-97 (Sheldrick, 2008)	
Refinement method	Full-matrix least-squares on F^2	
Refinement program	SHELXL-2014/7 (Sheldrick, 2015)	
Function minimized	$\Sigma w(F_o^2 - F_c^2)^2$	
Data / restraints / parameters	11270 / 75 / 639	
Goodness-of-fit on F^2	1.291	
Δ/σ_{max}	0.002	
Final R indices	10649 data; I>2σ(I)	R1 = 0.0306, wR2 = 0.0665
	all data	R1 = 0.0334, wR2 = 0.0678
Weighting scheme	$w=1/[\sigma^2(F_o^2)+27.8757P]$ where $P=(F_o^2+2F_c^2)/3$	
Largest diff. peak and hole	2.192 and -1.850 $eÅ^{-3}$	
R.M.S. deviation from mean	0.103 $eÅ^{-3}$	

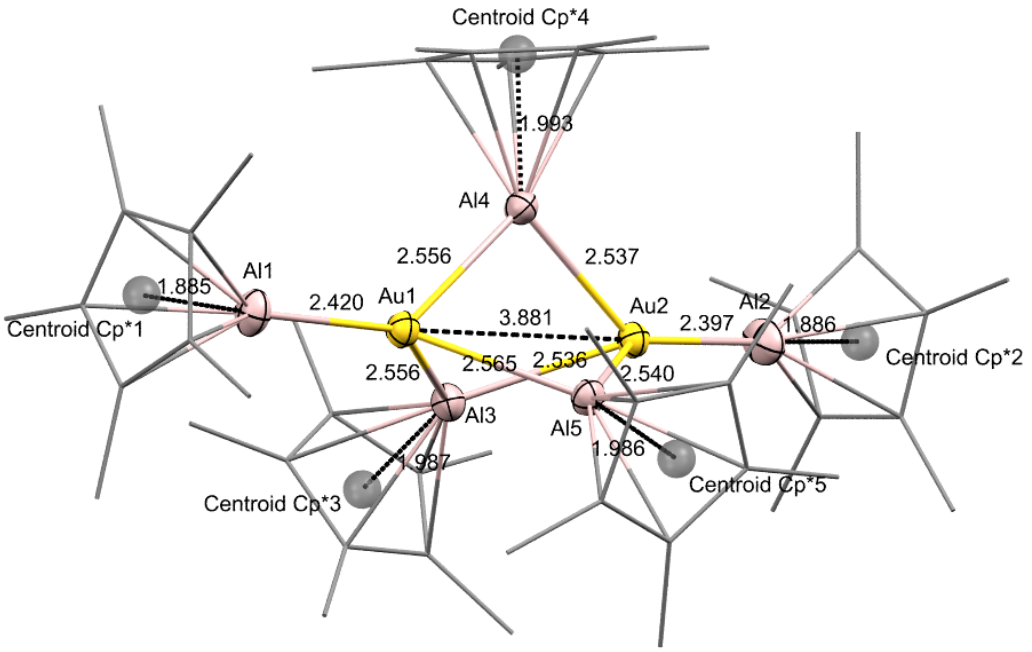

Figure S57: Selected bond lengths and atom distances in the structure of **3**, showing a large Au-Au distance of 3.881 Å (Au1-Au2) and a short Al-Au bond length of 2.397 Å.

Table S2: Selected bond distances for compound **3**.

Atoms	Bond distances [Å]
Au1–Al1	2.420
Au1–Al3	2.556
Au1–Al4	2.556
Au1–Au5	2.565
Au2–Al2	2.397
Au2–Al3	2.536
Au2–Al4	2.537
Au2–Al5	2.540
Au1–Au2	3.881
Al1–Centroid Cp*1	1.885
Al2–Centroid Cp*2	1.886
Al3–Centroid Cp*3	1.987
Al4–Centroid Cp*4	1.993
Al5–Centroid Cp*5	1.986

Table S3: Selected bond angles for compound **3**.

Atoms	Bond angles [°]
Al1–Au1–Al3	138.27
Al1–Au1–Al4	136.46
Al1–Au1–Al5	144.41
Al3–Au1–Al4	68.51
Al3–Au1–Al5	67.46
Al4–Au1–Al5	67.98
Al2–Au2–Al3	135.67
Al2–Au2–Al4	137.79
Al2–Au2–Al5	144.36
Al3–Au2–Al4	69.13
Al3–Au2–Al5	68.14
Al4–Au2–Al5	68.65
Al1–Au1–Au2	175.17
Al2–Au2–Au1	174.69

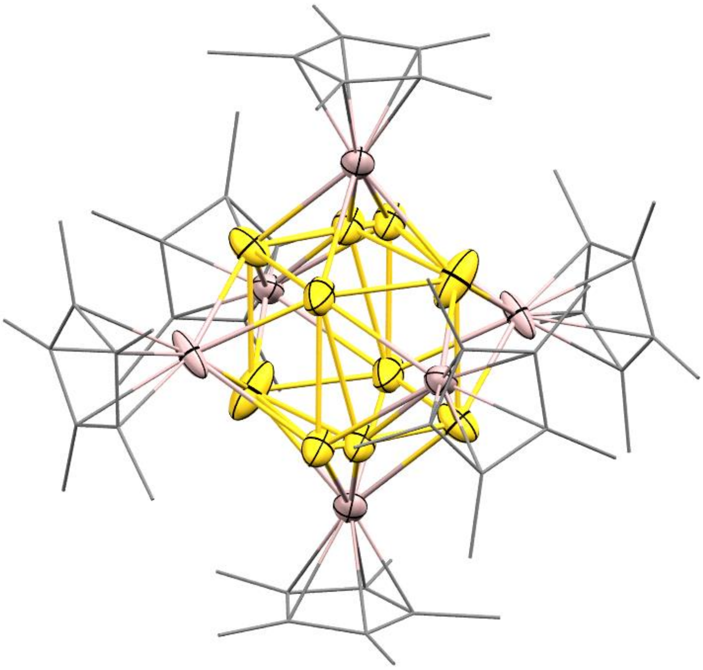

Figure S58: Molecular structure of $[HAu_7Al_6](Cp^*)_6$. Au: yellow, Al: pink, C: grey; H-atoms, co-crystallized solvents and disordered ligand moieties are omitted for clarity. Note that the occupancy of gold atoms within the cluster core cannot be assigned unambiguously.

The best structure model we obtained so far from X-ray single crystal data unfortunately contains several unsatisfying features like elongated displacement ellipsoids for one independent Au and one Al atom, respectively, and a split position for another Au atom, all this termed as disorder as usually done. A refinement with split positions for all affected Au and Al atoms was not successful, while the Cp* ligand corresponding to the respective Al atom could be refined at four split positions. This whole disorder can be roughly, but not completely, interpreted as a static superposition of two orientations of the cluster shown in Figure 2(2), however, dynamic interconversion

between different shapes, as suggested by the DFT calculations, must be taken into account. For a phase transition between temperatures of synthesis and measurement we could not detect any hints. A measurement at higher temperature was not possible because the crystals de-compose upon loss of solvent molecules of crystallization.

Table S4: Crystallographic information for $[HAu_7Al_6](Cp^*)_6$.

Chemical formula	$C_{74}H_{106}Al_6Au_8$	
Formula weight	2733.19	
Temperature	100(2) K	
Wavelength	0.71073 Å	
Crystal size	0.113 x 0.155 x 0.314 mm	
Crystal system	monoclinic	
Space group	*C* 1 2/m 1	
Unit cell dimensions	a = 19.9350(12) Å<br>b = 17.5005(9) Å<br>c = 12.9731(7) Å	α = 90°<br>β = 124.724(2)°<br>γ = 90°
Volume	3719.9(4) $Å^3$	
Z	2	
Density (calculated)	2.440 g/cm^3	
Absorption coefficient	15.823 mm^{-1}	
F(000)	2520	
Diffractometer	Bruker D8 Venture Duo IMS	
Radiation source	IMS microsource, Mo	
Theta range for data collection	1.96 to 25.44°	
Index ranges	-24<=h<=24, -21<=k<=21, -15<=l<=15	
Reflections collected	32825	
Independent reflections	3539 [R(int) = 0.0829]	
Coverage of independent reflections	99.5%	
Structure solution technique	direct methods	
Structure solution program	SHELXS-97 (Sheldrick, 2008)	
Refinement method	Full-matrix least-squares on F^2	
Refinement program	SHELXL-2014/7 (Sheldrick, 2015)	
Function minimized	$\Sigma w(F_o^2 - F_c^2)^2$	
Data / restraints / parameters	3539 / 0 / 121	
Goodness-of-fit on F^2	1.085	
Δ/σ_{max}	0.359	
Final R indices	2565 data; I>2σ(I)<br>all data	R1 = 0.1135,<br>wR2 = 0.2469<br>R1 = 0.1586,<br>wR2 = 0.2655
Weighting scheme	$w=1/[\sigma^2(F_o^2)+(0.0669P)^2+774.2982P]$<br>where $P=(F_o^2+2F_c^2)/3$	
Largest diff. peak and hole	7.046 and -6.240 $eÅ^{-3}$	
R.M.S. deviation from mean	0.422 $eÅ^{-3}$	

6. DFT-Calculations

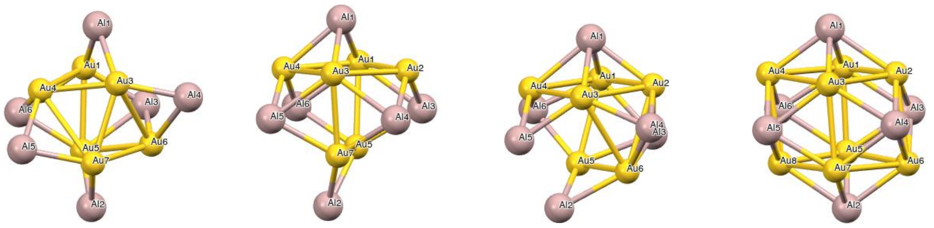

	(a)	(b)	(c)	(d)
ΔE (eV)	+0.62	+0.05	0.00	
ΔG (eV)	+0.56	+0.15	0.00	
$\Delta E_{HOMO/LUMO}$ (eV)	1.32	1.29	1.96	1.44

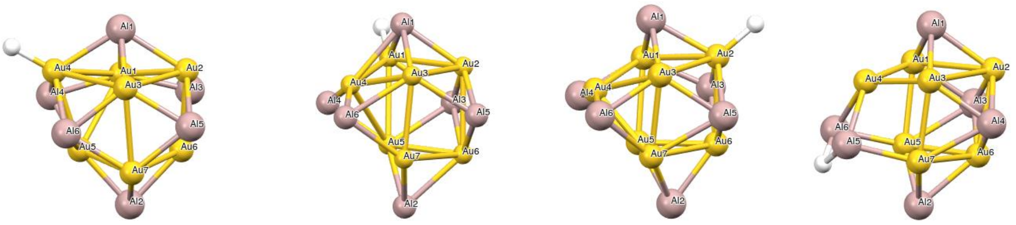

	(e)	(f)	(g)	(h)
ΔE (eV)	0.00	+0.02	+0.14	+0.17
ΔG (eV)	0.00	+0.11	+0.21	0.00
$\Delta E_{HOMO/LUMO}$ (eV)	1.40	1.42	1.57	1.69
^{1}H NMR (ppm)	8.7	8.6	5.7	4.3
^{1}H(Me) NMR (ppm)	1.8	1.8	1.8	1.9
^{13}C(Cp*) NMR (ppm)	116.8	116.9	116.9	117.9
^{13}C(Me) NMR (ppm)	10.9	10.8	10.8	10.9
	ν(Au_4-H)=1745 cm^{-1}	ν(Au_1-H)=1742 cm^{-1}	ν(Au_2-H)=1729 cm^{-1}	ν(Al-H)=1143 cm^{-1}

Figure S59: DFT-optimized low-energy structures of $[Au_6Al_6](Cp^*)_6$ (**1**) (a, b, c), $[Au_8Al_6](Cp^*)_6$ (d) and $[HAu_7Al_6](Cp^*)_6$ (**2**) (e, f, g, h).

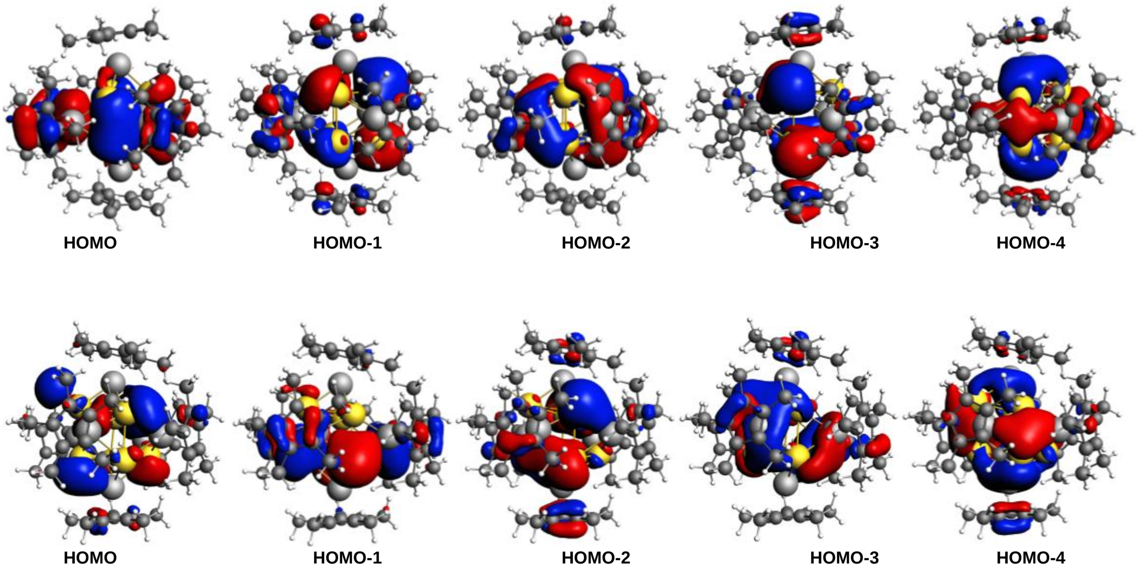

Figure S60: The five highest occupied Kohn-Sham orbitals of the lowest isomers of : Top: $[Au_6Al_6](Cp^*)_6$ (**1**, see Figure S59 c); bottom: $[HAu_7Al_6](Cp^*)_6$ (**2**, see Figure S59 e). In both cases, they can be identified as composing the cluster superatomic 1D set.

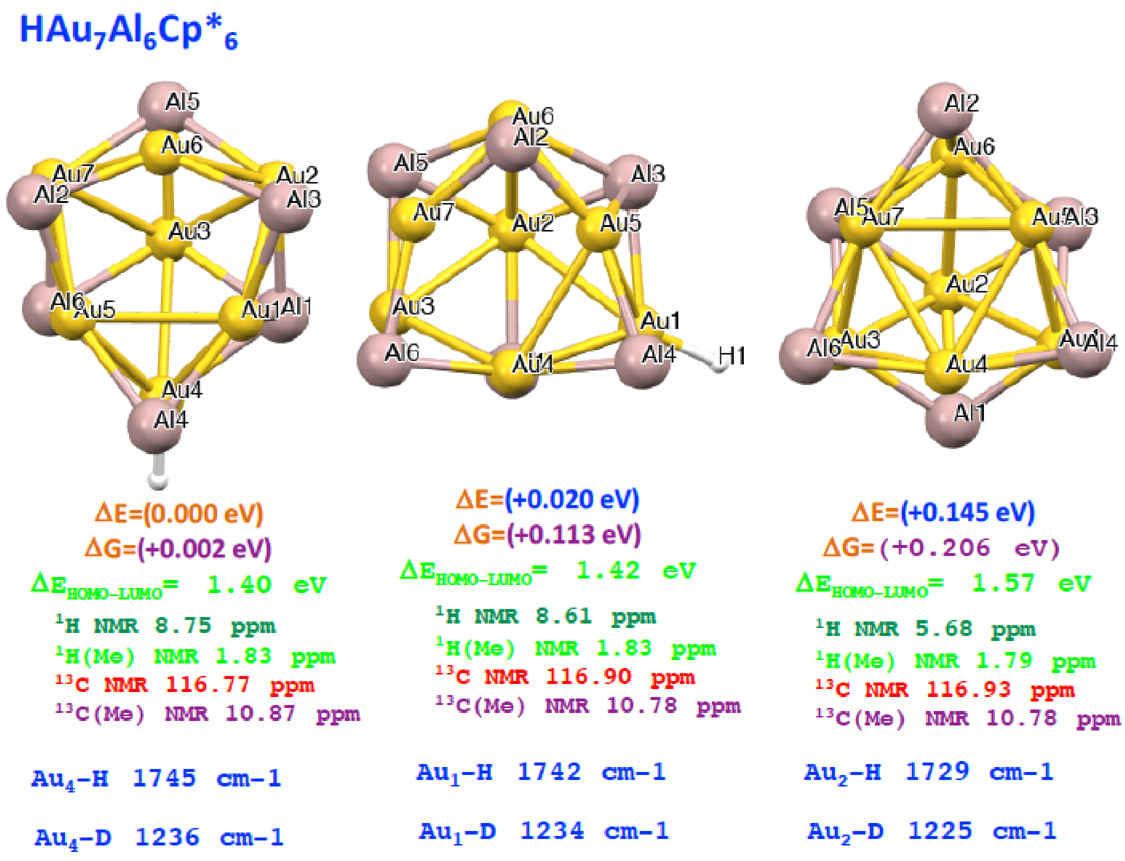

Figure S61: DFT-optimized low-energy structures of $[HAu_7Al_6](Cp^*)_6$ (**2**) and $[DAu_7Al_6](Cp^*)_6$ (**2D**) as well as their calculated spectroscopic data.

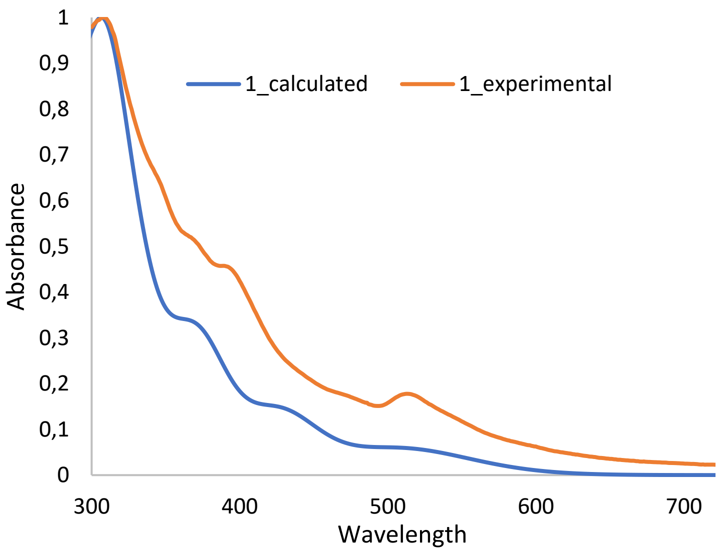

Figure S62: The TD-DFT simulated UV-vis spectrum of **1** compared with the experimental.

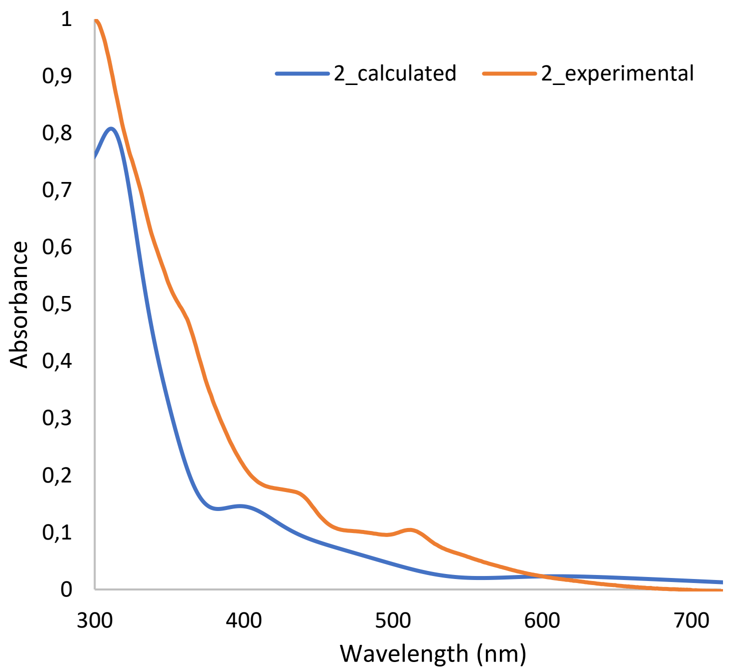

Figure S63: The TD-DFT simulated UV-vis spectrum of **2** compared with the experimental**.**

7. References

(1) Tsui, E. Y.; Müller, P.; Sadighi, J. P. Reactions of a Stable Monomeric Gold(I) Hydride Complex. *Angewandte Chemie International Edition* **2008**, *47* (46), 8937-8940.
(2) Ganesamoorthy, C.; Loerke, S.; Gemel, C.; Jerabek, P.; Winter, M.; Frenking, G.; Fischer, R. A. Reductive elimination: a pathway to low-valent aluminium species. *Chemical Communications* **2013**, *49* (28), 2858-2860.
(3) Brown, H. C.; Krishnamurthy, S.; Hubbard, J. L. Addition compounds of alkali metal hydrides. 15. Steric effects in the reaction of representative trialkylboranes with lithium and sodium hydrides to form the corresponding trialkylborohydrides. *Journal of the American Chemical Society* **1978**, *100* (11), 3343-3349.
(4) Werner, H.; Otto, H.; Ngo-Khac, T.; Burschka, C. Synthese und eigenschaften neur kupfer- und gold-komplexe des typs C5H5MPR3, C5Me5MPR3 und R″C2MPR3 (M = Cu, Au) sowie die kristallstruktur von C5H5AuPPr3i. *Journal of Organometallic Chemistry* **1984**, *262* (1), 123-136.

(5) APEX4 Version 2021.10-0, Bruker AXS Inc., Madison, Wisconsin, USA, 2021.
(6) SAINT, Version 8.34 A, Bruker AXS Inc., Madison, Wisconsin, USA, 2014.
(7) G. M. Sheldrick, SADABS, Version 2014/5, Bruker AXS Inc., Madison, Wisconsin, USA, 2014.
(8) G. M. Sheldrick, SHELXT - Integrated space-group and crystal-structure determination. *Acta Crystallogr. Sect. A* **2015,** *71* (1), 3-8.
(9) G. Sheldrick, Crystal structure refinement with SHELXL. *Acta Crystallogr. Sect. C* **2015,** *71* (1), 3-8.
(10) C. B. Hübschle, G. M. Sheldrick, B. Dittrich, ShelXle: a Qt graphical user interface for SHELXL. *J. Appl. Crystallogr.* **2011,** *44* (6), 1281-1284.
(11) *International Tables for Crystallography, Vol. C*. Wilson, A. J., Ed. Kluwer Academic Publishers: Dordrecht, The Netherlands, **1992**; pp Tables 6.1.1.4 (pp. 500–502), 4.2.6.8 (pp. 219–222), and 4.2.4.2 (pp. 193–199).
(12) C. F. Macrae, I. J. Bruno, J. A. Chisholm, P. R. Edgington, P. McCabe, E. Pidcock, L. Rodriguez-Monge, R. Taylor, J. van de Streek, P. A. Wood, Mercury CSD 2.0 - new features for the visualization and investigation of crystal structures, *J. Appl. Crystallogr.* **2008,** *41* (2), 466-470.
